# Stakeholder and end-user perspectives to refine the Context-sensitive Positive Health Questionnaire (CPHQ) to measure broad health

**DOI:** 10.1371/journal.pone.0330014

**Published:** 2026-08-24

**Authors:** Mirte Boelens, Cheryl Roumen, Esther J. Bloemen-van Gurp, John Dierx, Lenny Nahar-van Venrooij, Tim van Zutphen, Pien M.R. Christiaanse, Jaron Schnitzer, M. Elske van den Akker-van Marle, Miriam J.J. de Kleijn, Marja van Vliet, Jessica C. Kiefte-de Jong, Marieke D. Spreeuwenberg

**Affiliations:** 1 Leiden University Medical Center/Health Campus The Hague, Department of Public Health and Primary Care, The Hague, The Netherlands; 2 Department of Health Services Research, Care and Public Health Research Institute (CAPHRI), Faculty of Health Medicine and Life Sciences, Maastricht University, Maastricht, The Netherlands; 3 Zuyd University of Applied Sciences, Research Center of community care, Heerlen, The Netherlands; 4 Fontys University of Applied Sciences, Center of Expertise Health, Eindhoven, The Netherlands; 5 Avans University of Applied Science, Centre of Expertise Perspective in Health, Research Group Equal Chances on Healthy Choices, Breda, Netherlands; 6 Jeroen Bosch Academy Research, Jeroen Bosch Hospital, ‘s-Hertogenbosch, Netherlands; 7 Tranzo Scientific Centre for Care and Wellbeing, Tilburg University, Tilburg, the Netherlands; 8 Department of Sustainable Health, Faculty Campus Fryslân, University of Groningen, Leeuwarden, the Netherlands; 9 Department of Biomedical Data Sciences, Section of Medical Decision-Making, Leiden University Medical Center, Leiden, The Netherlands; 10 Institute for Positive Health, Utrecht, The Netherlands; Hunan University, CHINA

## Abstract

**Introduction:**

Broad health concepts are increasingly embedded in healthcare and other domains. Therefore, in a previous study, we developed and validated the Context-Sensitive Positive Health Questionnaire (CPHQ). The CPHQ is based on Positive Health, the Capability Approach and on perspectives of stakeholders and end-users, including those with a lower socioeconomic position (SEP) with the aim to validly measure broad health. Stakeholders and end-users were involved in the development process. However, professionals working in healthcare, welfare or policy domain were not sufficiently involved, while their perspective on how to measure broad health is needed to support broader applicability and acceptability.

**Objective:**

We aimed to refine the 32-item CPHQ for cross-domain use through focus group discussions (FGDs) with stakeholders across healthcare, welfare and policy domains, researchers, as well as patients and citizens, including those from a lower SEP, expert review and a member check.

**Methods:**

We conducted nine (FGDs) and one interview with stakeholders from healthcare, welfare, and policy domains, research, patients and (lower SEP) citizens (n = 76). An adapted COMET method analysis and qualitative analysis were used, followed by a member check to validate the changes.

**Results:**

Items were omitted, merged or added, leading to a refined CPHQ (28 items). Main themes guiding edits were 1) the importance of avoiding normative phrasing and including subjectively interpretable items, 2) prioritization of items reflecting aspects of experienced health (e.g., “I feel happy” or “I feel healthy”), 3) relevance of functionings. At the same time, views diverged on the inclusion and wording of items related to exclusion, political representation and sense of belonging. These items were subsequently discussed by the core group and evaluated using the predefined decision rules and qualitative feedback.

**Conclusion:**

The CPHQ refinement involved a wide range of stakeholders, including end-users. This led to a 28-item CPHQ to measure broad health. Future research in different populations and settings is needed to validate and possibly shorten the refined CPHQ.

## Introduction

There is growing recognition that health encompasses more than the absence of disease. In response to critiques of the World Healthcare Organization’s (WHO) definition of health, which is the complete state of physical, mental and social well-being, several approaches to measure broad health have been proposed [1]. In 2011, Huber proposed a more dynamic conceptualization of health as “the ability to adapt and to self-manage in the face of life’s physical, emotional, and social challenges” [2]. This was operationalized into the concept of Positive Health, which reflects health as a broad construct comprising six dimensions: bodily functions, mental well-being, meaningfulness, quality of life, participation, daily functioning. Positive Health is about what people themselves perceive to constitute their health, and what gives meaning to their lives to enable self-management and resilience [3]. The Capability Approach, as developed by Sen and Nussbaum, differs from Positive Health as it is a dynamic and context-sensitive framework by focusing on individuals’ opportunities to achieve what they value in terms of health [4,5]. The Capability Approach distinguishes between actual states of health called “functionings” (e.g., feeling healthy or feeling happy) and “capabilities” that enable people to achieve these states of health. Health, in this view, is not only defined by actual outcomes, but also by the capabilities to attain them. The conversion of resources into capabilities depends on “conversion factors”. “Conversion factors” can be personal (e.g., sex or reading skills), social (e.g., public policies, social norms or discriminating practices) or environmental (e.g., climate or geographical region) [6,7].

Within Dutch healthcare policy, there is a growing emphasis on health as a broad conceptualization of health in which the ability to adapt is included [2]. We will refer to the broad conceptualization of health, including Positive Health and the Capability Approach, as “broad health”. Accordingly, policy documents and health programs within the healthcare and social domain have been established in line with this framework. However, so far, no adequately validated questionnaires seem to be available to evaluate the impact of these health programs and interventions [8–11]. Although several previous attempts have been proposed some limitations remain either with psychometric validation or because the contextual factors are not sufficiently included.

the 42-item My Positive Health questionnaire, originally developed as a dialogue tool (https://www.iph.nl/en/positive-health/conversation-tools/), was not suitable as a validated measurement instrument as it lacks comprehensibility and discriminant validity [12,13]. Subsequently, this questionnaire was reordered and reduced to a 17-item questionnaire using factor analysis [13]. The 17-item questionnaire showed adequate psychometric properties but is primarily focused on perceived health whereas in line with current policy and intervention approaches, and as suggested by the International Union for Health Promotion and Education (IUHPE), it would be more valuable to develop a measurement instrument based on Positive Health, supplemented with contextual factors such as political, environmental, and social aspects [13,14]. Additionally, a measurement instrument developed in this way is expected to align well with current Dutch governmental policies and intervention approaches within the healthcare and welfare and policy settings. As a result, it is likely to be both sensitive and responsive in capturing the impact of health interventions.

Therefore, in a previous study, we developed and validated a context-sensitive Positive Health questionnaire (CPHQ) as a measurement instrument based on the principles of Positive Health and grounded in the Capability Approach by accounting for differences in capabilities and conversion factors influencing health [15]. This CPHQ consists of 32 items and comprises 11 dimensions; relaxation, autonomy, fitness, perceived environmental safety, exclusion, social support, financial resources, political representation, health literacy, resilience, and enjoyment. This CPHQ demonstrated adequate factorial and concurrent validity. However, in the development of the CPHQ a broad range of potential users such as professionals working in the healthcare, welfare and policy sector were not sufficiently involved in the initial questionnaire development. They were consulted after focus group discussions (FGDs) with patients and citizens were finished. Taking into account their view on how to measure broad health is needed to support applicability and acceptance in different sectors. Thus, to improve the applicability and acceptance of the CPHQ, further refinement based on the perspective of a more diverse group of stakeholders is needed.

The objective of this study is to further refine the CPHQ-instrument to make it more generally applicable and therefore accepted across different domains through FGDs incorporating the perspectives of a broader group of stakeholders across healthcare, welfare and policy domains, researchers, as well as patients and citizens, including those from a lower SEP, expert review and a member check.

## Materials and methods

For the refinement of the CPHQ measurement instrument different steps were conducted. Step 1 consisted of FGDs with stakeholders and end users (patients and (lower SEP) citizens). Step 2 consisted of an expert review by the core group and the Dutch National Positive Health research Network and member check after analysis of the FGDs. Both steps are described in more detail below. As a starting point for the current development, the CPHQ consisting of 32 items was used. This report follows the standards for reporting qualitative research as described by The Standards for Reporting Qualitative Research (SRQR) [16].

The core group (MS, JK, MB, CR, EB, JD) consisted of six project members with diverse research backgrounds: a researcher with a background in psychology and statistics, a researcher with a background in population health, a researcher in the field of broad health concepts, a researcher in the field of health behavior, health promotion and public health, a researcher with a background in nutritional sciences and public health, and a researcher with a background in participatory research and shared decision-making.

The Dutch National Positive Health research Network includes researchers from several Dutch universities, hospitals, the National Institute of Public Health and the Environment, the Institute of Positive Health, and VILANS a knowledge organization for care and support.

### Ethics

For this study, ethical approval was obtained from the Medical Ethics Review Committee of Leiden Den Haag Delft (LDD) (protocol 19035) which determined that the Medical Research Involving Human Subjects Act was not applicable. All participants gave written informed consent prior to study participation.

### Step 1: FGDs

#### Preparation for FGDs.

Before the FGDs were organized, first a rapid literature search was conducted to identify other potential questionnaire items to add to our starting point; the CPHQ [15]. A search was conducted in both PubMed and Google Scholar with the search string: (“health” OR“Positive Health”) AND “measurement”. Also, experts were consulted to provide suggestions for relevant questionnaires, such as the My Positive Health 42-item measurement scale [13], My Quality of Life Questionnaire [17], The subjective wellbeing-5 dimensions (SWB-5D) questionnaire [18], ICEpop CAPability measure for Adults (ICECAP-A) [19], Vita-16 [20], Brief Resilience Scale (BRS) [21], Personal Wellbeing Index- Adult (PWI-A) [22], EuroQol 5-dimensions (EQ-5D-5L) [23], Multicultural Quality of Life Index (MQLI) [24]. All questionnaire items yielded from the literature search were provided hard copy and online as a reference ‘handbook’ during the subsequent FGDs.

### Sampling and recruitment of FGD participants

Recruitment for the FGDs took place between 19-January-2023 and 19-September 2023. **Table 1** shows an overview of the recruitment. Ten FGDs (total of 76 participants) were organized at different locations within the Netherlands with different stakeholder groups. These groups were:

**Table 1:** Recruitment of focus group discussions (FGDs).

FGD	Target group	Distributor(s) of invitations	Invitations (n)	Participants (n)	Location	Date (dd-mm-yy)	Facilitators
1	Medical specialists and nurses	LNvV	15	10	Den Bosch	23-01-23	CR, EB, MB
2	Regional policymakers, welfare	JS, JD	33	14	Eindhoven	25-01-23	JD, MB, EB
3	Citizens	JS and passive via hard copy information letters in a primary care practice waiting room	18 + passive	8	Eindhoven	07-02-23	EB, MB
4	Lower SEP citizens	TvZ, PMRC	15	8	Leeuwarden	24-02-23	PMRC, TvZ, MB
5	National policymakers	JK, MS, JD, MdK, CR, EB and passive via social media	28 + passive	6	Utrecht	16-03-23	EB, MB
6	Researchers and experts	CR, EM, MB MdK, and passive via social media	33 + passive	8	Utrecht	01-03-23	CR, EB, MB
7	Paramedics	JS, MB and passive via social media	60 + passive	8	Eindhoven	23-03-23	JS, CR, EB, MB
8	Mixed group (policymakers and experts)	EB, MB, CR, MdK	17	6	Utrecht	06-04-23	EB, MB, CR
9	Patients	CR	7	1	Maastricht	16-08-23	CR, MB
10	Patients	TvZ, PMRC	72	7	Leeuwarden	19-09-23	PMRC, TvZ, MB

1) medical specialists and nurses (n = 10),
2) regional policymakers in health and welfare (n = 14),
3) citizens (n = 8),
4) citizens with a low socioeconomic position (n = 8),
5) national policymakers in health and welfare (n = 6),
6) researchers and experts (n = 8),
7) paramedics (n = 8),
8) mixed group of professional stakeholders who could not participate during the other FGDs (n = 6),
9) patients (two groups, n = 1), conducted as an interview,
10) patients (n = 7)

For one of the FGDs with patients, only one of the patients who agreed to participate showed up. This FGD was therefore approached as an interview using similar questions as the FGDs. A purposeful sampling approach was used. This meant that the participants for FGDs were recruited based on accessibility and based on a variety of professions and backgrounds and geographical areas to capture a wide range of perspectives. The distributors of the invitations were researchers who had access to the target population (see Table 1).

### FGD method

The 32 CPHQ items were presented in these FGDs on four posters (A0 size) (see S1 Appendix. FGD posters.). Items were subdivided over four posters according to the categories used in the original CPHQ, namely two posters with items concerning “perceived health” and two posters with items concerning “personal context”.

Firstly, participants received a short paper questionnaire including sociodemographic questions about their educational level, gender and work experience. FGD leaders helped participants to fill out the questionnaire if they had questions. The FGDs were divided into four parts: a full group starting discussion, round 1 in subgroups, round 2 in subgroups and a closing discussion with the full group (see S3 Appendix. Example prompts).

Full group starting discussion: First, the concept of broad health was defined and communicated to participants as: “The extent to which one is capable to adapt and to thrive given one’s physical, mental, social and contextual opportunities” [15]. Second, the discussion addressed topics as: what does health consist of, what do we need to capture when measuring health and preconditions of a measuring instrument.

Round 1: Participants in each FGD were divided into approximately equal subgroups of no more than five participants per subgroup. Each group was accompanied by a researcher that guided the process. The group size was limited to a maximum five participants in order to have sufficient time and opportunity for each individual of the group to state their opinion. Silent participants were encouraged to speak up by the researcher. Each group discussed the four posters with the original CPHQ items. First, qualitative feedback was requested through sticky notes on comprehensibility and relevancy of the item measuring broad health. During the FGDs, participants could consult the ‘handbook’ with items from the questionnaires that resulted from the rapid literature search to search for items that they felt were missing (See S2 Appendix. Hand book). Participants could also add their own items to the sticky notes.

Round 2: Individual quantitative feedback was requested on the importance of the item in measuring broad health through colored stickers. The colored stickers were: green = retain item (score 1), yellow = adjust item (score 2) and orange = omit item (score 3). Each participant received 32 colored stickers of each color (1 for each CPHQ question) and assigned these individually to each item on the four posters. Each participant also received a golden ticket that could be used to mark the most important item for them.

Full group closing discussion: After both rounds, the FGD was closed with a discussion among all participants of the FGD to ensure full diversity of opinions was taken into account. The discussions were mostly about the most striking findings and some additional discussion points related to the preferred number of items and answer method of a measurement instrument. An example topic list for the FGDs is included in S3 Appendix. Example prompts. FGDs took around 120 minutes and were audio recorded.

### Analysis of the FGDs

Descriptive analyses were performed for educational level, gender and work experience using IBM SPSS statistics for Windows, version 29.0 (International Business Machines Corporation, Armonk, New York).

FGDs were transcribed ad verbatim and analyzed independently by two researchers (CR and MB). Qualitative feedback on the sticky notes and the transcripts were analyzed deductively on the following themes: formulation, overlap, redundancy, importance, new suggestions, duration and length of the questionnaire and inductively using content analysis.

Analysis of the feedback of the colored stickers for was done using an adapted consensus method inspired by the COMET hand book and Blazeby et al. [25,26]. For each FGD all items were scored quantitatively by counting the colored stickers scored as 1 for green, 2 for yellow and 3 for orange. Number of golden tickets was scored per item. Decision rules were applied per item separately for each FGD. An item was retained when >50% scored 1 (green) and ≤15% 3 (orange), adjusted when >50% scored 2 (yellow) and ≤15% 1 (green), and omitted when >50% scored 3 (orange) and ≤15% 1 (green). The > 50% cutoffs were used to reflect equal weighing of each opinion and a democratic process of majority decision rather than plurality. The ≤ 15% cutoff was used to identify contrasting opinions compared to the 50% group, in order to investigate, discuss and form a substantiated opinion.

If an item score fell outside these predefined categories, the quantitative result was considered inconclusive and the item was subsequently discussed by the core group together using the quantitative results together with the qualitative input from the sticky notes and transcripts. When the quantitative results were close to one of the predefined categories (retain, adjust or omit), this was taken into consideration during discussion, but did not automatically determine the final decision. Final decisions were reached through consensus within the core group.

As one FGD with patients turned out as an interview, we combined this data with the other patient FGD data. This means all patients were analyzed together.

The resulting scores (retain, adjust and omit) were subsequently compared across all FGDs to determine whether consensus between FGDs had been reached. Consensus was defined as all FGDs resulting in the same quantitative decision (retain, adjust or omit) for a given item. In these cases, the consensus decision was followed by the core group. When decisions differed between FGDs, the item was discussed by the core group using the quantitative results, qualitative feedback from the transcripts and sticky notes and the golden ticket prioritization.

The goal of the qualitative feedback was not to examine emerging themes but rather to examine which aspects of the individual CPHQ items required adaptation or adjustment or removal. Therefore, all qualitative feedback was grouped all per individual CPHQ item across FGDs. Feedback was summarized by examining the frequency of recurring comments, for example that an item was difficult to understand or should be reformulated.

Items without consensus between FGDs were discussed by the core group. Consensus was achieved through structured discussion until agreement was reached. For each item, a different core group member initiated the discussion by stating their preferred decision and rationale. Subsequently, the remaining members shared their views in a rotating order. At least four out of the six core group members had to agree on a decision to retain, adjust or omit, merge an item.This structured discussion format was deliberately applied to ensure that all core group members had equal opportunity to contribute and to mitigate potential dominance effects or power imbalances within the group. CR and MB facilitated the discussion.

Items classified as adjust were reformulated based on the qualitative feedback. This could also include merging items together.

Finally items that were retained, adjusted and sometimes merged were further refined to improve readability, consistency and comprehensibility across the questionnaire.

Step 1 led to a revised CPHQ with a reduced set of items.

### Step 2: Expert validation and member check

#### Expert validation.

The revised CPHQ was validated with experts in two rounds: 1) with the core group together with experts involved in the research project 2) with experts from the Dutch National Positive Health research network. Finally, the core group discussed the input from these experts, and finally categorized the items into the components of the Capability Approach: resources, conversion factors, functionings, and capabilities rendering a revised CPHQ version [5].

#### Member check.

This refined CPHQ version was submitted to 55 of the original 76 participants of the FGDs as only these had agreed on being contacted for further research. Participants were asked to give feedback per e-mail on the readability, comprehensibility and formulation of the items and headings using a 10-point scale (0 = not good, 10 = very good). Furthermore, participants were asked their preference for one out of three answer options being 1) a 5-point Likert scale in words (checked for comprehensibility by the Dutch center of expertise in reducing health inequalities (PHAROS: www.pharos.nl)) or 2) a 5-point Likert-scale consisting of smiley icons or 3) a combination of both. Next, we asked for further suggestions. Participants were able to respond between 27-November-2023 and 19-December-2023. An overview of the member check questions is displayed in S4 Appendix. Member check.

### Researcher positionality/reflexivity

The research consortium consisted of researchers with diverse backgrounds. The core group (MS^1^, JK^2^, MB^3^, CR^4^, EB^5^, JD^6^) included six project members with expertise in psychology and statistics^1^, population health^2,3^, broad health concepts^1,2,5^, human biology and participatory design within oncology^4^, nutritional sciences and public health^2,3,4,5,^ qualitative research^2,3,6^ participatory research^3,6^, and shared decision-making^6^. All consortium members were familiar with the frameworks of Positive Health. Especially JD had expertise on the Capability Approach. While some researchers were advocates of Positive Health (EB^5^, JD^6^) as a way to capture a broader understanding of health, others (MS^1^, JK^2^, MB^3^ CR^4^) maintained a more critical stance, emphasizing conceptual clarity and empirical validity. This diversity of perspectives fostered reflexive dialogue throughout the research process.

Some consortium members (MdK, MvV) were affiliated with the Institute of Positive Health (iPH), and one member (EB) was a certified Positive Health trainer. These positions provided valuable insight and practical expertise regarding Positive Health, but could also introduce a tendency toward interpreting the questionnaire and results in alignment with the framework.

The FGDs were facilitated by different consortium members (CR, MB, EB, LNvV, TvZ, PMRC, JS). There were always two and sometimes three discussion leaders depending on who was most familiar with the target group.

Data were interpreted by two lead researchers (CR, MB) with complementary perspectives, and analytic decisions were discussed within the core group (MS, JK, CR, MB, EB, JD) to enhance reflexivity. Following analysis, the wider consortium and experts from the Dutch National Positive Health Network reviewed and provided feedback on the questionnaire. While this consultation offered valuable expertise, it could potentially introduce confirmatory bias, favoring interpretations aligned with the Positive Health framework. Combining independent analysis, core group discussions, and external feedback helped mitigate potential bias and strengthened the credibility and trustworthiness of the findings.

## Results

### Step 1: FGD results

Each FGD had a duration of ~120 minutes. Table 2 shows characteristics of the participants of the FGDs. There was a tendency towards more professionals with work experience between 5–10 years, and more women who participated. Mostly highly educated people participated among professionals, whereas among patients and citizens there were relatively more participants with a lower level of education.

**Table 2:** Overview and characteristics of the focus group discussion (FGD) participants.

	Total	Medical specialists	Regional policymakers & welfare	Citizens	Lower SEP citizens	National policymakers	Experts	Paramedics	Mixed	Patients*
**N**										
	76	10	8	8	8	14	8	6	6	8
**Age (median; IQR)**										
	46.5 (35.3-58.8)^1^	43.0 (39.0-57.0)^2^	36.5 (26.8-40.3)	78.0 (70.0-83.0)^3^	47.0 (42.0-67.0)	44.0 (30.3-61.3)	48.5 (27.0-55.3)	40.0 (34.0-49.0)	53.0 (27.0-55.0)	60.0 (44.3-77.8)
Educational level**										
lower and intermediate	19	3	1	2	6	0	0	0	0	7
higher	56	7	7	6	1	14	8	6	6	1
do not want to disclose	1	0	0	0	1	0	0	0	0	0
**Gender**										
male	22	0	2	6	1	3	2	2	1	5
female	53	10	6	2	6	11	6	4	5	3
do not want to disclose	1	0	0	0	1	0	0	0	0	0
**Work experience**										
<2 years	4	0	2	0	1	0	0	0	0	1
2-5 years	52	8	2	6	5	9	5	6	4	7
5-10 years	12	2	1	1	0	3	3	0	2	0
>10 years	8	0	3	1	2	2	0	0	0	0

*There were two FGDs organized for patients. These are grouped together because one FGD included only one patient and was held as an interview.**Lower education level was categorized as having primary school or lower vocational education; Intermediate education level was categorized as having secondary vocational education; Higher educational level was categorized as having higher vocational education, university degree, postdoctoral degree.^1^4 missing values; ^2^3 missing values; ^3^1 missing value.

The qualitative analysis yielded input on several overarching themes. These themes included “Formulation of items”, “Overlap between items”, “Redundant items”, “Importance of items”, “New suggestions”, and “Length/duration of the questionnaire”. Table 3 shows an overview of the themes that emerged from the qualitative analysis.

**Table 3:** Themes that emerged from the focus group discussions (FGDs) about the original CPHQ items.

Theme	Explanation/examples
**1.Formulation of items**	Items in general, particularly related to health literacy, could be formulated shorter and easier to make them more understandable for people with limited literacy. For example the wording of some of the items that used “I am able to...” could be rephrased using alternative wordings such as “I can”. -Examples or normative words such as “adequate” should be avoided. -According to healthcare professionals items about feeling disadvantaged should be phrased positively. However, citizens and patients did not express this need and reported that they felt these items were important. Uncertainty about how to interpret the word “environment” in items. It could indicate their street, neighborhood, municipality but also their family and friends. This ambiguity made it difficult for the participants to answer these questions. They felt that the question should not be open for interpretation.
**2.Overlap between items**	Overlap was reported between; - “It is hard for me to move on when something bad happens”, “I find it difficult to cope with stressful events”, and “It does not take me long to recover from a stressful event”. - “I can afford to live a healthy life” and “I can afford healthy food and exercise”. - items related to health literacy and access to health information: “I can communicate with healthcare workers and understand the explanation about my illness or treatment”, “It is clear to me where I need to go for medical help” and “When I look up or receive information about a subject, it is explained in a way that I can understand”.
**3.Redundant items**	- Political items should be avoided in a questionnaire to measure broad health.
**4.Importance of items**	Most participants expressed that the items “I feel healthy”, “I feel fit”, and items related to relaxation were important items to measure health.
**5.New suggestions**	According to professionals an item related to a “sense of purpose” was missing. They suggested items that concerned ideals/goals in life or “I perceive my life as meaningful”.^1^However, citizens and patients did not express the need for such an item.
**6.Duration/length of the questionnaire**	Participants expressed that 32 items were too much.

^1^These suggestions also received seven golden tickets from participants.

After the quantitative analysis, the overall outcome was that eight items were retained, two items were omitted, 22 items were adjusted, of which seven items were merged with other items. This led to a total of 23 items. To illustrate, see S5 Appendix. Worked out example shows the decision flow of item 7. See Table 4 for an overview of the quantitative results. In total, 76 golden tickets could be given, of which 22 were given to “I feel healthy” and the others were distributed across diverse items without a clear preference. Furthermore, six participants did not use their golden ticket and 13 golden tickets were given to items from the ‘hand book’ or their own self-defined items.

**Table 4:** Quantitative outcomes and adjustments of items based on the focus group discussions (FGDs).

Original CPHQ item	Quantitative score	Consensus	Golden tickets (n)^1^ guiding decisions (Y)	Qualitative theme guiding decisions^2^	Decisions^3^	Revised CPHQ item
1: I am able to unwind	Retain	No	1	2	Merged with item 18	/
2: I am capable of carrying out tasks and activities adequately	Omit	Yes	0	1,6	Merged with item 3, removed normative phrasing	/
3: I am able to participate in activities that I value in my daily life (work, study, etc.)	Retain	No	1	1	Formulated easier and shorter, excluded examples	I can do what I think I important
4: I can work/volunteer	Adjust	No	0	1,6	Merged with item 3	/
5: I feel healthy	Retain	Yes	22	4	No changes	I feel healthy
6: I feel in good health	Adjust	No	0	1,4	Differentiated between physical fitness and mental wellbeing	I feel fit I feel good about myself
7: I can move easily, such as climbing stairs, walking, or cycling	Adjust	No	0	1	Excluded examples	I can move
8: I can find people with whom I can have a good time	Adjust	No	1	1	Formulated easier and shorter	I have people I can do fun things with
9: I feel that people support me when needed	Retain	Yes	0	1	Formulated easier and shorter	I feel that people support me
10: I feel that I ‘fit in’ in my environment	Adjust	Yes	1	1	Formulated easier and shorter, removed ‘environment‘because of ambiguity	I feel that I belong
11: I can afford to live a healthy lifestyle	Adjust	No	1	2	Merged with item 26	/
12: I feel happy	Retain	Yes	0	/	No changes	I feel happy
13: I am able to enjoy life	Retain	Yes	12	1	Formulated easier and shorter, removed ‘my life’ because of ambiguity	I can enjoy
14: I am able to be grateful for what life has to offer	Adjust	No	4	1	Formulated easier and shorter	I feel grateful for my life
15: When something bad happens, it is difficult for me to move on	Adjust	No	6, Y	1	Formulated easier and shorter	I can cope with setbacks
16: I have a hard time getting through stressful situations	Adjust	No	0	2	Merged with item 15	/
17: I don’t need much time to recover from a stressful event	Adjust	Yes	0	2	Merged with item 15	/
18: I am able to relax, when necessary	Retain	No	0	1	Formulated easier and shorter removed ‘when necessary’ because of ambiguity	I can relax
19: I have enough peace of mind	Adjust	No	0	1	Formulated easier and shorter	I feel calm
20: I feel safe in the neighborhood where I now live	Retain	Yes	3	1	Formulated easier and shorter, removed ‘neighborhood; (in Dutch omgeving (area)) because of ambiguity	I feel save where I live
21: My home environment is safe and provides numerous opportunities to engage in daily life	Adjust	No	1	1	Formulated easier and shorter	I can be active in my environment
22: I feel connected to the environment where I now live	Adjust	No	0	6	Merged with item 10	/
23: I feel disadvantaged because of my religion or spiritual beliefs	Adjust	No	0	1	Formulated easier and shorter, excluded examples	I feel accepted
24: I feel disadvantaged because of my (cultural) background	Adjust	No	2	1	Formulated easier and shorter	I feel excluded
25: I feel disadvantaged or excluded based on my sexuality and/or gender	Adjust	No	0	6	Merged with item 23, 24	
26: I can afford to eat healthily and participate in physical activities	Adjust	Yes	1	1	Formulated easier and shorter, excluded examples	I have money to life a healthy life
27: I have enough money to do things that are important to me	Adjust	No	0	1	Removed normative phrasing	I have money to do the things tI find important
28: Politics makes me feel represented	Omit	Yes	1	3	Omitted	/
29: I feel confident in the way that politicians handle issues that are important to me	Adjust	Yes	0	1	Formulated easier and shorter	I have confidence in society
30: I can communicate with healthcare professionals and understand their explanations of my illness or treatment	Adjust	No	0	2	Omitted	/
31: I know where to go for medical assistance	Adjust	No	1	1	Formulated easier and shorter	I can find help for my health
32: When I look up or receive information about a subject, it is explained in a way that I can understand	Adjust	No	0	1	Formulated easier and shorter	I can understand information about my health

^1^13 golden tickets were given to items from the handbook or items added by participants. ^2^Themes refer to the themes mentioned in table 3. ^3^Decisions are made by the core group. Refinement of the items after expert validation (step 2) is included in the final item. This questionnaire is developed in the Netherlands and in the Dutch language. All final items were translated to English by a native speaker from a translating agency. Back-to-back translation did not take place. Final items are available in Dutch and English in S6 Appendix. 28-item CPHQ.

### Step 2: Expert validation and member check results

#### Expert validation results.

The expert validation led to some refinement in formulation of items, e.g., all phrased in a similar way. Based upon the expert validation the core group made a couple of final adjustments (See Table 4). In line with the Capability construct and the preferred short items, “I am able to enjoy my life” and “I manage to relax when necessary” were adjusted to “I can enjoy” and “I can relax”, respectively. Indicative words such as “my life” and “when necessary” were left out since enjoyment and relaxation are not dependent on those indications and can be filled in by personal circumstances. Similarly, for the sentences “I feel that people support me when I need it“ and “I feel safe in the area where I live now”, the words “when I need it”, “area” and “now” were omitted. For the question “I am able to do activities that I find important in daily life (work, study, etc.)”, the core group decided to omit the examples and change the word activities to a more general verb “can”, formulating the new question as: “I can do what I think is important”. For the item “I feel fit”, there was a lot of debate about whether this referred to physical fitness or mental fitness. Therefore, “I feel fit” was left in the questionnaire referring to physical fitness and the additional item “I feel good” was added, referring to mental wellbeing.

Furthermore, based on expert validation and discussion within the core group (with input of the FGDs), five items that were previously omitted based on the COMET decision rules, were adapted and added, doing justice to the broad concept of health [25]. See Table 5 for an overview of the additional five items and reasons for adding these to the revised CPHQ. This increased the list of 23 items to 28 items.

**Table 5:** Additional items added after expert validation and core group discussion.

Item	Reason for adding the item
1: I feel that people support me	Professionals and experts had doubts about whether feeling support is the only aspect of support. Therefore having people who support was seen as important to additionally add.
2: I have goals and ideals I want to achieve	Professionals and experts expressed that meaningfulness/sense of belonging was important to add.
3: I can work/volunteer or provide informal care	Professionals and experts expressed that work/volunteering or informal caregiving is a way of participating in society and may be important to add.
4: I can express my boundaries	Professionals and experts expressed that setting boundaries may be important with respect to resilience and thus important to add.
5: I feel my life is meaningful	Professionals and experts expressed that meaningfulness/sense of belonging was important to add.

This questionnaire is developed in the Netherlands and in the Dutch language. All final items were translated to English by a native speaker from a translating agency. Back-translation did not take place. Final items are available in Dutch and English in S6 Appendix. 28-item CPHQ.

Subsequently, these 28 items were categorized into the components of the Capability Approach (Table 6). This was done by the core team. All core team members coded the items independently as either a resource, conversion factor capability or functioning [5,7,27]. After independently coding each item, the core team discussed until consensus was reached about the appropriate categorization of each item.

**Table 6:** Items of the revised CPHQ categorized according to the Capability Approach.

**Resources**	I have people I can do fun things with
	I have money to live a healthy life
	I have money to do the things I find important
	I have people who support me
**Conversion factors**	I feel excluded
	I feel accepted
	I feel that people support me
	I feel that I belong
	I feel grateful for my life
	I feel safe where I live
	I have confidence in society
	I have goals and ideals I want to achieve
**Capabilities**	I can relax
	I can do what I think is important
	I can move
	I can enjoy
	I can cope with setbacks
	I can be active in my environment
	I can find help for my health
	I can understand information about my health
	I can work/volunteer or provide informal care
	I can express my boundaries
**Functionings**	I feel healthy
	I feel fit
	I feel good about myself
	I feel calm
	I feel happy
	I feel my life is meaningful

This questionnaire is developed in the Netherlands and in the Dutch language. All final items were translated to English by a native speaker from a translating agency. Back-translation did not take place. Final items are available in Dutch and English in S6 Appendix. 28-item CPHQ.

Five items were categorized as resources and phrased accordingly (e.g., “I have people […])”. Seven questions were categorized as conversion factors, referring to factors that influence how people can convert resources into capabilities. These were phrased for example as “I feel people support me”. Ten items were categorized as capabilities and phrased using “I can…”. Six items were categorized as functionings, related to their physical or mental state, and phrased for example as “I feel happy”. Categorization according to the Capability Approach was done because experts felt that the CPHQ questionnaire should be based on theory.

As the questionnaire was developed in the Netherlands, the original items are in Dutch. These can be found in S6 Appendix. 28-item CPHQ.

### Member check results

The member check was sent to 55 of the 76 participants of the FGD participants who provided their email address and agreed to be contacted for additional information. The member check was conducted in the period between 27 November 2023 and 19 December 2023. In total, we received 18 replies (33%). Readability, comprehensibility and headings all scored good (see Table 7).

**Table 7:** Results of the member check.

Score of the questionnaire/items^1^	Mean±SD
Readability	8.0 ± 1.6
Comprehensibility	8.2 ± 1.1
Subheadings	7.5 ± 1.7
**Preference of answer options** ^**2**^	**Number (%)**
Likert-scale in smileys	8 (44%)
Likert-scale in text	5 (28%)
Likert-scale with a combination of smileys and text	2 (11%)

^1^Scored on a scale between 0–10 (i.e., not good to very good) ^2^3 participants gave no answer on preferred answer option.

There was no consensus based on what kind of answer options would be best suitable by the participants (see S4 Appendix. Member check.). Given the various preferences, the core group decided for the most inclusive option being textual answers on a five-point Likert-scale accompanied with smileys on a five-point Likert-scale.

Suggestions of participants concerned the answer format (5/18), section headings (3/18), formulation of items (7/18), categorization of items (3/18), target population (3/18), satisfaction (2/18), and applicability for secondary care (1/18) (see Table 8). Feedback on the answer format and categorization of items to subheadings will be used for future refinement of the CPHQ. With regard to the grouping and the applicability, no adjustments were made since the capability essentials were included in the grouping and the instrument is also designed for other domains than secondary care. With regard to the formulation, two participants questioned whether the opposite item “I feel excluded” would need to be stated in a positive formulation. We decided, however, to leave this item in the questionnaire because vulnerable populations such as lower SEP citizens did not recognize themselves in their positively formulated counterparts. This was also previously observed by Doornenbal et al [15]. Other remarks concerned the interpretation of words such as ‘grateful’ and ‘calm’, which is preference-sensitive and to further define certain items. We decided not to do this because at an earlier stage we decided to omit examples from the items based on the feedback we obtained in the FGDs.

**Table 8:** Feedback received from participants of the member check.

Type of feedback	Examples	Frequency of feedback
Answer format	Preference for using smileys, Preference for textual scales and Likert scales	5/18
Section headings	Clarity and conceptual fit of headings	3/18
Formulation of items	Comments on negative phrasing (I feel excluded) or ambiguity (grateful or calm)	7/18
Categorization	Suitability of items underneath a subheading	3/18
Target population	Understandability of items (e.g., understandability of ‘perceived’ health for people with limited literacy) and applicability in secondary care	3/18
Satisfaction with items	No comments, reply to state satisfaction	2/18
Applicability for secondary care	Doubts about suitability for secondary care	1/18

## Discussion

This study aimed to develop a revised version of the 32-CPHQ to measure broad health. The resulting 28-item version is intended for use across multiple domains, including the healthcare and welfare policy domain, research and with specific attention to vulnerable populations (e.g., individuals with a lower SEP). The development process involved stakeholders from all aforementioned domains and builds on Positive Health and the Capability Approach. This ensures that the revised- 28 item CPHQ is theoretically grounded, reflects stakeholder and end-user perspectives and ensures content validity [28].

### Key findings

#### Commonalities.

One key point across all FGDs was the consensus to avoid examples and normative phrasing in questionnaire items. This allows for more subjective interpretation by the respondents. For instance, the item “I am able to do activities that I find important in daily life (work, study, etc.)” originally included examples, but participants emphasized that what is considered important varies across individuals and contexts [6,7]. Allowing room for personal interpretation aligns with the aim to measure health in a context-sensitive way. However, it may result in their answers reflecting different underlying constructs, meaning that the item can no longer reliably measure a single, clearly defined concept. If so, this reduces the internal consistency. Further research is needed to examine this.

Another commonality was that the item “I feel healthy” was perceived as the most important item by participants. Other frequently emphasized items included “I feel in good health,” “I feel good,” and “I feel happy”. Interestingly, these items reflect functionings in terms of the Capability Approach: they describe actual states of being and doing [5]. Some of these items, such as “I feel healthy” or “I feel happy” are also used as global or standalone indicators in instruments that measure health, quality of life or wellbeing. For instance, the EQ-5D-5L includes a visual analogue scale (VAS) where respondents rate how healthy they feel, and single-item measures of health and happiness are also used in population health surveys [23,29]. This suggests that such functionings may not only serve as outcomes but also as overarching, integrative indicators of health. There could be overlap between these functionings and global health, wellbeing or quality of life indicators when interpreted as summary indicators rather than a dimension among others. Future research, for example examining correlations with these global indicators is relevant to get insight into potential overlap and usability as standalone items. According to the Capability Approach, what matters is not only the realization of these experienced states but the freedom and opportunity (i.e., capabilities) to achieve them. This implies that functionings may not be sufficient as a sole basis to assess broad health, but rather that they occupy a central yet incomplete part of broad health. So far it is unclear whether functionings represent a distinct dimension of broad health or rather that they represent a higher order factor. Psychometric analyses such as factor analysis and hierarchical models are therefore needed to clarify the structure and ensure appropriate interpretation. From a Capability Approach perspective, an instrument that focuses solely on functionings may miss whether people actually had the opportunity to achieve these states, while instruments that only assess capabilities might miss how people actually perceive their health. Therefore, we suggest that to assess broad health, a measurement instrument should include both elements.

Participants also discussed the item “I feel represented by politics”, and agreed that it should be removed, albeit for different reasons. Medical professionals questioned its relevance, as it was perceived to lie beyond their professional influence. Patients and citizens felt that it was unrelated to their own health. In the qualitative feedback, the item was described as abstract and difficult to interpret. Interestingly, a related item ”I have confidence in society” remained in the refined CPHQ. Previous research shows a link between poor health with lower political trust [30]. Mattilla (2020) suggests that one reason for this lower trust might be a disconnect between what citizens expect from the government and institutions and how they actually perceive its outcomes, particularly regarding how fairly they feel treated [30]. From a Capability Approach perspective, political representation can act as a conversion factor that shapes people’s ability to achieve valued states [6]. Moreover, in the original CPHQ, items on political representation formed a distinct factor in the factor analysis [15]. These differing perspectives reflect the complexity of determining which contextual and societal factors should be included in a measure of broad health. We based our inclusion of the item “confidence in society” and omission of “political representation” on our a priori set decision rules. We acknowledge that the omission of a politically tinged item, may limit the breadth of the construct measured. While the retained item “I have confidence in society” may capture social trust, conversion factors concerning politics are less directly assessed. Our decision possibly reflects a trade-off between construct completeness and interpretability as participants found the item about political representation difficult to understand and abstract. We therefore recommend that future validation studies further explore whether and how aspects like political representation and trust in institutions contribute to people’s perceived capabilities for broad health. Rather than including these items by default, their relevance may need to be assessed contextually, depending on the population or on the purpose of the measurement.

### Differences

There were also some key differences between the stakeholder groups. End-user participants (i.e., (lower SEP) citizens and patients) preferred the item “I feel excluded” to be phrased negatively as they did not recognize themselves in its positive form. This reflects findings from the original CPHQ development [15]. Professionals, researchers and experts, however, favored positive phrasing. This divergence may reflect differences in how broad health is conceptualized across social strata. Several studies suggest that individuals with lower education levels are more likely to define health in negative terms [31–34]. To illustrate, Peersman et al. found that lower-educated respondents referenced the presence of health problems, while higher-educated individuals emphasized the absence of such problems [32]. The differences may stem from both cultural framing and real differences in health experiences across socioeconomic groups [34]. To ensure that health measurement tools are inclusive and sensitive to different lived realities, it may be useful to examine how individuals from different SEP strata interpret specific items, particularly those related to exclusion or disadvantage. While we already included citizens and low SEP citizens, future development and implementation efforts may benefit from continued attention to subgroup differences in interpretation of item phrasing.

Another difference concerned the inclusion of the concept of meaningfulness. Where professionals, researchers and experts emphasized its importance, citizens and patients did not mention meaningfulness. This may be due to the abstract nature of the concept, which may not surface spontaneously in discussions. Nonetheless, meaningfulness has been emphasized in the literature on Positive Health, even by patients [2,3]. Also in earlier research, in the concept of salutogenesis, which is about the origins of health and focuses on factors that support health, developed by Antonovsky, meaningfulness is seen as an important asset for health [35]. Following expert consultation and discussion within the core group, an item on meaningfulness was added to the refined questionnaire. Future psychometric analysis will determine whether it should be retained.

### Strengths and limitations

This study has several strengths. First, the questionnaire was developed through co-creation with diverse stakeholders from healthcare, welfare, and policy domains and including patients and (lower SEP) citizens. This user-centered approach increases the likelihood of acceptance and applicability in various settings. Second, the inclusion of citizens with a lower SEP and participants from different regions of the Netherlands improves the relevance and representativeness of the instrument. Third, the iterative development process, including expert validation and a member check, enhanced the credibility and robustness of the 28-item CPHQ. Fourth, the questionnaire is theoretically grounded not only in Positive Health but also in the Capability Approach, which adds more attention to contextual influences on health. Fifth and finally, recurring themes across FGDs suggest that thematic saturation has been reached.

Nevertheless, some limitations should be acknowledged. There may be some recruitment bias towards individuals who are already more positive towards the concept of broad health. Despite efforts to recruit a diverse group of stakeholders from different professional backgrounds and geographical regions within the Netherlands, it cannot be ruled out that participants were positively inclined toward Positive Health as one of the concepts of broad health. To reduce this risk, during recruitment we explicitly invited both proponents and critics and FGD facilitators actively stimulated critical discussion. Nevertheless, this potential bias should be taken into account when interpreting the findings. The omission of the item “I feel represented by politics” may reduce coverage of a conversion factor that theoretically could influence health. This reflects a trade-off between interpretability and construct completeness as the item was perceived as difficult and abstract. Future validation studies could explore whether including this item adds meaningful information, depending on the context, target population or the purpose of the measurement. Additionally, consensus was not always reached for specific items. Some decisions with respect to the wording of items were therefore compromises and may not be ideal formulations for all stakeholders. However, a member check was performed for contingency. Unfortunately, the response rate for the member check was low which may cause non-response bias. We cannot examine whether these participants were representative of all FGD participants. It could be that the response of FGD participant groups was unequal, and that results reflect predominantly the perspectives of more accessible and articulate participants. In particular, (lower SEP) citizens and patients may have been less likely to participate in the member check compared with professionals. As a result, some perspectives might be underrepresented in the final feedback on new CPHQ items. Finally, while the CPHQ is meant to be both suitable to assess broad health using population monitoring and program evaluation. Compared to population monitoring where understandability may be more important, good sensitivity to short-term changes is needed for program evaluation. Sensitivity to detect shorter and longer term changes is not yet known and remains to be confirmed.

### Implications and future research

The revised CPHQ offers a promising tool to measure broad health. It could provide valuable insights into the effectiveness of health and welfare interventions or (policy) programs that are currently assessed from a more narrow perspective. Furthermore, such a measurement instrument may be used for population monitoring. In particular, the functioning items are of added value on top of patient report outcomes or biomedical indicators as it reflects real opportunities and lived health instead of biomedical status and limitations. However, it is a challenge to measure broad health with a compact questionnaire. In further research, we will investigate if the revised CPHQ is sufficiently sensitive to pick up small changes in health status.

Next steps in the validation of the revised CPHQ include item reduction analysis (e.g., by factor analysis) to assess the most important items and to potentially shorten the questionnaire. Questionnaire length can have a serious impact in how the questionnaire is filled out and on response rates [36]. Discriminant validity should be assessed by examining scores on the revised CPHQ across different subpopulations. Examining correlations with other health questionnaires (e.g., EQ-5D-5L) and analyzing changes in scores over time will further support its validity. Furthermore, future research should focus on its responsiveness in diverse populations and settings. This is in line with a recent rapid review on the overall concept of Positive Health which emphasizes the need for more clarity regarding suitable settings and target groups for the implementation of Positive Health, with special attention paid to the inclusion of vulnerable populations [37]. Once the revised CPHQ has been evaluated and proven useful in several subpopulations and settings, it would be interesting to investigate how the revised CPHQ relates to other questionnaires measuring broad concepts of health, such as the 17-item Positive Health questionnaire and whether it is possible to adapt it to other populations such as youth.

## Conclusions

In conclusion, the revised CPHQ was developed with input from a wide range of stakeholders, including patients and (lower SEP) citizens. This led to a 28-item questionnaire to measure broad health. Future research, through cross-sectional or longitudinal research in varying populations and settings, has to ensure its validity.

## Supporting information

S1 Appendix 1. FGD posters. (DOCX)

S2 Appendix 2.Hand Book. (DOCX)

S3 Appendix 3. Example prompts. (DOCX)

S4 Appendix 4. Member check. (DOCX)

S5 Appendix 5. Worked out example. (DOCX)

S6 Appendix 6. 28 item CPHQ. (DOCX)
