# Supplementary material for: Stakeholder and end-user perspectives to refine the Context-sensitive Positive Health Questionnaire (CPHQ) to measure broad health
Source: PLoS One. 2026 Aug 24;21(8):e0330014. doi: 10.1371/journal.pone.0330014 (PMC13502646; doi:10.1371/journal.pone.0330014)
Supplement: Supplementary file 1 [file pone.0330014.s001.docx]

**Appendix 1. FGD posters.**

 Example of a poster used during the focus groups discussions (FGD). On top is the theme “perceived health”. Underneath from left to right: “Questions”, “Judgement” and “Remarks”. Each participant received green (keep), yellow (adjust) and orange/red (omit) memos with a participant ID on it. In addition, they received memos to add comments.

Categorization of items across the four A0 posters is displayed in table 1. Posters were discussed in the focus group discussions at random. In table 1 the Dutch items are displayed next to the English free text translation (unofficial).

| **Table 1.** Items distributed across posters for focus group discussions in Dutch and Freely translated to English. | | |
| --- | --- | --- |
|  | **Dutch** | **English** |
|  | **Poster 1** |  |
| **Item/title** | **Beleefde gezondheid** | **Perceived health** |
| 1 | Ik ben in staat om te ontspannen | I am able to unwind |
| 2 | Ik ben in staat om taken en activiteiten adequaat uit te voeren | I am capable of carrying out tasks and activities adequately |
| 3 | Ik ben in staat om in het dagelijks leven activiteiten te doen die ik belangrijk vindt (werk, studie, etc) | am able to participate in activities that I value in my daily life (work, study, etc.) |
| 4 | Ik kan werken/vrijwilligerswerk doen | I can work/volunteer |
| 5 | Ik voel mij gezond | I feel healthy |
| 6 | Ik voel mij fit | I feel in good health |
| 7 | Ik kan makkelijk bewegen, bijvoorbeeld traplopen, wandelen of fietsen | I can move easily, such as climbing stairs, walking, or cycling |
| 8 | Ik kan mensen vinden met wie ik leuke dingen kan doen | I can find people with whom I can have a good time |
| 9 | Ik voel dat mensen mij steunen als dat nodig is | I feel that people support me when needed |
|  | **Poster 2** |  |
| **Item/title** | **Beleefde gezondheid** | **Perceived health** |
| 10 | Ik voel dat ik ‘erbij hoor’ in mijn omgeving | I feel that I ‘fit in’ my environment |
| 11 | Ik kan het betalen om gezond te leven | I can afford to live a healthy lifestyle |
| 12 | Ik voel mij gelukkig | I feel happy |
| 13 | Ik ben in staat om te genieten van mijn leven | I am able to enjoy life |
| 14 | Ik ben in staat om dankbaar te zijn voor wat het leven mij biedt | I am able to be grateful for what life has to offer |
| 15 | Het is moeilijk voor mij om verder te gaan als er iets vervelends gebeurt | When something bad happens, it is difficult for me to move on |
| 16 | Ik vind het moeilijk om stressvolle gebeurtenissen te doorstaan | I have a hard time getting through stressful situations |
| 17 | Ik heb niet veel tijd nodig om van een stressvolle gebeurtenis te herstellen | I don’t need much time to recover from a stressful event |
|  | **Poster 3** |  |
| **Item/title** | **Persoonlijke context** | **Personal context** |
| 18 | Het lukt mij te ontspannen wanneer dat nodig is | I am able to relax when necessary |
| 19 | Ik heb voldoende rust in mijn hoofd | I have enough peace of mind |
| 20 | Ik voel mij veilig in de omgeving waar ik nu woon | I feel safe in the neighbourhood where I now live |
| 21 | Mijn omgeving is veilig en geeft genoeg mogelijkheden om actief te kunnen zijn | My home environment is safe and provides numerous opportunities to engage in daily life |
| 22 | Ik voel mij verbonden met de omgeving waarin ik nu woon | I feel connected to the environment where I now live |
| 23 | Ik voel mij benadeeld vanwege mijn religie of spirituele levensovertuiging | I feel disadvantaged because of my religion or spiritual beliefs |
| 24 | Ik voel mij benadeeld vanwege mijn (culturele) achtergrond* | I feel disadvantaged because of my (cultural) background |
| 25 | Ik voel mij benadeeld of buitengesloten op basis van mijn seksualiteit en/of gender | I feel disadvantaged or excluded based on my sexuality and/or gender |
|  | **Poster 4** |  |
| **Item/title** | **Persoonlijke context** | **Personal context** |
| 26 | Ik kan gezonde voeding en beweegactiviteiten betalen | I can afford to eat healthily and participate in physical activities |
| 27 | Ik heb genoeg geld om dingen te doen die belangrijk voor mij zijn | I have enough money to do things that are important to me |
| 28 | Ik voel mij vertegenwoordigd vanuit de politiek | Politics makes me feel represented |
| 29 | Ik heb vertrouwen in hoe de politiek omgaat met dingen die voor mij belangrijk zijn | I feel confident in the way that politicians handle issues that are important to me |
| 30 | Ik kan communiceren met zorgmedewerkers en begrijp de uitleg over mijn ziekte of behandeling | I can communicate with healthcare professionals and understand their explanations of my illness or treatment |
| 31 | Het is voor mij duidelijk waar ik moet zijn voor medische hulp | I know where to go for medical assistance |
| 32 | Als ik informatie opzoek of ontvang over een onderwerp, wordt dit op een voor mij begrijpelijke manier uitgelegd | When I look up or receive information about a subject, it is explained in a way that I can understand |
