# Supplementary material for: Stakeholder and end-user perspectives to refine the Context-sensitive Positive Health Questionnaire (CPHQ) to measure broad health
Source: PLoS One. 2026 Aug 24;21(8):e0330014. doi: 10.1371/journal.pone.0330014 (PMC13502646; doi:10.1371/journal.pone.0330014)
Supplement: Supplementary file 2 [file pone.0330014.s002.docx]

**Appendix 2. Hand book.**

**1. Overview of questionnaires in the hand book**
Some questionnaires are only available in Dutch. The questionnaires that were included in the hand book were:
-Original CPHQ(1)
-My Positive Health-42 (MPH-42)(2)
-My Quality of Life Questionnaire(3)
-The subjective wellbeing-5 dimensions questionnaire (SWB-5D)(4)
-ICEpop CAPability measure for Adults (ICECAP-A)(5)
-Vita-16(6)
-Brief Resilience Scale (BRS)(7)
-Personal Wellbeing Index- Adult (PWI-A)(8)
-EuroQol 5-dimensions (EQ5D-5L)(9, 10)
-Multicultural Quality of Life Index (MQLI)(11)

**2. Items per questionnaire**
All items in the handbook are in Dutch as the focus group discussions (FGDs) took place in the Netherlands and were in Dutch. For this appendix the items are reported in English, if authors provided an (un)official English translation. The Vita-16 is not provided in English by the authors and is therefore provided in Dutch. Questionnaires are provided in table 1-10.

**Table 1.** The original CPHQ.

I am able to unwind

I am capable of carrying out tasks and activities adequately

I am able to participate in activities that I value in my daily life (work, study, etc.)

I can work/volunteer

I feel healthy

I feel in good health

I can move easily, such as climbing stairs, walking, or cycling

I can find people with whom I can have a good time

I feel that people support me when needed

I feel that I ‘fit in’ in my environment

I can afford to live a healthy lifestyle

I feel happy

I am able to enjoy life

I am able to be grateful for what life has to offer

When something bad happens, it is difficult for me to move on

I have a hard time getting through stressful situations

I don’t need much time to recover from a stressful event

I am able to relax when necessary

I have enough peace of mind

I feel safe in the neighborhood where I now live

My home environment is safe and provides numerous opportunities to engage in daily life

I feel connected to the environment where I now live

I feel disadvantaged because of my religion or spiritual beliefs

I feel disadvantaged because of my (cultural) background

I feel disadvantaged or excluded based on my sexuality and/or gender.

I can afford to eat healthily and participate in physical activities

I have enough money to do things that are important to me

Politics makes me feel represented

I feel confident in the way that politicians handle issues that are important to me

I can communicate with healthcare professionals and understand their explanations of my illness or treatment

I know where to go for medical assistance

When I look up or receive information about a subject, it is explained in a way that I can understand

**Table 2.** The MPH-42.

Feeling healthy

Feeling fit

Having complaints or pain

Sleeping pattern

Eating pattern

Physical condition

Exercise

Being able to remember things

Being able to concentrate

Being able to communicate

Being cheerful

Accepting yourself

Being able to handle changes

Having control

Having a meaningful life

Being high-spirited

Wanting to achieve ideals

Feeling confident about own future

Accepting life

Being grateful

Continue learning

Enjoyment

Being happy

Feeling good

Feeling well-balanced

Feeling safe

Living conditions

Having enough money

Social contacts

Being taken seriously

Doing fun things together

Having the support of others

Belonging

Doing meaningful things

Being interested in society

Looking after yourself

Knowing your limitations

Knowledge of health

Managing time

Managing money

Being able to work

Asking for help

**Table 3.** The My quality of Life questionnaire.

I feel happy

I enjoy my life

I am happy with my life as it is

I think my life is meaningful and purposeful

I live in a way that fits me

I make my own choices

I know what I want in life

I feel strong due to my life conceptions and beliefs

I feel good about the responsibilities I have

I have a good balance between activity and relaxation

I spent time on things I think are important

I have a good balance between being alone and among people

I have a warm and trusted relations with other people

I feel taken serious by other people

I feel accepted in my neighborhood/environment

I feel safe in my neighborhood/environment

I can go to others if I need help

I can mean something for other people

I feel useful

I dare to ask when I need help

I feel physically healthy

I feel mentally healthy

I feel filt enough to do what I want

I can deal with change and setbacks

Go where I want in my residence, goes

Go when I want in my residence, goes

Visiting neighbors, friends and acquaintances when I want goes

Undertaking trips as I want to goes

Going to work and/or locations like I want goes

The possibility for an equivalent conversation with people I love is

The social contact with people I love is

The respect I receive from people I love is

The social contact with people I know less is

The respect I receive from people I know less is

The possibility to give and receive love and affection is

The possibility to see people as often as I want

The possibility to help or support people that need me is

Washing, dressing and grooming the way I want goes

Washing, dressing and grooming when I want goes

Going to bed and getting out of bed when I want goes

Going to the toilet when I need and want to goes

Eating and drinking when I want goes

Eating and drinking what I want goes

Eating and drinking where I want

The possibility to do paid or unpaid work is

The possibility to do work that fits me is

Contact with my colleagues is

The possibility to improve or keep my position is

The possibility to change position or employer is

The possibility to follow education of my choice is

The possibility to pay what I need with my income is

Keeping track of my money is

Keeping track of my expenses is

The possibility to spent my money as I want is

The possibility to spent my time as I want is

The possibility to spent my free time as I want is

**Table 4.** The SWB-5D.

My health state restricts my physical activities

During the past week, I felt happy

During the past week, I felt lonely

I live my life my own way

For me, life is a continuous process of learning, change and growth

**Table 5.** The ICECAP-A.

Feeling settled and secure

Love, friendship and support

Being independent

Achievement and progress

Enjoyment and pleasure

**Table 6.** The Vita-16.

Ik heb genoeg energie om al mijn dagelijkse activiteiten te kunnen volbrengen

Ik bruis van de energie

Mijn batterij is 100% opgeladen aan het begin van de dag

Na het avondeten zit ik nog vol energie

Ik verheug mij op elke nieuwe dag

Ik maak plannen voor de toekomst

Als ik een doel heb, maak ik direct plannen om dit doel te bereiken

Het behalen van mijn doelen maakt mij gelukkig

Ik krijg energie van het maken van toekomstplannen

Ik vind het heel erg belangrijk om mijn doelen werkelijkheid te laten worden

Ik ga meteen aan de slag met nieuwe uitdagingen

Ik kan heel goed omgaan met tegenslagen

Ik kan heel goed oplossingen vinden in moeilijke situaties

Na een moeilijke periode ben ik snel weer de oude

Door mijn ervaring voel ik mij sterker in moeilijke tijden

Elke ervaring in het leven maakt mij sterker

**Table 7.** The BRS.

I tend to bounce back quickly after hard times

I have a hard time making it through stressful events

It does not take me long to recover from a stressful event

It is hard for me to snap back when something bad happens

I usually come through difficult times with little trouble

I tend to take a long time to get over set-backs in my life

**Table 8.** The PWI-A.

How satisfied are you with your life as a whole?

How satisfied are you with your standard of living?

How satisfied are you with your health?

How satisfied are you with what you are achieving in life?

How satisfied are you with your personal relationships?

How satisfied are you with how safe you feel?

How satisfied are you with feeling part of your community?

How satisfied are you, with your future security?

How satisfied are you with your spirituality or religion?

**Table 9.** The EQ-5D-5L.

Mobility

Self-care

Usual activities (e.g. work, study, housework, family or leisure activities)

Pain / Discomfort

Anxiety / Depression

**Table 10.** The MQLI.

Physical well-being (feeling energetic, free of pain and physical problems)

Psychological / Emotional well-being (feeling good, comfortable with yourself)

Self-care and independent functioning (carrying out daily living tasks; making own decisions)

Occupational functioning (able to carry out work school, and homemaking duties)

Interpersonal functioning 9able to respond and relate well to family, friends and groups)

Social-emotional support (availability of people you can trust and who can offer help and emotional support)

Community and services support (pleasant and safe neighbourhood, access to financial, informational and other resources)

Personal fulfillment (experiencing a sense of balance, dignity, and solidarity; enjoying sexuality, the arts etc.)

Spiritual fulfillment (experiencing faith, religiousness, and transcendence beyond ordinary material life)

Global perception of quality of life (feeling satisfied and happy with your life in general)

**3. References**

1.	Doornenbal BM, van Zutphen T, Beumeler LFE, Vos RC, Derks M, Haisma H, et al. Development and validation of a Context-sensitive Positive Health Questionnaire (CPHQ): A factor analysis and multivariate regression study. Journal of Patient-Reported Outcomes. 2024;8(1):44.

2.	Van Vliet M, Doornenbal BM, Boerema S, van den Akker-van Marle EM. Development and psychometric evaluation of a Positive Health measurement scale: a factor analysis study based on a Dutch population. BMJ Open. 2021;11(2):e040816.

3.	Hendrikx RJP, Drewes HW, Spreeuwenberg MD, Ruwaard D, Huuksloot M, Zijderveld C, et al. Measuring Population Health from a Broader Perspective: Assessing the My Quality of Life Questionnaire. Int J Integr Care. 2019;19(2):7.

4.	Haspels HN, de Vries M, van den Akker-van Marle ME. The assessment of psychometric properties for the subjective wellbeing-5 dimensions (SWB-5D) questionnaire in the general Dutch population. Qual Life Res. 2023;32(1):237–45.

5.	Al-Janabi H, Flynn TN, Coast J. Development of a self-report measure of capability wellbeing for adults: the ICECAP-A. Qual Life Res. 2012;21(1):167–76.

6.	Strijk JE, Wendel-Vos GCW, Picavet HSJ, Hofstetter H, Hildebrandt VH. Wat is vitaliteit en hoe is het te meten? Kerndimensies van vitaliteit en de Nederlandse vitaliteitsmeter. Tijdschrift voor gezondheidswetenschappen. 2015;93:32–40.

7.	Smith BW, Dalen J, Wiggins K, Tooley E, Christopher P, Bernard J. The brief resilience scale: assessing the ability to bounce back. Int J Behav Med. 2008;15(3):194–200.

8.	Khor S, Fuller-Tysziewicz M, Hutchinson D. Australian normative data. In: Personal Wellbeing Index Manual. Melbourne. : Australian Centre on Quality of Life, Deakin University.; 2020.

9.	Feng YS, Kohlmann T, Janssen MF, Buchholz I. Psychometric properties of the EQ-5D-5L: a systematic review of the literature. Qual Life Res. 2021;30(3):647–73.

10.	Foundation ER. EQ–5D–5L User Guide, 2025 [Available from: https://euroqol.org/information-and-support/documentation/user-guides/.

11.	Mezzich JE, Cohen NL, Ruiperez MA, Banzato CE, Zapata-Vega MI. The Multicultural Quality of Life Index: presentation and validation. J Eval Clin Pract. 2011;17(2):357–64.
