# Supplementary material for: Stakeholder and end-user perspectives to refine the Context-sensitive Positive Health Questionnaire (CPHQ) to measure broad health
Source: PLoS One. 2026 Aug 24;21(8):e0330014. doi: 10.1371/journal.pone.0330014 (PMC13502646; doi:10.1371/journal.pone.0330014)
Supplement: Supplementary file 3 [file pone.0330014.s003.docx]

**Appendix 3. Example prompts.**

| **Time** | **topic** | **Work form** | **Prompts** |
| --- | --- | --- | --- |
| 10 minutes | -Welcome & introduction -Practical aspects -Explanation process with quantitative scoring items (retain, adjust, omit) and qualitative scoring (memos and golden tickets), the posters + explain the hand book. | Presentation | -What is (broad) health |
| 20 minutes | -Expectations of the day? -When we measure health, what do we need to capture? -Preconditions of a measuring instrument/questionnaire? -Fill out questionnaire | Open discussion and paper questionnaire | -Measuring health *How do we do that? What does health consist of? What do we need to measure health? What not? -What preconditions are needed for a measuring instrument / questionnaire to measure broad health?* |
| 30 minutes | Round 1: quantitative scoring of the items on all 4 posters | Carrousel (in small groups under supervision of researcher) |  |
| 30 minutes | Round 2: qualitative feedback on the items on all 4 posters | Carrousel (in small groups under supervision of researcher) |  |
| 20 minutes | -Summary of input: e.g., where is strong agreement, where do opinions differ, new suggestions -Essential feedback still missing -Answer options -Length -Readability |  |  |
| 10 minutes | -Tips and tops to improve future sessions -Closure and thank the participants and if applicable vouchers and travel reimbursement) | Plenary |  |
