# Supplementary material for: Stakeholder and end-user perspectives to refine the Context-sensitive Positive Health Questionnaire (CPHQ) to measure broad health
Source: PLoS One. 2026 Aug 24;21(8):e0330014. doi: 10.1371/journal.pone.0330014 (PMC13502646; doi:10.1371/journal.pone.0330014)
Supplement: Supplementary file 4 [file pone.0330014.s004.docx]

**Appendix 4. Member check.**

Please note that the member check questions are originally in Dutch and translated to English.

| **Please indicate on a scale from 1-10 how much you agree with the questions.** | 0= no agreement, 10=full agreement | | | | | | | | | | |
| --- | --- | --- | --- | --- | --- | --- | --- | --- | --- | --- | --- |
|  | 0 | 1 | 2 | 3 | 4 | 5 | 6 | 7 | 8 | 9 | 10 |
| What do you think of the readability of the questionnaire? |  |  |  |  |  |  |  |  |  |  |  |
| What do you think of the understandability of the questionnaire? |  |  |  |  |  |  |  |  |  |  |  |
| Are the subheadings for categorized questions clear? |  |  |  |  |  |  |  |  |  |  |  |
| Do you have any suggestions or adjustments that you feel we need to make? |  | | | | | | | | | | |

**Please indicate your preference for an answer option?**

| Indicate your preference below | Example question |  | | | | |
| --- | --- | --- | --- | --- | --- | --- |
|  | I feel healthy | No I don’t feel healthy at all | No I don’t feel healthy | I sometimes feel healthy | Yes I feel healthy | Yes I feel entirely healthy |
|  | | | | | | |
|  | I feel healthy |  |  |  |  |  |
