# Supplementary material for: Stakeholder and end-user perspectives to refine the Context-sensitive Positive Health Questionnaire (CPHQ) to measure broad health
Source: PLoS One. 2026 Aug 24;21(8):e0330014. doi: 10.1371/journal.pone.0330014 (PMC13502646; doi:10.1371/journal.pone.0330014)
Supplement: Supplementary file 5 [file pone.0330014.s005.docx]

**Appendix 5. Worked out example.**

Example Item nr 7: I can move easily, such as climbing stairs, walking or cycling.

Step 1: we applied the decision rules per focus group and subsequently across focus groups
An item was retained when >50% scored 1 (green) and ≤15% 3 (orange), adjusted when >50% scored 2 (yellow) and ≤15% 1 (green), and omitted when >50% scored 3 (orange) and ≤15% 1 (green).

First the decision rules were applied to the individual focus groups. The patient focus groups were analyze together due to a small number of participants in one of the focus groups. See table 1 for an overview of decision in step 1.

| **Table 1.** overview decisions in step 1. | |
| --- | --- |
| Experts | No consensus |
| National policymakers | No consensus |
| Regional policymakers & welfare | Retain |
| Medical specialists | Adjust |
| Paramedics | No consensus |
| Mixed | No consensus |
| Patients | Retain |
| Citizens | Retain |
| Citizens lower SEP | Retain |
| Overall | No consensus |

This step yielded the following results:
-In four focus groups there was consensus to retain the item.
-In one focus groups there was consensus to adjust the item.
- In four of the focus groups there was no consensus.

Subsequently it was examined whether there was consensus across all focus groups. For item 7, there was no consensus. Therefore, the core group decided to go on with step 2.

Step 2: Examine qualitative feedback and golden tickets for this item.
In step 2 all the qualitative feedback on the memo’s was grouped for this item. This yielded some key themes. The themes that were identified were:

-Mentioning that it was a good item
-Omission of examples
-Mentioning that it is more important to get to the place where you want or need to be whether that is via walking or a wheel chair.
-That normative phrasing should be avoided and that for this item the word ‘easily” may be normative.

There was 1 golden ticket given to this item.

Step 3: Decide to adjust the item based on qualitative feedback.
The core group discussed until consensus as reached about the item. At least four out of six core group members had to agree to an adjustment or inclusion of an item for consensus. Based on all the qualitative input and golden tickets the item was rephrased to “I can move”. For this item it was decided to omit the examples and the normative phrasing and thereby also making the item shorter and enhance readability.
