# Supplementary material for: Stakeholder and end-user perspectives to refine the Context-sensitive Positive Health Questionnaire (CPHQ) to measure broad health
Source: PLoS One. 2026 Aug 24;21(8):e0330014. doi: 10.1371/journal.pone.0330014 (PMC13502646; doi:10.1371/journal.pone.0330014)
Supplement: Supplementary file 6 [file pone.0330014.s006.docx]

**Appendix 6. 28-item CPHQ.**

This questionnaire is developed in the Netherlands and in the Dutch language. All final items were translated to English by a native speaker from a translating agency. Back-to-back translation did not take place.

| **Table 1.** Definitive list of items of the improved 28-CPHQ in Dutch and in English categorized according to subheadings. | | |
| --- | --- | --- |
|  | **Dutch** | **English** |
| **Item** |  |  |
|  | **Mijn omgeving** | **My environment** |
| 1 | Ik heb mensen met wie ik leuke dingen kan doen | I have people I can do fun things with |
| 2 | Ik heb geld om gezond te leven | I have money to live a healthy life |
| 3 | Ik heb geld om de dingen die ik belangrijk vind te doen | I have money to do the things I find important |
| 4 | Ik heb mensen die mij steunen | I have people who support me |
| 5 | Ik voel me buitengesloten* | I feel excluded* |
| 6 | Ik voel me geaccepteerd | I feel accepted |
| 7 | Ik voel dat mensen mij steunen | I feel that people support me |
| 8 | Ik voel dat ik erbij hoor | I feel that I belong |
| 9 | Ik voel mij dankbaar voor het leven | I feel grateful for my life |
| 10 | Ik voel mij veilig waar ik woon | I feel safe where I live |
| 11 | Ik heb vertrouwen in de samenleving | I have confidence in society |
| 12 | Ik heb doelen en idealen die ik wil bereiken | I have goals and ideals I want to achieve |
| **Item** | **Waar ik toe in staat ben** | **What I am capable of doing** |
| 13 | Ik kan ontspannen | I can relax |
| 14 | Ik kan doen wat ik belangrijk vind | I can do what I think is important |
| 15 | Ik kan bewegen | I can move |
| 16 | Ik kan genieten | I can enjoy |
| 17 | Ik kan omgaan met tegenslagen | I can cope with setbacks |
| 18 | Ik kan actief zijn in mijn omgeving | I can be active in my environment |
| 19 | IK kan hulp vinden voor mijn gezondheid | I can find help for my health |
| 20 | Ik kan informatie over mijn gezondheid begrijpen | I can understand information about my health |
| 21 | Ik kan werken/vrijwilligerswerk doen of mantelzorg geven | I can work/volunteer or provide informal care |
| 22 | Ik kan mijn grenzen aangeven | I can express my boundaries |
| **Item** | **Ervaren gezondheid** | **Perceived health** |
| 23 | Ik voel mij gezond | I feel healthy |
| 24 | Ik voel mij fit | I feel fit |
| 25 | Ik zit lekker in mijn vel | I feel good about myself |
| 26 | Ik voel mij rustig | I feel calm |
| 27 | Ik voel mij gelukkig | I feel happy |
| 28 | Ik voel dat mijn leven zinvol is | I feel my life is meaningful |

*Negative formulation.
