# Supplementary material for: Stakeholder and end-user perspectives to refine the Context-sensitive Positive Health Questionnaire (CPHQ) to measure broad health
Source: PLoS One. 2026 Aug 24;21(8):e0330014. doi: 10.1371/journal.pone.0330014 (PMC13502646; doi:10.1371/journal.pone.0330014)
Supplement: Supplementary file 7 [file pone.0330014.s008.docx]

Rebuttal letter: PONE-D-25-39699
Stakeholder and end-user perspectives to improve the Context-sensitive Positive Health Questionnaire (CPHQ) to measure broad health

Dear editors and reviewers,
We would like to thank you for your constructive feedback on our manuscript. We appreciate the time and effort that you took to review our manuscript. We have carefully considered all your comments and revised our manuscript accordingly. We believe that these revisions improved the clarity and quality of our manuscript. Below, we provide a point-by-point response to each comment. Reviewer and editor comments are in black font color followed by our response and a description of our changes in blue. We hope that the revised version meets your expectations and we look forward to your response.

On behalf of all authors,
Cheryl Roumen and Mirte Boelens

Journal requirements:

When submitting your revision, we need you to address these additional requirements.

1. Please ensure that your manuscript meets PLOS ONE's style requirements, including those for file naming. The PLOS ONE style templates can be found at https://journals.plos.org/plosone/s/file?id=wjVg/PLOSOne_formatting_sample_main_body.pdf and <https://journals.plos.org/plosone/s/file?id=ba62/PLOSOne_formatting_sample_title_authors_affiliations.pdf>
We adjusted our files accordingly.

2. Thank you for stating the following in the Funding Section of your manuscript:

“This work was funded by a research grant (project number: 10530012110002) from ZonMw, The Netherlands Organization for Health Research and Development(https://www.zonmw.nl/en). ZonMw has no role in the study design, data collection and analysis, decision to publish or preparation of the manuscript.”

We note that you have provided funding information that is currently declared in your Funding Statement. However, funding information should not appear in the Acknowledgments section or other areas of your manuscript. We will only publish funding information present in the Funding Statement section of the online submission form.

Please remove any funding-related text from the manuscript and let us know how you would like to update your Funding Statement. Currently, your Funding Statement reads as follows:

“his work was funded by a research grant (project number: 10530012110002) from ZonMw, The Netherlands Organization for Health Research and Development (<https://www.zonmw.nl/en>). ZonMw has no role in the study design, data collection and analysis, decision to publish or preparation of the manuscript.”

Please include your amended statements within your cover letter; we will change the online submission form on your behalf.

We have deleted funding information from the acknowledgement section and other areas of the manuscript.
In the cover letter we ensured an update of our funding information. The funding information we would like to report is as follows: “This work was funded by a research grant (project number: 10530012110002) from ZonMw, The Netherlands Organization for Health Research and Development (https://www.zonmw.nl/en). ZonMw has no role in the study design, data collection and analysis, decision to publish or preparation of the manuscript.”.

3. In the online submission form you indicate that your data is not available for proprietary reasons and have provided a contact point for accessing this data. Please note that your current contact point is a co-author on this manuscript. According to our Data Policy, the contact point must not be an author on the manuscript and must be an institutional contact, ideally not an individual. Please revise your data statement to a non-author institutional point of contact, such as a data access or ethics committee, and send this to us via return email. Please also include contact information for the third party organization, and please include the full citation of where the data can be found.
We have edited the point of contact to the institutional point of contact of the Health Campus the Hague, department of Public Health and Primary Care at the Leiden University Medical Center via: [LUMC-CampusDenHaag@lumc.nl](mailto:LUMC-CampusDenHaag@lumc.nl).
Furthermore, (meta)data is uploaded in a repository (<https://doi.org/10.34894/9STE1Z>, [Ontwikkelen Meetinstrumentarium Brede Benadering Gezondheid](https://dataverse.nl/dataverse/breedmeten)).

4. Please include captions for your Supporting Information files at the end of your manuscript, and update any in-text citations to match accordingly. Please see our Supporting Information guidelines for more information: <http://journals.plos.org/plosone/s/supporting-information>.
We have added Supporting Information files at the end of our manuscript. We adjusted in-text citations accordingly.

If the reviewer comments include a recommendation to cite specific previously published works, please review and evaluate these publications to determine whether they are relevant and should be cited. There is no requirement to cite these works unless the editor has indicated otherwise. 
Thank you for pointing this out. The reviewer has not recommended to cite specific previously published work.

Reviewers' comments:
Reviewer's Responses to Questions

Comments to the Author
1. Is the manuscript technically sound, and do the data support the conclusions?
The manuscript must describe a technically sound piece of scientific research with data that supports the conclusions. Experiments must have been conducted rigorously, with appropriate controls, replication, and sample sizes. The conclusions must be drawn appropriately based on the data presented.

Reviewer #1: Yes

2. Has the statistical analysis been performed appropriately and rigorously?

Reviewer #1: N/A

3. Have the authors made all data underlying the findings in their manuscript fully available?
The [PLOS Data policy](https://eur03.safelinks.protection.outlook.com/?url=http%3A%2F%2Ftrack.editorialmanager.com%2FCL0%2Fhttp%3A%252F%252Fwww.plosone.org%252Fstatic%252Fpolicies.action%2523sharing%2F1%2F010f0199e68bd8d7-ecacca60-12c8-4bf1-94de-98560fa1fc3a-000000%2FaKRUXS_lrtukVpo283HeBf6pXcwD6zpdiCoGFrtyBwQ%3D233&data=05%7C02%7Cm.boelens%40lumc.nl%7C76547e7d203049d3c62d08de0bb3af3c%7Cc4048c4fdd544cbd80495457aacd2fb8%7C0%7C0%7C638961063816764281%7CUnknown%7CTWFpbGZsb3d8eyJFbXB0eU1hcGkiOnRydWUsIlYiOiIwLjAuMDAwMCIsIlAiOiJXaW4zMiIsIkFOIjoiTWFpbCIsIldUIjoyfQ%3D%3D%7C0%7C%7C%7C&sdata=mMtdl0cd%2FHR3%2Fu0%2FE%2BhDho1IuhUomOEneHbgWN6FVuc%3D&reserved=0) requires authors to make all data underlying the findings described in their manuscript fully available without restriction, with rare exception (please refer to the Data Availability Statement in the manuscript PDF file). The data should be provided as part of the manuscript or its supporting information, or deposited to a public repository. For example, in addition to summary statistics, the data points behind means, medians and variance measures should be available. If there are restrictions on publicly sharing data—e.g. participant privacy or use of data from a third party—those must be specified.

Reviewer #1: Yes

4. Is the manuscript presented in an intelligible fashion and written in standard English?
PLOS ONE does not copyedit accepted manuscripts, so the language in submitted articles must be clear, correct, and unambiguous. Any typographical or grammatical errors should be corrected at revision, so please note any specific errors here.

Reviewer #1: Yes

5. Review Comments to the Author
Please use the space provided to explain your answers to the questions above. You may also include additional comments for the author, including concerns about dual publication, research ethics, or publication ethics. (Please upload your review as an attachment if it exceeds 20,000 characters)

*Reviewer #1: 
1. Study aim vs. design clarity*
The paper oscillates between instrument refinement and qualitative exploration of item content. Tighten the aim in Abstract/Introduction and align Methods/Results accordingly (e.g., “to refine the 32-item CPHQ for cross-domain use through stakeholder focus groups, expert review, and member checking, producing a 28-item version ready for psychometric testing ).
We have tightened our aim in the abstract/introduction and aligned this aim with our methods/results accordingly. The objective in the introduction (see line 121-128) is adjusted to: *“The objective of this study is to further improve the CPHQ-instrument to make it more generally applicable and therefore accepted across different domains through focus group discussions (FGD) incorporating the perspectives of a broader group of stakeholders across healthcare, welfare and policy domains, researchers, as well as patients and citizens, including those from a lower SEP, expert review and a member check.“.*

*2. Sampling strategy and reflexivity*
Provide a more transparent account of purposive sampling: who identified participants, how invitations were distributed, and how diversity (domain, geography, SEP, gender) was ensured; add a brief table on recruitment sources and non-participation. Include researcher positionality/reflexivity and how it may have shaped facilitation and analysis (e.g., backgrounds of moderators, prior ties to Positive Health). This aligns with SRQR/COREQ good practice.
We acknowledge the information on the sampling process was rather brief. Therefore, we have added more detailed information about the sampling and recruitment in our manuscript. See line 168-171 in the methods sections “*A purposeful sampling approach was used. This meant that the participants for FGDs were recruited based on accessibility and based on a variety of professions and backgrounds and geographical areas to capture a wide range of perspectives. The distributors of the invitations were researchers who had access to the target population (see table 1).”* Furthermore, we added information in a table (table 1) in the methods section, see line 172,173 and below.

| **Table. 1** Recruitment of focus group discussions (FGDs) | | | | | | | |
| --- | --- | --- | --- | --- | --- | --- | --- |
| **Focus group** | **Target group** | **Distributor(s) of invitations** | **Invitations (n)** | **Participants (n)** | **Location** | **Date (dd-mm-yyyy)** | **Facilitators** |
| 1 | Medical specialists | LNvV | 15 | 10 | Den Bosch | 23-01-23 | CR, EB & MB |
| 2 | Regional policymakers, welfare | JS, JD | 33 | 14 | Eindhoven | 25-01-23 | JD, MB, EB |
| 3 | Citizens | JS and passive via hardcopy information letters in a primary care practice waiting room | 18 + passive | 8 | Eindhoven | 07-02-23 | EB, MB |
| 4 | Citizens | TvZ, PC | 15 | 8 | Leeuwarden | 24-02-23 | PC, TvZ, MB |
| 5 | National policymakers | JK, MS, JD, MdK, CR, EB and passive via social media | 28 + passive | 14 | Utrecht | 16-03-23 | EB, MB |
| 6 | Experts | CR, EM, MB MdK, and passive via social media | 33 + passive | 8 | Utrecht | 01-03-23 | CR, EB, MB |
| 7 | Paramedics | JS, MB and passive via social media | 60 + passive | 6 | Eindhoven | 23-03-23 | JS, CR, EB, MB |
| 8 | Mixed group ( policymakers and experts) | EB, MB, CR, mDK | 17 | 6 | Utrecht | 06-04-23 | EB, MB, CR |
| 9 | Patients | CR | 7 | 1 | Maastricht | 16-08-23 | CR, MB |
| 10 | Patients | TvZ, PC | 72 | 8 | Leeuwarden | 19-09-23 | PC, TvZ, MB |

Furthermore, we agree with the reviewer that information on researcher positionality/reflexivity is important. We added information about researcher positionality/reflexivity in the methods section, see line 287-311
“Researcher positionality/reflexivity
The research consortium consisted of researchers with diverse backgrounds. The core group (MS^1^, JK^2^, MB^3^, CR^4^, EB^5^, JD^6^) included six project members with expertise in psychology and statistics^1^, population health^2,3^, broad health concepts^1,2,5^, human biology and participatory design within oncology^4^, nutritional sciences and public health^2,3,4,5,^, and participatory research and shared decision-making^6^. All consortium members were familiar with the frameworks of Positive Health. Especially JD had expertise on the Capability Approach. While some researchers were advocates of Positive Health (EB^5^, JD^6^) as a way to capture a broader understanding of health, others (MS^1^, JK^2^, MB^3^ CR^4^) maintained a more critical stance, emphasizing conceptual clarity and empirical validity. This diversity of perspectives fostered reflexive dialogue throughout the research process.

Some consortium members (MdK, MvV) were affiliated with the Institute of Positive Health (iPH), and one member (EB) was a certified Positive Health trainer. These positions provided valuable insight and practical expertise regarding Positive Health, but could also introduce a tendency toward interpreting the questionnaire and results in alignment with the framework.

The focus group discussions were facilitated by different consortium members (CR, MB, EB, LNvV, TvZ, PMRC, JS). There were always 2 and sometimes 3 discussion leaders depending on who was most familiar with the target group.

Data were interpreted by two lead researchers (CR, MB) with complementary perspectives, and analytic decisions were discussed within the core group (MS, JK, CR, MB, EB, JD) to enhance reflexivity. Following analysis, the wider consortium and experts from the Dutch Positive Health Network reviewed and provided feedback on the questionnaire. While this consultation offered valuable expertise, it could potentially introduce confirmatory bias, favoring interpretations aligned with the Positive Health framework. Combining independent analysis, core group discussions, and external feedback helped mitigate potential bias and strengthened the credibility and trustworthiness of the findings.”.

*3. Focus group procedures and analytic rigour*
You describe two “rounds” (qualitative memos; sticker voting) plus thematic analysis and an adapted COMET decision rule. Please:
Cite or append the exact decision rules and thresholds and justify the >50% / ≤15% cut-offs; explain how discordant groups were handled.
The decision rules are inspired by an example given in the COMET handbook(1). The decision rules are originally used by Blazeby et al.(2) The COMET handbook describes these decision rules to retain or omit items as less stringent which reduces the likelihood of omitting items that may be important. In the COMET handbook they use a Delphi approach consisting of two rounds. They used more stringent criteria in their second round. Instead of a second round, we discussed discordant items within the core team. Discordant items were discussed as follows: First, one core team explained the opinion, doubts and consideration of a certain item, then a second core team member would do the same until all members had given their opinion. Then, the another core team member would give a suggestion and all members would respond to that in sequence. Finally, a parallel discussion took place in which all members participated until consensus was reached. To avoid bias, for different discordant questions, different core team members started the discussion.
The discussion about these items were based on the qualitative input written on the memos and the golden tickets.

We added the reference to the comet handbook by Paula Williamson (2017) and a reference to the paper by Blazeby et al (2015) in our manuscript, see line 219. We also added a more thorough explanation of our analysis in the manuscript, see line 222-253 *“**For each focus group all items were scored quantitatively by counting the colored stickers scored as 1 for green, 2 for yellow and 3 for orange. Number of golden tickets was scored per item. Decision rules were applied per item separately for each focus group.* *An item was retained when >50% scored 1 (green) and ≤15% 3 (orange), adjusted when >50% scored 2 (yellow) and ≤15% 1 (green), and omitted when >50% scored 3 (orange) and ≤15% 1 (green). If a score of an item fell outside these categories, the core group decided that it was inconclusive or proposed a suggestion if numbers were close to either keep, adjust omit.

 After this, in case of consensus about an item across the focus groups (when all focus groups score the same on an item), this score (retain, adjust, omit) was followed by the core group. If the scoring of an item was inconclusive (discordant items), the core group decided on retaining, adjusting, omitting or merging items when meaning and content overlapped based on the memos with qualitative input and the golden tickets.
The goal of the qualitative feedback was not to examine emerging themes but rather to examine what aspects of the CPHQ items need adaptation or adjustment and what is perceived as useful or not useful. Therefore, we grouped all qualitative feedback from all focus groups per item. We examined how often a certain type of feedback was given for each item, e.g. “this item is complicated, consider reformulating the item”.*

*Discordant items (items without consensus based on quantitative scoring) were discussed by the core group. Consensus was achieved through structured discussion until agreement was reached. For each item, a different core group member initiated the discussion by stating their preference and providing a rationale. Subsequently, the other members shared their views in a rotating order. At least four out of six core group members had to agree to an adjustment or inclusion of an item for consensus. This structured discussion format was deliberately applied to ensure that all core group members had equal opportunity to contribute and to mitigate potential dominance effects of power imbalances within the group. CR and MB lead the discussion.

 Items that were scored as ‘adjust’ were discussed by the core group and ultimately reformulated based on the qualitative feedback. Items that were scored as ‘retain’ and items there were merged were also sometimes reformulated to enhance overall readability and consistency throughout the questionnaire.”*

Clarify how qualitative inputs (memos/transcripts) were integrated with sticker scores to decide retain/adjust/omit/merge at item level; a worked example for one contentious item would help. Report coder training, codebook development, intercoder agreement (if calculated) or processes to reach consensus. Move Table 2 (themes guiding adjustments) earlier and link each theme to concrete item changes.
We added two appendices to our manuscript. Appendix 5 illustrates a worked out example of item number seven. We also added appendix 3 Example prompts which includes our topic list for the focus groups

Our aim was not to generate new themes but to examine if the original CPHQ and if so what aspects of the CPHQ need adaptation/adjustment. Therefore, we did not develop a codebook nor performed coder training. We also did not assess intercoder agreement. We scored quantitatively and complemented this with the qualitative feedback.

We grouped all qualitative feedback from all focus groups per item. We examined how often a certain type of feedback was given, e.g., this item is difficult, reformulating the item”. Discordant items (items without consensus based on quantitative scoring) were discussed by the core group. Consensus was achieved through structured discussion until agreement was reached. For each item, a different core group member initiated the discussion by stating their preference and providing a rationale. Subsequently, the other members shared their views in a rotating order. At least four out of six core group members had to agree to an adjustment or inclusion of an item for consensus. This structured discussion format was deliberately applied to ensure that all core group members had equal opportunity to contribute and to mitigate potential dominance effects of power imbalances within the group. CR and MB lead the discussion.

We have added a more thorough explanation of qualitative input and process to reach consensus in the manuscript. See also our response to comment 3.

Table 2 is now labeled as table 3 in the manuscript. Each theme is linked to a decision in table 4. This way, readers can see which themes led to changes in items.

*4. Theoretical mapping to the Capability Approach*
The recategorization into resources, conversion factors, capabilities, functionings is a major conceptual move. Provide a short, a priori mapping framework (definitions + decision rules) and a few exemplars that were debated (e.g., “I feel safe where I live” vs. “I can be active in my environment”). Consider an expert agreement check for the taxonomy.
We thank the reviewer for highlighting the conceptual importance the recategorization into resources, conversion factors, capabilities and functionings. We agree that this represents a crucial step. The categorization was guided a priori by the Capability Approach framework and by the original CPHQ(3) and shown in table 5 (now table 6). The categoriziation was done by the core team which includes expertise on the capability approach. Categorization was done based on the definitions of resources, conversion factors, capabilities, and functionings according to the capability approach(4, 5). All core team members coded the items independently as either a resource, conversion factor capability or functioning. After independently coding each item the core team discussed until consensus was reach about the appropriate categorization of each item. Please, note, however, this recategorization was not aimed at removing items but more linking them explicitly to the taxonomy the Capability Approach for which experts familiar with the Capability Approach were part of our research group.

We added an explanation in line 373-377.

*5. Language, readability, and cross-cultural validity*
You note items were developed in Dutch, translated to English by a native translator, with no back-translation. For an instrument intended for cross-domain and potentially cross-national use, justify the absence of back-translation and describe plans for formal linguistic validation and cognitive debriefing in future studies. Also, elaborate on how readability for low literacy was ensured beyond the PHAROS review (e.g., Flesch scores, cognitive interviewing).
We did not perform back-to-back translation because of the interdisciplinary and cross-domain nature of the instrument. In addition, the aim of back-translation is to check linguistic equivalence but can be easily biased through the different perceptions and meanings of items that could exist within such an instrument. For that reason we prioritized the formulation of items together with stakeholders rather than testing for textual symmetry.

Readability and low literacy was ensured in several ways. Besides the Pharos review, we included a target group in the focus group sessions with a low social-economic status and lower literacy, so that we made sure that also these people would be able to understand the questionnaire. Second, we did a member check in which we specifically asked to check for readability and understandability. Third, we did an additional check for readability by checking words in all three subheadings on B1-level status (the level which is considered appropriate to be understandable for all people) via the website: <https://ishetb1.nl/> (Is it B1?). This website is set up by Loo van Eck and klinkende taal, with the aim to improve readability. On the website of klinkende taal (<https://klinkendetaal.nl/klanten/>), one can see that customers are municipalities, living cooporations, wellbeing organizations, foundations, health insurers, all of which are relevant parties for our new CPHQ2.0 questionnaire. Not all words are in the dictionary. However, we tested 10 words from different subheadings of which all were considered B1-level. See table 2 for an overview.

| **Table 2** check B1 subheadings | | | | |
| --- | --- | --- | --- | --- |
| **Subcategory** | **Question** | **Word in Dutch** | **Word in English** | **B1-level** |
| My environment | 6 | Geaccepteerd | Accepted | Yes |
|  | 7 | Steunen | Support | Yes |
|  | 9 | Dankbaar | Thankful | Yes |
|  | 11 | Vertrouwen | Trust | Yes |
|  | 12 | Doelen | Goals | Yes |
| What I am capable of doing | 13 | Ontspannen | Relax | Yes |
|  | 19 | Gezondheid | Health | Yes |
|  | 22 | Grenzen | Barriers | Yes |
| Perceived health | 26 | Rustig | Calm | Yes |
|  | 28 | Zinvol | Meaningful | Yes |

*6. Member check scope and interpretation*
Response rate was 33% (18/55). Discuss non-response bias and whether respondents differed from the original focus-group sample.

Summarize what changed (if anything) after the member check, and justify keeping contested items (e.g., “I feel excluded” negative phrasing) beyond stating “no major concerns.” Consider a short table mapping member-check suggestions to disposition.

We thank the reviewer for this comment. We acknowledge that the low response rate (33%) can lead to response bias and added this to our discussion as a limitation, see line 531-536 *“Unfortunately, the response date for the member check was low which may cause non-response bias. We cannot examine whether these participants were representative of all FGD participants. It could be that response of FGD participant groups was unequal and that results reflect predominantly the perspectives of more accessible and articulate participants. In particular, (lower SEP) citizens and patients may have been less likely to participate in the member check compared with professionals. As a result, some perspectives might be underrepresented in the final feedback on new CPHQ items.”.*

The feedback included suggestions on the order of items, specific feedback on the formulation of items (e.g. to make it even more straightforward, advice to test it with Pharos) and on answer categories. For example, it was mentioned that the negatively worded item attracted attention as it was the only negatively worded item and that it could be difficult to understand. We decided however to leave this item in the questionnaire because vulnerable populations such as lower SEP citizens did not recognize themselves in their positively formulated counterparts. This was also previously observed by Doornenbal et al(3) .Another example was to further define certain items. We decided not to do this because in an earlier stage we decided to omit examples from the times based on the feedback we obtained in the focus groups.

Feedback on the answer options were used to choose the most suitable answers option together with feedback from the focus groups in a later stage of the research project. This lead to the decision to combine smileys with a text on a five-point scale. Feedback on sections headings was also used in a later stage of the project to reformulate the section headings and to ad a third subheading.

We added a new table (Table 8) that illustrates the feedback from the member check. See line, 417.

In the manuscript we also added more information as follows (line 406-420): *“Suggestions of participants concerned the answer format (5/18), section headings (3/18), formulation of items (7/18), categorization of items (3/18), target population (3/18), satisfaction (2/18), and applicability for secondary care (1/18) (see* ***table 8****). Feedback on the answer format and categorization of items to subheadings will be used for future improvement of the CPHQ. With regard to the grouping and the applicability, no adjustments were made since the capability essentials were included in the grouping and the instrument is designed also for other domains than secondary care. With regard to the formulation, two participants questioned whether the opposite item “I feel excluded” would need to be stated in a positive formulation. We decided however to leave this item in the questionnaire because vulnerable populations such as lower SEP citizens did not recognize themselves in their positively formulated counterparts. This was also previously observed by Doornenbal et al. Other remarks concerned the interpretation of words such as ‘grateful’ and ‘calm’, which is preference-sensitive and to further define certain items. We decided not to do this because in an earlier stage we decided to omit examples from the times based on the feedback we obtained in the focus groups”.*

| **Table 8.** Feedback received from participants of the member check. | | |
| --- | --- | --- |
| Type of feedback | Examples | Frequency of feedback |
| Answer format | Preference for using smileys, Preference for textual scales and Likert scales | 5/18 |
| Section headings | Clarity and conceptual fit of headings | 3/18 |
| Formulation of items | Comments on negative phrasing (I feel excluded) or ambiguity (grateful or calm). | 7/18 |
| Categorization | Suitability of items underneath a subheading | 3/18 |
| Target population | Understandability of items (ie. Understandability of ‘perceived’ health for people with limited literacy) and applicability in secondary care | 3/18 |
| Satisfaction with items | No comments, reply to state satisfaction | 2/18 |
| Applicability for secondary care | Doubts about suitability for secondary care | 1/18 |

*7. Political representation & trust items*
Decisions around removing “I feel represented by politics” but retaining “confidence in society” merit deeper justification, given prior factor structure in the original CPHQ and the Capability Approach emphasis on social conversion factors. Consider keeping both for psychometric testing, or provide empirical/analytic rationale for removal beyond stakeholder preference.

We thank the reviewer for this valuable comment. The decision to remove the item “I feel represented by politics” and retain the item “I have confidence in society” is grounded on the focus group data. There was consensus about omission of the item “I feel represented by politics” based on the quantitative analysis. This was not the case for the item “I have confidence in society”. Furthermore, based on the qualitative analysis the input the item “I feel represented by politics” was considered as difficult to interpret, overly abstract an not directly relevant to the daily experiences of responders (in case of citizens and patients). In contrast, the other item “I have confidence in society” yielded less unambiguous feedback. For example, it was also considered as important because of the relation with social trust. Therefore, we decided to retain but adjust the item.

Conceptually, the item “confidence in society” may capture social trust and aligns with the capability approach conversion factors. The other item “I feel represented by politics”, although indeed theoretically relevant, was judged as less interpretable by our target population.

We agree that it would be interesting to explore both items in future psychometric testing, but we feel that for the current instrument, retention of the item “confidence in society” and omission of the item “I feel represented by politics” is justified.

We adjusted the paragraph discussing these items, see line 470-494: *“Participants also discussed the item “I feel represented by politics”, and agreed that it should be removed, albeit for different reasons. Medical professionals questioned its relevance, as it was perceived to lie beyond their professional influence. Patients and citizens felt that it was unrelated to their personal health. In the qualitative feedback, the item was described as abstract and difficult to interpret. Interestingly, a related item ”I have confidence in society” remained in the revised CPHQ. Previous research shows a link between poor health with lower political trust(6). Mattilla (2020) suggests that one reason for this lower trust might be a disconnect between what citizens expect from the government and institutions and how they actually perceive its outcomes, particularly regarding how fairly they feel treated(6). From a Capability Approach perspective, political representation can act as a conversion factor that shapes people's ability to achieve valued states(5). Moreover, in the original CPHQ, items on political representation formed a distinct factor in the factor analysis(3). These differing perspectives reflect the complexity of determining which contextual and societal factors should be included in a measure of broad health. We based our inclusion of the item “confidence in society” and omission of “political representation” on our a priori set decision rules. We acknowledge that the omission of a politically tinged item, may limits the breadth of the construct measured. While the retained item “I have confidence in society” may capture social trust, conversion factors concerning politics are less directly assessed. Our decision possibly reflects a trade-off between construct completeness and interpretability as participants found the item about political representation difficult to understand and abstract. We therefore recommend that future validation studies further explore whether and how aspects like political representation and trust in institutions contribute to people’s perceived capabilities for (broad) health. Rather than including these items by default, their relevance may need to be assessed contextually, depending on the population or on the purpose of the measurement.”.*

We also added it to our limitations as follows (line 538-548): *“Despite efforts to recruit a diverse group of stakeholders from different professional backgrounds and geographical regions within the Netherlands, it cannot be ruled out that participants were more positively included toward Positive Health as one of the concepts of broad health. To reduce this risk, during recruitment we explicitly invited both proponents and critics and FGD facilitators actively stimulated critical discussion. Nevertheless, this potential bias should be taken into account when interpreting the findings. The omission of the item “I feel represented by politics” may reduce coverage of a conversion factor that theoretically could influence health. This reflects a trade-off between interpretability and construct completeness as the item was perceived as difficult and abstract. Future validation studies could explore whether including this item adds meaningful information, depending on the context, target population of purpose of the measurement.”.*

*8. Data availability and transparency*
The current Data Availability Statement indicates restricted access due to privacy. Provide a stronger transparency plan (e.g., share de-identified sticker tallies, decision logs for each item in a public repository; provide the final 28-item instrument, Dutch + English, with response options). This aligns with PLOS ONE policy and will aid replication.
We thank the reviewer for this comments and acknowledge the importance of transparency for replicability. The metadata of our research project is uploaded in a repository (<https://doi.org/10.34894/9STE1Z>, [Ontwikkelen Meetinstrumentarium Brede Benadering Gezondheid](https://dataverse.nl/dataverse/breedmeten)). We will upload our de-identified sticker tallies and decision logs. We also provide our CPHQ instrument in Dutch and English in appendix 6. Because we did not perform our research with response options we provide the questionnaire without response options.

Abstract: Report key numbers succinctly (10 FGDs; n=76; final 28 items; main themes guiding edits). Avoid method jargon (“adapted COMET”) without a one-line gloss.
Thank you. We updated the results in the abstract accordingly from:

 *“The refined CPHQ consists of 28 items. Items were omitted, merged or added. Participants emphasized the importance of avoiding normative phrasing and including subjectively interpretable items. Items reflecting aspects of experienced health (e.g., “I feel happy” or “I feel healthy”) were prioritized, highlighting the relevance of functionings. At the same time, views diverged on the inclusion and wording of items related to exclusion, political representation and sense of belonging.“*

To:

*“Items were omitted, merged or added leading to a refined CPHQ (final 28 items). Main themes guiding edits were 1) the importance of avoiding normative phrasing and including subjectively interpretable items, 2) prioritization of items reflecting aspects of experienced health (e.g., “I feel happy” or “I feel healthy”), 3) highlighting the relevance of functionings. At the same time, views diverged on the inclusion and wording of items related to exclusion, political representation and sense of belonging.“.*

Methods: Specify focus-group duration per stakeholder type; clarify whether mixed-stakeholder groups were analyzed separately. Add example prompts and the “handbook” list to an SI.

Each focus group had a duration of 2 hours. The duration was not different between stakeholder types. We added this to the manuscript, see line, 315.

There was one mixed focus group consisting of policy makers and experts. This group was analyzed as a whole and not separated into different subgroups because the result of the focus group was based on individual opinions and voting, but also the interaction between the group members, e.g. sometimes members within a focus group changed their minds based on the various discussion points. Therefore, it was scientifically sound to analyze on group level and not on individual or subgroup level.

We have added a supplement with the script of the focus groups and a supplement with the hand book.

Results:
Tables 3–5 are valuable. Add a column in Table 3 linking each edit to the qualitative theme and COMET outcome; indicate where “golden tickets” influenced decisions.
We linked each edit to the corresponding qualitative themes and indicated whether “golden tickets” influenced decisions in the table. The table is now labeled as table 4.

Report descriptive characteristics per group consistently; a CONSORT-like flow for recruitment would help readability.
We added more information about the recruitment in the manuscript and in table 2. See line 172-173 and our response to comment 3.

Discussion:
When noting the primacy of functionings items (“I feel healthy/happy/fit”), link this more explicitly to measurement implications (risk of redundancy with global health/QoL items; need to test higher-order factor).
We agree that this is important to acknowledge. Therefore we have included the following in the implication of the discussion (line 564-569):

*“The revised CPHQ offers a promising tool to measure broad health. It could provide valuable insights in the effectiveness of health and welfare interventions or (policy) programs that are currently assessed from a more narrow perspective.* *In particular the functioning items are of added value on top of patient report outcomes or biomedical indicators as it reflects real opportunities and lived health instead of biomedical status and limitations.”.*

Limitations: Acknowledge possible pro-Positive-Health sample bias; discuss implications of removing politically tinged items for construct coverage.
-primacy of functionings…
- implications for monitoring population broad health vs. program evaluation, including sensitivity to change….

Indeed, we cannot rule out possible pro-Positive-Health sample bias. We shortly addressed this in our limitations, however, we agree that due to the importance we can elaborate on this potential bias.
In line 510-517 we discuss the potential bias *“There may be some recruitment bias towards individuals who are already more positive towards the concept of broad health. Despite efforts to recruit a diverse group of stakeholders from different professional backgrounds and geographical regions within the Netherlands, it cannot be ruled out that participants were more positively included toward Positive Health as one of the concepts of broad health. To reduce this risk, during recruitment we explicitly invited both proponents and critics and FGD facilitators actively stimulated critical discussion. Nevertheless, this potential bias should be taken into account when interpreting the findings.”.*

We elaborated on the implications of removing politically tinged items from our questionnaire. See also our response to comment 7.

We also elaborated on primacy of functionings in our discussion and adjusted the paragraph from: *“Another commonality was that the item “I feel healthy” was perceived as the most important item by participants. Other frequently emphasized items included “I feel fit,” “I feel good,” and “I feel happy”. Interestingly, these items reflect functionings in the terms of the Capability approach: they describe actual states of being and doing(4). Some of these items such as “feel healthy” or “I feel happy” are also used as standalone indicators in instruments that measure health, quality of life or well-being. For instance, the EQ-5D-5L includes a visual analogue scale (VAS) where respondents rate how healthy they feel, and single-item measures or happiness are also used in population health surveys(7, 8). This suggests that such functionings may not only serve as outcomes but also as overarching, integrative indicators of health. However, according to the Capability Approach, what matters is not only the realization of these experienced states but the freedom and opportunity (i.e. capabilities) to achieve them. Instruments that focus solely on functionings may miss whether people actually had the opportunity to achieve these states, while instruments that only assess capabilities might miss how people actually perceive their health. Therefore, we suggest that to assess broad health, a measurement instrument should include both elements.”*

to (line 470-494): *“Participants also discussed the item “I feel represented by politics”, and agreed that it should be removed, albeit for different reasons. Medical professionals questioned its relevance, as it was perceived to lie beyond their professional influence. Patients and citizens felt that it was unrelated to their personal health. In the qualitative feedback, the item was described as abstract and difficult to interpret. Interestingly, a related item ”I have confidence in society” remained in the revised CPHQ. Previous research shows a link between poor health with lower political trust(6). Mattilla (2020) suggests that one reason for this lower trust might be a disconnect between what citizens expect from the government and institutions and how they actually perceive its outcomes, particularly regarding how fairly they feel treated (Mattila, 2020). From a Capability Approach perspective, political representation can act as a conversion factor that shapes people's ability to achieve valued states(5). Moreover, in the original CPHQ, items on political representation formed a distinct factor in the factor analysis(3). These differing perspectives reflect the complexity of determining which contextual and societal factors should be included in a measure of broad health. We based our inclusion of the item “confidence in society” and omission of “political representation” on our a priori set decision rules. We acknowledge that the omission of a politically tinged item, may limits the breadth of the construct measured. While the retained item “I have confidence in society” may capture social trust, conversion factors concerning politics are less directly assessed. Our decision possibly reflects a trade-off between construct completeness and interpretability as participants found the item about political representation difficult to understand and abstract. We therefore recommend that future validation studies further explore whether and how aspects like political representation and trust in institutions contribute to people’s perceived capabilities for (broad) health. Rather than including these items by default, their relevance may need to be assessed contextually, depending on the population or on the purpose of the measurement.”*

We added the following to our limitation sections about implications for monitoring population health vs. program evaluation (line 558-561): “Finally, while the CPHQ is meant to be both suitable to assess broad health using population monitoring and program evaluation. Sensitivity to detect short and longer term changes is not yet known and remains to be confirmed.”

Presentation: Fix small typos (“pronfessionals”, spacing), standardize item wording (all first-person present), and ensure the final 28-item list in Appendix 3 exactly matches Table 5 categories.
Thank you for pointing this out. We have adjusted our manuscript accordingly. We ensured the 28-item list in Appendix 3 (now appendix 6) exactly matches the items in table 5 (now table 6) of the manuscript.

**References**

1.	Williamson PR, Altman DG, Bagley H, Barnes KL, Blazeby JM, Brookes ST, et al. The COMET Handbook: version 1.0. Trials. 2017;18(Suppl 3):280.

2.	Blazeby JM, Macefield R, Blencowe NS, Jacobs M, McNair AG, Sprangers M, et al. Core information set for oesophageal cancer surgery. Br J Surg. 2015;102(8):936–43.

3.	Doornenbal BM, van Zutphen T, Beumeler LFE, Vos RC, Derks M, Haisma H, et al. Development and validation of a Context-sensitive Positive Health Questionnaire (CPHQ): A factor analysis and multivariate regression study. Journal of Patient-Reported Outcomes. 2024;8(1):44.

4.	Nussbaum MC. Creating capabilities: The human development approach. Harvard University Press; 2011.

5.	Robeyns I. The Capability Approach: a theoretical survey. Journal of Human Development. 2005;6(1):93–117.

6.	Mattila M. Does poor health mobilize people into action? Health, political trust, and participation. European Political Science Review. 2020;12(1):49–65.

7.	Andrews FM, Withey SB. Social indicators of well-being: Americans' perceptions of life quality: Springer Science & Business Media; 2012.

8.	Feng YS, Kohlmann T, Janssen MF, Buchholz I. Psychometric properties of the EQ-5D-5L: a systematic review of the literature. Qual Life Res. 2021;30(3):647–73.
