# Supplementary material for: Stakeholder and end-user perspectives to refine the Context-sensitive Positive Health Questionnaire (CPHQ) to measure broad health
Source: PLoS One. 2026 Aug 24;21(8):e0330014. doi: 10.1371/journal.pone.0330014 (PMC13502646; doi:10.1371/journal.pone.0330014)
Supplement: Supplementary file 8 [file pone.0330014.s009.docx]

Rebuttal letter: PONE-D-25-39699
Stakeholder and end-user perspectives to improve the Context-sensitive Positive Health Questionnaire (CPHQ) to measure broad health

Dear editors and reviewers,

We would like to thank you for your constructive feedback on our manuscript. We appreciate the time and effort that you took to review our manuscript. We have carefully considered all your comments and revised our manuscript accordingly. We believe that these revisions improved the clarity and quality of our manuscript. Below, we provide a point-by-point response to each comment. Reviewer and editor comments are in black font color followed by our response and a description of our changes in blue. We hope that the revised version meets your expectations and we look forward to your response.

On behalf of all authors,
Cheryl Roumen and Mirte Boelens

**Comments to the Author**

1. If the authors have adequately addressed your comments raised in a previous round of review and you feel that this manuscript is now acceptable for publication, you may indicate that here to bypass the “Comments to the Author” section, enter your conflict of interest statement in the “Confidential to Editor” section, and submit your "Accept" recommendation.

Reviewer #2: All comments have been addressed

Reviewer #3: All comments have been addressed

2. Is the manuscript technically sound, and do the data support the conclusions?

The manuscript must describe a technically sound piece of scientific research with data that supports the conclusions. Experiments must have been conducted rigorously, with appropriate controls, replication, and sample sizes. The conclusions must be drawn appropriately based on the data presented.

Reviewer #2: Yes

Reviewer #3: Yes

3. Has the statistical analysis been performed appropriately and rigorously?

Reviewer #2: Yes

Reviewer #3: Yes

4. Have the authors made all data underlying the findings in their manuscript fully available?

The [PLOS Data policy](https://eur03.safelinks.protection.outlook.com/?url=http%3A%2F%2Ftrack.editorialmanager.com%2FCL0%2Fhttp%3A%252F%252Fwww.plosone.org%252Fstatic%252Fpolicies.action%2523sharing%2F1%2F010f019d08d63bf1-234d0fd6-433e-42a2-a697-8f5be49d9a1a-000000%2FlM4oRUat7sCOe3jozYQUmPfhhN9tSYy5FoI95oGzcKo%3D255&data=05%7C02%7Cm.boelens%40lumc.nl%7C07c27a141813476d8c5408de8e6505ce%7Cc4048c4fdd544cbd80495457aacd2fb8%7C0%7C0%7C639104761729624453%7CUnknown%7CTWFpbGZsb3d8eyJFbXB0eU1hcGkiOnRydWUsIlYiOiIwLjAuMDAwMCIsIlAiOiJXaW4zMiIsIkFOIjoiTWFpbCIsIldUIjoyfQ%3D%3D%7C0%7C%7C%7C&sdata=JQGL%2FU31AxcG3JKYVhJxNdnad5M1sPBJKeKs2v1Zz6s%3D&reserved=0) requires authors to make all data underlying the findings described in their manuscript fully available without restriction, with rare exception (please refer to the Data Availability Statement in the manuscript PDF file). The data should be provided as part of the manuscript or its supporting information, or deposited to a public repository. For example, in addition to summary statistics, the data points behind means, medians and variance measures should be available. If there are restrictions on publicly sharing data—e.g. participant privacy or use of data from a third party—those must be specified.

Reviewer #2: Yes

Reviewer #3: No

5. Is the manuscript presented in an intelligible fashion and written in standard English?

PLOS ONE does not copyedit accepted manuscripts, so the language in submitted articles must be clear, correct, and unambiguous. Any typographical or grammatical errors should be corrected at revision, so please note any specific errors here.

Reviewer #2: Yes

Reviewer #3: Yes

6. Review Comments to the Author

Please use the space provided to explain your answers to the questions above. You may also include additional comments for the author, including concerns about dual publication, research ethics, or publication ethics. (Please upload your review as an attachment if it exceeds 20,000 characters)

Reviewer #2: My review comments are based on Revision 1 which includes multiple changes made in response to Reviewer 1's feedback. These changes have been substantively met, and no further changes are recommended. As noted by the authors, a well-designed patient-reported outcome measure is an important step in supporting the potential utility of positive health perspectives. The continued development of the CPHQ is an important step in this direction. Future research from independent research groups to clarify the reliability and validity of this measure will be an important outcome that this paper supports.
Thank you for your review of our manuscript and your considerations!

Reviewer #3: Thank you for the opportunity to evaluate this manuscript. In this work, the authors aimed to refine the 32-item Context-sensitive Positive Health Questionnaire (CPHQ) for broader use across healthcare, welfare, and policy domains. Starting from the previously developed CPHQ, they conducted focus group discussions with stakeholders from healthcare, welfare, policy, and research, as well as with patients and citizens, including individuals with lower socioeconomic position. The study combined qualitative feedback and structured item scoring during the focus groups. Participants commented on the relevance, clarity, and formulation of questionnaire items, rated whether items should be retained, adjusted, or omitted, and identified particularly important items. Based on this process, the authors omitted, merged, reformulated, and added items, resulting in a revised set of 28 items. After the focus groups, the revised questionnaire was further examined through expert validation and a member check with a subgroup of original participants. The expert review contributed to additional refinements in item wording and supported the inclusion of some items that had been reconsidered as important for capturing broad health. The final set of items was then categorized according to the Capability Approach into resources, conversion factors, capabilities, and functionings. The final result of the study is a revised 28-item CPHQ, intended as a theory-informed and stakeholder-informed instrument to measure broad health, with future research needed for further validation and possible shortening.

1/ In the Objective sections of both the Abstract and Introduction, the manuscript alternates between “refine the 32-item CPHQ for cross-domain use,” “further improve the CPHQ-instrument,” and broader descriptions of exploring stakeholder perspectives. The manuscript would thus benefit from stating one stable objective formulation and using that exact wording throughout the manuscript to keep the design, analysis, and results aligned.
Thank you for pointing out that we should use one stable formulation to keep everything aligned. We changed the wording in our manuscript to “refine” throughout the entire manuscript.

2/ In the Abstract (“We conducted focus group discussions (9 FGDs; n=76)”) and in the Methods/Table 1, where ten focus groups are listed (“Focus group 1” through “Focus group 10”), it seems that there is a mismatch in the reported number of FGDs. The manuscript would benefit from aligning the number of focus groups consistently across the Abstract, Methods, Results, and tables, and clarifying whether one group was excluded from a specific stage of analysis.
Thank you for addressing this mismatch. We organized ten FGDs but for one of the patient FGDs only one of the patients who agreed to participate showed up. Therefore, we conducted nine FGDs and one interview. We have explained this more in depth in our manuscript to enhance clarity and transparency.

Line 46 in the abstract now states: “We conducted nine focus group discussions (9 (FGDs) and one interview;( n=76) with stakeholders from healthcare, welfare, and policy domains, research, patients and (lower SEP) citizens.”. IN the methods section line 160 in now states: “For one of the FGDs with patients, only one of the patients who agreed to participate showed up. This FGD was therefore approached as an interview using similar questions as the FGDs.” And line 237-238 now states: “As one FGD with patient turned out as an interview, we combined this data with the other patient FGD. This means all patients were analyzed together.”.

3/ In the FGD method subsection, the manuscript states that “Participants in each focus group were divided into groups of no more than five persons,” however no rationale is provided for limiting the discussion groups to five persons, whereas focus group methodology is often described as favoring group sizes closer to 8–12 participants (e.g., Vaughn, 96, Sage Pub), with possible consequences for the breadth of interaction and diversity of viewpoints; The manuscript would benefit from specifying the methodological referential used to justify this choice, and clarifying whether these were considered full focus groups or smaller breakout groups within a larger focus-group session.

The participants focus group discussion groups were divided into smaller sub-groups during part of the focus group discussion. We started and closed with a full group discussion. We limited the number of participants in these sub-groups to a maximum of five participants in order to be able to have sufficient time and opportunity for each individual of the group to state their opinion. Given the fact that posters with statements needed careful consideration, we ensured that every member’s opinion was heard. When a participant was silent, a discussion leader would give that member the floor. Before and after the discussions We are convinced that with the methodology of using smaller sub-groups and starting and concluding in a bigger full group discussion we used all opportunity to capture participants’ opinions and ensured substantiation in the smaller sub-groups while also ensuring that the diversity of opinions was still taken into account in the full focus group.

In the method section of the manuscript we have clarified our choice by:
1) clarifying that we started and closed with a full focus group discussion,
2) by adding clarification about the starting discussion with all participants in line 180-184: “Full group starting discussion: First, the concept of broad health was defined and communicated to participants as: “The extent to which one is capable to adapt and to thrive given one’s physical, mental, social and contextual opportunities” (15). Second, the discussion addressed topics as: what does health consist of, what do we need to capture when measuring health and preconditions of a measuring instrument.“
3) adding two sentences to line 187-191: “Participants in each FGD were divided into approximately equal sub-groups of no more than five persons per sub-group. Each group was accompanied by a researcher that guided the process. The group size was limited to a maximum 5 participants in order to have sufficient time and opportunity for each individual of the group to state their opinion. Silent participants were encouraged to speak up by the researcher.” and
4) by explicating closure with a full group discussion in line 209-212: “Full group closing discussion: After both rounds, the FGD was closed with a discussion among all participants of the FGD to ensure full diversity of opinions was taken into account. The short discussions were mostly about the most striking findings and some additional discussion points related to the preferred number of items and answer method of a measurement instrument.”.

4/ In the Analysis of the FGDs subsection, the thresholds “>50%” and “≤15%” are presented as decision rules for “retain,” “adjust,” and “omit,” but their applicability to this specific focus-group item-refinement setting remains unclear since the rules are borrowed from another consensus context; The manuscript would benefit from adding one concise explanation of why these thresholds were considered suitable for this study design and dataset.

The CPHQ2.0 is intended for use for diverse populations and each opinion during the focus groups was weighed equally. Therefore, you could refer to the use of >50% as a democratic process to ensure a majority decision rather than a plurality. In addition, we used the ≤15% cutoff to identify significant amount of people with a contrasting opinion as the 50% group. We were of the opinion that even if there would be a majority of more than 50%, if there would be a large group opposing that opinion, the reasoning would need to be discussed in the core group rather than immediately make a decision.

In the manuscript we have clarified this in the method section by adding two sentences in line 232-235: ”The >50% cutoff were used to reflect equal weighing of each opinion and a democratic process of majority decision rather than plurality. The ≤15% cutoff was used to identify contrasting opinions compared to the 50% group, in order to investigate, discuss and form a substantiated opinion.”

5/ In the Introduction and Discussion, the manuscript alternates between the terms “broad health,” “Positive Health,” “Context-sensitive Positive Health Questionnaire,” and “Capability Approach,” without always specifying whether these terms refer to the same construct, a framework, or a measurement target; The manuscript would benefit from defining these terms once and using them with stricter consistency throughout.

Wishing you every success in the subsequent editorial stages !

We appreciate the reviewer’s request for stricter consistency to enhance clarity and the support during the editorial stages.

We have reread our manuscript clarified certain terms. In the introduction we adjusted the following:

1)Positive health is defined in the first paragraph of the introduction. Thereafter the Capability approach is defined as a dynamic framework, which does not only look at the outcomes (which is done in Positive Health), but also places health in context of the people’s capability and social and environmental resources. We have indicated this a bit more clearly by adding a couple of words in line 74-75: ”The Capability Approach …..differs from Positive Health as it is …”

2)In the second paragraph in line 85-86 we have added a sentence to clarify that we use the term ‘broad health’ to refer to the broad conceptualization of health which includes the concept of positive health and the capability approach framework.

3)In the third paragraph in line 90-91, three words are added to indicate the section refers to a Positive health questionnaire.

4)The fourth paragraph starts with the explanation of the Context-sensitive Positive Health Questionnaire (CPHQ). In the first sentence in line 104-107 it is explained that the questionnaire is based on both Positive Health and the Capability Approach.

By explicating specific terms in the introduction, we believe that the terms used in the discussion are now clear. We only omitted the term well-being in line 517.

By improving the clarity on the previous paragraphs, we believe this is now consistently defined.
