# Supplementary material for: Stakeholder and end-user perspectives to refine the Context-sensitive Positive Health Questionnaire (CPHQ) to measure broad health
Source: PLoS One. 2026 Aug 24;21(8):e0330014. doi: 10.1371/journal.pone.0330014 (PMC13502646; doi:10.1371/journal.pone.0330014)
Supplement: Supplementary file 9 [file pone.0330014.s010.docx]

Rebuttal letter: PONE-D-25-39699
Stakeholder and end-user perspectives to improve the Context-sensitive Positive Health Questionnaire (CPHQ) to measure broad health

Dear editors and reviewers,

We would like to thank you for your constructive feedback on our manuscript. We appreciate the time and effort that you took to review our manuscript. We have carefully considered all your comments and revised our manuscript accordingly. We believe that these revisions improved the clarity and quality of our manuscript. Below, we provide a point-by-point response to each comment. Reviewer and editor comments are in black font color followed by our response and a description of our changes in blue. We hope that the revised version meets your expectations and we look forward to your response.

On behalf of all authors,
Cheryl Roumen and Mirte Boelens

Journal Requirements:

1. If the reviewer comments include a recommendation to cite specific previously published works, please review and evaluate these publications to determine whether they are relevant and should be cited. There is no requirement to cite these works unless the editor has indicated otherwise. 

2. Please review your reference list to ensure that it is complete and correct. If you have cited papers that have been retracted, please include the rationale for doing so in the manuscript text, or remove these references and replace them with relevant current references. Any changes to the reference list should be mentioned in the rebuttal letter that accompanies your revised manuscript. If you need to cite a retracted article, indicate the article’s retracted status in the References list and also include a citation and full reference for the retraction notice.

Reviewers' comments:

**Comments to the Author**

1. If the authors have adequately addressed your comments raised in a previous round of review and you feel that this manuscript is now acceptable for publication, you may indicate that here to bypass the “Comments to the Author” section, enter your conflict of interest statement in the “Confidential to Editor” section, and submit your "Accept" recommendation.

Reviewer #2: All comments have been addressed

Reviewer #3: All comments have been addressed

2. Is the manuscript technically sound, and do the data support the conclusions?

The manuscript must describe a technically sound piece of scientific research with data that supports the conclusions. Experiments must have been conducted rigorously, with appropriate controls, replication, and sample sizes. The conclusions must be drawn appropriately based on the data presented.

Reviewer #2: Partly

Reviewer #3: Yes

3. Has the statistical analysis been performed appropriately and rigorously?

Reviewer #2: Yes

Reviewer #3: Yes

4. Have the authors made all data underlying the findings in their manuscript fully available?

The [PLOS Data policy](https://eur03.safelinks.protection.outlook.com/?url=http%3A%2F%2Ftrack.editorialmanager.com%2FCL0%2Fhttp%3A%252F%252Fwww.plosone.org%252Fstatic%252Fpolicies.action%2523sharing%2F1%2F010f019e4871193f-158b3223-ea5e-4ddc-acaa-efd6c7b11355-000000%2Fw4p_EtD6K0uTV4PgxXoEU2hE3DhcsY1ytsmgg_UMSiA%3D258&data=05%7C02%7Cm.boelens%40lumc.nl%7C43aec996023e41ae7fea08deb6e3bab4%7Cc4048c4fdd544cbd80495457aacd2fb8%7C0%7C0%7C639149286380786472%7CUnknown%7CTWFpbGZsb3d8eyJFbXB0eU1hcGkiOnRydWUsIlYiOiIwLjAuMDAwMCIsIlAiOiJXaW4zMiIsIkFOIjoiTWFpbCIsIldUIjoyfQ%3D%3D%7C0%7C%7C%7C&sdata=w8GiVjF9bAyT8vaJEchjCu7%2BHxGLnktpNIRYObut638%3D&reserved=0) requires authors to make all data underlying the findings described in their manuscript fully available without restriction, with rare exception (please refer to the Data Availability Statement in the manuscript PDF file). The data should be provided as part of the manuscript or its supporting information, or deposited to a public repository. For example, in addition to summary statistics, the data points behind means, medians and variance measures should be available. If there are restrictions on publicly sharing data—e.g. participant privacy or use of data from a third party—those must be specified.

Reviewer #2: No

Reviewer #3: Yes

5. Is the manuscript presented in an intelligible fashion and written in standard English?

PLOS ONE does not copyedit accepted manuscripts, so the language in submitted articles must be clear, correct, and unambiguous. Any typographical or grammatical errors should be corrected at revision, so please note any specific errors here.

Reviewer #2: Yes

Reviewer #3: Yes

6. Review Comments to the Author

Reviewer #2:
l46 The 'n=76' is misplaced and somewhat confusing as it occur after 'one interview'. If it refers to the number of participants it should go after 'stakeholders'?
We adjusted this accordingly.

ll53 For the 'At the same time, views diverged' lacks clarity as to what happened in these results. Due to a lack of consensus were these items excluded?
Thank you for pointing out that our phrasing was unclear. We have clarified this sentence in the abstract by explicitly stating that the differing views did not automatically lead to omission of the items but that these final items were based on quantitative scoring and qualitative feedback and the core group discussions.

l87 Can you specify more clearly what is meant by 'no adequately validated questionnaires' as this is the crux of reasoning for why this study occured. What were the issues with these questionnaires? How does this research seek to address them?
Thank you for highlighting this. Existing broad-health questionnaires either lack sufficient psychometric validation, were developed primarily as dialogue tools rather than measurement instruments and do not adequately include contextual factors such as social, political or environmental influences on health. To illustrate, one of the first instruments was the 42-item Positive Health questionnaire which lacks comprehensibility and discriminant validity(1, 2). It was also not developed as a measuring instrument but as a dialogue tool. A shorter version, the 17-item Positive Health questionnaire showed improved psychometric properties but the validity and reliability of the constructs underlying the questionnaire has not yet sufficiently been shown. Moreover, the IPH-17 is primarily focused on perceived health whereas contextual factors are also important(2). Our study aims to address these limitations by refining a questionnaire that is theoretically grounded in both Positive Health and the Capability Approach and includes contextual determinants of health. This is explained in the paragraph below.

We agree that this was not entirely clear in our introduction. We reformulated some sentences in our introduction and feel that it is now sufficiently clear.

l93 It would be helpful to specify why it was not 'suitable'.
We have specified this accordingly. It was not suitable as it lacks comprehensibility and also in discriminant validity.

l143 Specify who the 'experts' were.
We agree that this is not entirely clear as we explain this later in the manuscript. We added a description about the experts. The experts include the core group and expert from the Dutch National Positive Health research Network. This network covers a broad range of researchers and we now specify this in the manuscript.

We specified this at the start of the method section in l138-147*: The core group (MS, JK, MB, CR, EB, JD) consisted of six project members with diverse research backgrounds: a researcher with a background in psychology and statistics, a researcher with a background in population health, a researcher in the field of broad health concepts, a researcher in the field of health behavior, health promotion and public health, a researcher with a background in nutritional sciences and public health, and a researcher with a background in participatory research and shared decision-making.*

*The Dutch National Positive Health research Network includes researchers from several Dutch universities, hospitals, the National Institute of Public Health and the Environment, the Institute of Positive Health, and VILANS a knowledge organization for care and support*

l154-158 Suggest list these numerically for structure and clarity, as they are currently a long wordy read.
Thank you for this suggestion. We agree that the description of the stakeholder groups was lengthy and difficult to read. We have revised this by presenting the stakeholders in a more structured format. We also corrected a minor error in table 1.

l195 What does 'memo' refer to? Does this mean the CPHQ lists of items?
We agree that this may not entirely clear. A memo refers to a sticky note on which participants of the FGDs wrote their qualitative feedback concerning the individual CPHQ items. We changed the word in the manuscript to sticky notes.

l207 What is Appendix S3 referring to?
It refers to the example prompts (topic list) used for the FGDs. We rephrased the sentence for to improve clarity and readability.

l219 As above - what are the memos referring to?
A memo refers to a sticky note. We changed the word in the manuscript to sticky notes. The sticky notes included qualitative feedback discussed by the focus group members about individual CPHQ items. We specified this in the manuscript (l255 and l258).

l217 Clarify how the themes were determined?
Thank you for addressing tat this important aspect is not clear. We have clarified the analytical approach used to identify the themes. The themes were explored through deductive and inductive qualitative content analysis off both the focus group transcripts and the participant feedback on the sticky notes. The analysis focused specifically on feedback regarding the questionnaire items (e.g., wording, overlap and missing content) rather than on generating a broader theory of health.

It now states (L 243-248): *FGDs were transcribed ad verbatim and analyzed independently by two researchers (CR and MB). Qualitative feedback on the sticky notes and the transcripts were analyzed deductively on the following themes: formulation, overlap, redundancy, importance, new suggestions, duration and length of the questionnaire and inductively using content analysis. Analysis of the feedback on sticky notes for was done using an adapted consensus method inspired by the COMET handbook and Blazeby et al.(3, 4).*

l222-232 This decision-making process seems unnecessarily complex and confusing, especially considering it was done per FDG and these groups ranged in size from 6 to 14 participants. Or were all groups combined and then these metrics were calculated?
Thank you for this comment. We agree that the decision-making procedure was not sufficiently clear. Quantitative scoring was first calculated separately for each focus group. Subsequently, the results (e.g,, omit, adjust, retain) were aggregated to determine whether overall consensus had been reached. Items for which overall consensus was reached were retained, adjusted or omitted accordingly. For items where no overall consensus was reached, the core group reviewed the quantitative results together with the qualitative feedback from the transcripts and and the sticky notes and the golden ticket prioritization to reach a final decision. This procedure enabled us to identify variations between focus groups. We have clarified this in the manuscript and thoroughly rewrote this section, see l238-290*:* ***Analysis of the FGDs*** *Descriptive analyses were performed for educational level, gender and work experience using IBM SPSS statistics for Windows, version 29.0 (International Business Machines Corporation, Armonk, New York).*

*FGDs were transcribed ad verbatim and analyzed independently by two researchers (CR and MB). Qualitative feedback on the sticky notes and the transcripts were analyzed deductively on the following themes: formulation, overlap, redundancy, importance, new suggestions, duration and length of the questionnaire and inductively using content analysis. Analysis of the feedback on sticky notes for was done using an adapted consensus method inspired by the COMET handbook and Blazeby et al.(3, 4).*

*For each FGD all items were scored quantitatively by counting the colored stickers scored as 1 for green, 2 for yellow and 3 for orange. Number of golden tickets was scored per item. Decision rules were applied per item separately for each FGD.* *An item was retained when >50% scored 1 (green) and ≤15% 3 (orange), adjusted when >50% scored 2 (yellow) and ≤15% 1 (green), and omitted when >50% scored 3 (orange) and ≤15% 1 (green).* *The >50% cutoffs were used to reflect equal weighing of each opinion and a democratic process of majority decision rather than plurality. The ≤15% cutoff was used to identify contrasting opinions compared to the 50% group, in order to investigate, discuss and form a substantiated opinion.

If an item score fell outside these predefined categories, the quantitative result was considered inconclusive and the item was subsequently discussed by the core group together using the quantitative results together with the qualitative input from the sticky notes and transcripts. When the quantitative results were close to one of the predefined categories (retain, adjust or omit), this was taken into consideration during discussion, but did not automatically determine the final decision. Final decisions were reached through consensus within the core group.*  *As one FGD with patients turned out as an interview, we combined this data with the other patient FGD data. This means all patients were analyzed together.

The resulting scores (retain, adjust and omit) were subsequently compared across all FGDs to determine whether consensus between FGDs had been reached. Consensus was defined as all FGDs resulting in the same quantitative decision (retain, adjust or omit) for a given item. In these cases, the consensus decision was followed by the core group. When decisions differed between FGDs, the item was discussed by the core group using the quantitative results, qualitative feedback from the transcripts and sticky notes and the golden ticket prioritization.*

*The goal of the qualitative feedback was not to examine emerging themes but rather to examine which aspects of the individual CPHQ items required adaptation or adjustment or removal. Therefore, all qualitative feedback was grouped all per individual CPHQ item across FGDs. Feedback was summarized by examining the frequency of recurring comments, for example that an item was difficult to understand or should be reformulated.*
*Items without consensus between FGDs were discussed by the core group. Consensus was achieved through structured discussion until agreement was reached. For each item, a different core group member initiated the discussion by stating their preferred decision and rationale. Subsequently, the remaining members shared their views in a rotating order. At least four out of the six core group members had to agree on a decision to retain, adjust or omit, merge an item.. This structured discussion format was deliberately applied to ensure that all core group members had equal opportunity to contribute and to mitigate potential dominance effects or power imbalances within the group. CR and MB facilitated the discussion.*

*Items classified as ‘adjust’ were reformulated based on the qualitative feedback. This could also include merging items together.

Finally items that were retained, adjusted and sometimes merged were further refined to improve readability, consistency and comprehensibility across the questionnaire.*

*Step 1 led to a revised CPHQ with a reduced set of items.*

l322 What type of qualitative analysis was used? it is problematic that this is not clarified throughout the article. Were deductive or inductive approaches used? Who in the team has qualitative experience? Given the background of the core team, there seems to be significant potential for bias to occur- as noted by the authors.
First of all, we agree that the qualitative approach should have been described more explicitly. We have clarified that we applied a deductive and inductive content analysis. We deductively explored the following themes formulation, overlap, redundancy, importance, new suggestions, duration and length of the questionnaire. Our manuscript now states (l243-248): *FGDs were transcribed ad verbatim and analyzed independently by two researchers (CR and MB). Qualitative feedback on the sticky notes and the transcripts were analyzed deductively on the following themes: formulation, overlap, redundancy, importance, new suggestions, duration and length of the questionnaire and inductively using content analysis. Analysis of the feedback on sticky notes for was done using an adapted consensus method inspired by the COMET handbook and Blazeby et al.(3, 4).*

We also expanded the researcher positionality/reflexivity section by adding which core team member has experience with qualitative research. We also agree that there is a potential for bias and we tried to reduce this by working in multidisciplinary team that included differing views towards Positive Health, by independently coding by two researchers, by discussions until consensus was reached, the member check and by the involvement of multiple different stakeholder groups. This is already mentioned in the researcher positionality/reflexivity however, we now included this potential source of bias in our discission of the limitations, see l586-591: *Another potential limitation is the risk of confirmation bias because of the background of the core team members and by consultation of the Dutch National Positive Health research Network. To limit this potential bias, we worked in an interdisciplinary team with differing views towards Positive Health, data were independently by two researchers, by discussion until consensus was reached, the member check (although with a low response rate) and the involvement of multiple different stakeholder groups.*

Table 2 - I appreciate why statistically you have used a Median and IQR for Age, but providing a Mean and Age Range would be clearer for the reader.
We thank you for drawing attention to this. However, using the median and IQR for age is, in this case, statistically more sound compared to using the mean and standard deviation. Therefore, we decided to show the median and IQR.

l331 - missing the word 'see' before Appendix S5
We adjusted this accordingly.

l334 - typo for 'table' should be capitalised.
We adjusted this accordingly.

Table 4 is very difficult to read. Suggest reformat in Landscape not Portrait.
We adjusted this accordingly.

Table 4 - Item 3 (original) is very different to the Revision wording, to the extent it isn't measuring the same thing. What someone values is very different to what is important.
Thank you for this observation. During the FGDs, participants indicated that the wording of the original item 3 was perceived as abstract and difficult to understand and that it was not sufficiently inclusive. Participants mentioned that it was not sufficiently inclusive for people who do not work or study (e.g., people with a disease, who are jobless or who are retired).
The revised wording was therefore chosen for improved comprehensibility, particularly for participants with lower literacy levels and for inclusivity. Throughout the discussions in the FGDs participants seemed to interpret both formulations as referring to personally meaningful activities. We acknowledge that there may be subtle differences between what someone values and what someone finds important, even though in our focus group discussions this was interpreted similarly. We added this consideration to the discussion. On an additional note, during the revision process, we continued with our research to the CPHQ and validated the refined questionnaire. The item did not make in into the final 20-item validated questionnaire(5).

We added this to the limitation section (l591-601): *Some items were revised to be more understandable, comprehensible and inclusive. For example, original item “I am able to participate in activities that I value in my daily life (work, study, etc)” that changed to “I can do what I think is important” and original item “I can move easily, such as climbing stairs, walking or cycling” to “I can move” we omitted the examples. Examples such as work or study may lead to people feeling excluded for example because they cannot study or work but still do things they find important such as volunteer work. We acknowledge that there may be subtle differences between what someone values and what someone finds important but during the FGDs this was interpreted similarly by the participants. Similarly, examples about whether people can move such as climbing stairs, walking or cycling are not sufficiently inclusive for people who are in a wheelchair. Furthermore, during the FGDs participants mentioned that this item should measure mobility, i.e., are you able to go where you want to be?*

Table 4 - Item 7 Revision seems highly problematic. Unless you have a severe spinal cord injury, everyone can move. The original item that denoted 'easily' was a much better descriptor of what the item seems to be trying to measure.
Thank you again for this thoughtful comment. We agree that the revised item may appear broader than the original formulation. During the FGDs participants consistently requested shorter items and the omission of examples. Therefore, we removed the examples and the item was shortened. Furthermore, qualitative feedback was to assess perceived mobility, which with the previous example would not be possible if in a wheelchair for example, rather than the mere ability to move (using you legs). They stakeholder preferred the item to state something about there mobility, i.e., that they could get to the place they wanted to be whether that be by walking a wheelchair or using other ways of transport should be left open. The revised wording was therefore chosen to indicate mobility, for improved comprehensibility, particularly for participants with lower literacy levels and for inclusivity. On an additional note, during the revision process, we continued with our research to the CPHQ and validated the refined questionnaire. The item “I can move” was categorized under the domain “mobility” together with the item “I can be active in my community”(5). Therefore, we feel that it measure what it should measure according to the stakeholder input.

We added this to the limitation section (l591-601): *Some items were revised to be more understandable, comprehensible and inclusive. For example, original item “I am able to participate in activities that I value in my daily life (work, study, etc)” that changed to “I can do what I think is important” and original item “I can move easily, such as climbing stairs, walking or cycling” to “I can move” we omitted the examples. Examples such as work or study may lead to people feeling excluded for example because they cannot study or work but still do things they find important such as volunteer work. We acknowledge that there may be subtle differences between what someone values and what someone finds important but during the FGDs this was interpreted similarly by the participants. Similarly, examples about whether people can move such as climbing stairs, walking or cycling are not sufficiently inclusive for people who are in a wheelchair. Furthermore, during the FGDs participants mentioned that this item should measure mobility, i.e., are you able to go where you want to be?*


l345 It would be helpful to specify who the experts were at this stage.
We agree that this is not entirely clear as we explain this later in the manuscript. We added a description about the experts. The experts include the core group and expert from the Dutch National Positive Health research Network. This network covers a broad range of researchers and we now specify this in the manuscript.

We specified this at the start of the method section in l138-147: *The core group (MS, JK, MB, CR, EB, JD) consisted of six project members with diverse research backgrounds: a researcher with a background in psychology and statistics, a researcher with a background in population health, a researcher in the field of broad health concepts, a researcher in the field of health behavior, health promotion and public health, a researcher with a background in nutritional sciences and public health, and a researcher with a background in participatory research and shared decision-making.*

*The Dutch National Positive Health research Network includes researchers from several Dutch universities, hospitals, the National Institute of Public Health and the Environment, the Institute of Positive Health, and VILANS a knowledge organization for care and support.*

l362 - Should read Table not table
We adjusted this accordingly.

There are some issues with the 'expert' added items. Namely: Item 2 clearly describes achievement - it is not about meaning or belonging. Item 3 is poorly worded and relates to three different things - work, volunteering and 'informal care' which will most likely to jargon to a layperson. Similarly Item 4 sounds like psychological jargon with 'boundaries' and should be rephrased for a layperson..
I also think it is problematic that the 'expert' group came in over the top of the community consultation and added their own preferred items and words in. It would have been more methodologically sound to do this prior to the community consultation, not afterwards.
Thank you for this observation. We agree that the expert-added items required further evaluation. The purpose of the expert validation was not to override stakeholder input, but rather to ensure that important theoretical constructs identified in Positive Health and the Capability Approach literature were adequately represented in the CPHQ questionnaire after stakeholder consultation.

We3 acknowledge the methodological concern regarding the timing of the expert review. Ideally, theoretical and stakeholder perspectives should be integrated iteratively throughout the development phase. In our study the, expert validation was intended as a final content validation step to identify potential gaps in construct coverage rather than to replace or supersede stakeholder preferences.

We continued our research and conducted a validation study to the refined CPHQ. Some items were exclude based on the factor analysis (item 2, item 3, and item 4) whereas for others the relevance of maintaining the items (item 1 and item 5) was confirmed. Interestingly, item 2, 3 and 4 are exactly the items that you identified as possibly problematic. These findings thus support your observation and underline the importance of empirically testing theoretically motivated additions alongside stakeholder-informed content.

l381 The comment that the 'experts' felt that the CPHQ should be based on theory hence the use of Capability theory is interesting. Surely the CPHQ is based on Positive Health theory?
Indeed, the CPHQ is based on both Positive Health and on the Capability Approach. We worded this a bit awkwardly. We agree that the CPHQ is grounded in the concept of Positive Health. However, in the development and refinement process, we explicitly also used the Capability Approach as a structuring framework to operationalize and categorize items. This was done because the Capability approach offers a more explicit theoretical lens to structure what people are actually able to do and be in context. We have now clarified this in the manuscript as well. See l435-438: *Categorization according to the Capability Approach was used to ensure the analytical framework complementing the Positive Health foundation of the CPHQ as done because experts emphasized the importance of an explicit theoretical structure in the operationalization of the questionnaire items. felt that the CPHQ questionnaire should be based on theory.*


l395 Shoud read Table not table 7
We adjusted this accordingly.

Table 7 should be for each of the final items, not only the Totals. The range of ratings for each item should also be provided.
We did a member check on the full questionnaire. We asked participants about the readability, comprehensibility and subheadings of the total questionnaire and did not ask them about all the single final items. We agree with the reviewer that we could have done this, but we decided not to because we felt that this would make our member check to long resulting in a lower response rate. We added this as a limitation in our discussion, see l517-520: *The member check was deliberately designed to assess the overall readability, comprehensibility and the subheadings f the questionnaire instead of individual items because long questionnaires tend to reduce the response rate. Therefore, the member check does may give insight in the readability and comprehensibility of the individual items.*

l403 Again should read Table not table 8
We adjusted this accordingly.

l413 It looks like 'times' is an error and should be 'items'.
Indeed, this should be items. We adjusted this accordingly.

My comments above tend to be editorial, with some minor methodological issues. Essentially this is a long article on the item (re) development phase of a scale which now needs some good quantitative research done to it. A minor (? major) issue is I think the scale is misnamed. Its not at all about the contexts within which patients life their lives and deal with it's challenges. It is, in fact, entirely without context in the items, and the capability framework is about linguistic expression of capabilities, it is not at all about context. Given these concerns though, positive health is an important, slowly emerging concept, and a good scale to measure it well is a worthy endeavour.
Thank you for this thoughtful and constructive comment. We appreciate your careful reading of our manuscript and the recognition of the importance of developing a good scale to measure broad health.

We acknowledge that context-sensitive may be interpreted in different ways. However, we respectfully differ from the view that the questionnaire is entirely without context. The CPHQ was specifically developed to include contextual factors that influence health, in line with the Capability Approach.

Within the Capability Approach, health is understood not only in terms of individual functioning but also in relation to the resources and conversion factors that shape what people are effectively able to be and do.
Accordingly, the CPHQ includes items addressing social support, exclusion, financial resources, confidence in society, safety, health literacy and participations. These factors were identified through co-creation with stakeholders, including (lower SEP) citizens and patients, in the FGDs. These items are present in the CPHQ as resources, conversion factors and even via capabilities by measuring what people are able to do or achieve within their specific contextual circumstances.

The term context-sensitive therefore refers to the explicit incorporation of these contextual influences on health throughout the questionnaire. while the items do not always directly describe specific life situations, they aim to capture the contextual conditions that influence health opportunities and outcomes.

As noted in our response to earlier comments, we subsequently conducted a psychometric validation study of the refined CPHQ. this validation study confirmed that the contextual items remained integral components of the final questionnaire, supporting the use of the term context-sensitive(5).

To improve clarity we added the following information to the introduction in l109-115 after “…factors influencing health (6)”: *Therefore, in a previous study, we developed and validated a Context-sensitive Positive Health Questionnaire (CPHQ) as a measurement instrument based on the principles of Positive Health and grounded in the Capability Approach by accounting for differences in capabilities and conversion factors influencing health(6). The term context-sensitive refers to the explicit inclusion of contextual influences on health throughout the questionnaire. while items may not always directly describe specific life situation, they aim to capture contextual conditions that influence health opportunities and outcomes.*

Reviewer #3: The authors carried out a very thorough revision and I am particularly satisfied with the final state of the manuscript.
We thank the reviewer for this compliment. We were happy with all comments previously received to improve our manuscript.

**References**

1.	Prinsen CAC, Terwee CB. Measuring positive health: for now, a bridge too far. Public Health. 2019;170:70–7.

2.	Van Vliet M, Doornenbal BM, Boerema S, van den Akker-van Marle EM. Development and psychometric evaluation of a Positive Health measurement scale: a factor analysis study based on a Dutch population. BMJ Open. 2021;11(2):e040816.

3.	Williamson PR, Altman DG, Bagley H, Barnes KL, Blazeby JM, Brookes ST, et al. The COMET Handbook: version 1.0. Trials. 2017;18(Suppl 3):280.

4.	Blazeby JM, Macefield R, Blencowe NS, Jacobs M, McNair AG, Sprangers M, et al. Core information set for oesophageal cancer surgery. Br J Surg. 2015;102(8):936–43.

5.	Dubbeldeman EM, Boelens M, Bloemen-van Gurp EJ, Dierx JAJ, Spreeuwenberg MD, Kiefte-de Jong JC. A longitudinal psychometric evaluation of a context-sensitive positive health questionnaire for measuring broad health in Dutch adults. Journal of Patient-Reported Outcomes. 2026.

6.	Doornenbal BM, van Zutphen T, Beumeler LFE, Vos RC, Derks M, Haisma H, et al. Development and validation of a Context-sensitive Positive Health Questionnaire (CPHQ): A factor analysis and multivariate regression study. Journal of Patient-Reported Outcomes. 2024;8(1):44.
