# Supplementary material for: Stakeholder and end-user perspectives to refine the Context-sensitive Positive Health Questionnaire (CPHQ) to measure broad health
Source: PLoS One. 2026 Aug 24;21(8):e0330014. doi: 10.1371/journal.pone.0330014 (PMC13502646; doi:10.1371/journal.pone.0330014)
Supplement: Peer review history

# Peer review: Decision Letter 0

**DOI:** 10.1371/journal.pone.0330014.r001

**Authors:** Vanessa Carels

15 Oct 2025

Dear Dr. Boelens,

Thank you for submitting your manuscript to PLOS ONE. After careful consideration, we feel that it has merit but does not fully meet PLOS ONE’s publication criteria as it currently stands. Therefore, we invite you to submit a revised version of the manuscript that addresses the points raised during the review process.

Please note that we have only been able to secure a single reviewer to assess your manuscript. We are issuing a decision on your manuscript at this point to prevent further delays in the evaluation of your manuscript. Please be aware that the editor who handles your revised manuscript might find it necessary to invite additional reviewers to assess this work once the revised manuscript is submitted. However, we will aim to proceed on the basis of this single review if possible.

Please submit your revised manuscript by Nov 28 2025 11:59PM. If you will need more time than this to complete your revisions, please reply to this message or contact the journal office at plosone@plos.org. When you're ready to submit your revision, log on to https://www.editorialmanager.com/pone/ and select the 'Submissions Needing Revision' folder to locate your manuscript file.

- A rebuttal letter that responds to each point raised by the academic editor and reviewer(s). You should upload this letter as a separate file labeled 'Response to Reviewers'.
- A marked-up copy of your manuscript that highlights changes made to the original version. You should upload this as a separate file labeled 'Revised Manuscript with Track Changes'.
- An unmarked version of your revised paper without tracked changes. You should upload this as a separate file labeled 'Manuscript'.

If you would like to make changes to your financial disclosure, please include your updated statement in your cover letter. Guidelines for resubmitting your figure files are available below the reviewer comments at the end of this letter.

If applicable, we recommend that you deposit your laboratory protocols in protocols.io to enhance the reproducibility of your results. Protocols.io assigns your protocol its own identifier (DOI) so that it can be cited independently in the future. For instructions see: https://journals.plos.org/plosone/s/submission-guidelines#loc-laboratory-protocols. Additionally, PLOS ONE offers an option for publishing peer-reviewed Lab Protocol articles, which describe protocols hosted on protocols.io. Read more information on sharing protocols at https://plos.org/protocols?utm_medium=editorial-email&utm_source=authorletters&utm_campaign=protocols.

We look forward to receiving your revised manuscript.

Kind regards,

Vanessa Carels

Staff Editor

PLOS ONE

Journal requirements:

When submitting your revision, we need you to address these additional requirements.

1. Please ensure that your manuscript meets PLOS ONE's style requirements, including those for file naming. The PLOS ONE style templates can be found at

https://journals.plos.org/plosone/s/file?id=wjVg/PLOSOne_formatting_sample_main_body.pdf and

https://journals.plos.org/plosone/s/file?id=ba62/PLOSOne_formatting_sample_title_authors_affiliations.pdf

2. Thank you for stating the following in the Funding Section of your manuscript:

“This work was funded by a research grant (project number: 10530012110002) from ZonMw, The Netherlands Organization for Health Research and Development(https://www.zonmw.nl/en). ZonMw has no role in the study design, data collection and analysis, decision to publish or preparation of the manuscript.”

We note that you have provided funding information that is currently declared in your Funding Statement. However, funding information should not appear in the Acknowledgments section or other areas of your manuscript. We will only publish funding information present in the Funding Statement section of the online submission form.

Please remove any funding-related text from the manuscript and let us know how you would like to update your Funding Statement. Currently, your Funding Statement reads as follows:

“his work was funded by a research grant (project number: 10530012110002) from ZonMw, The Netherlands Organization for Health Research and Development (https://www.zonmw.nl/en). ZonMw has no role in the study design, data collection and analysis, decision to publish or preparation of the manuscript.”

Please include your amended statements within your cover letter; we will change the online submission form on your behalf.

3. In the online submission form you indicate that your data is not available for proprietary reasons and have provided a contact point for accessing this data. Please note that your current contact point is a co-author on this manuscript. According to our Data Policy, the contact point must not be an author on the manuscript and must be an institutional contact, ideally not an individual. Please revise your data statement to a non-author institutional point of contact, such as a data access or ethics committee, and send this to us via return email. Please also include contact information for the third party organization, and please include the full citation of where the data can be found.

4. Please include captions for your Supporting Information files at the end of your manuscript, and update any in-text citations to match accordingly. Please see our Supporting Information guidelines for more information: http://journals.plos.org/plosone/s/supporting-information.

If the reviewer comments include a recommendation to cite specific previously published works, please review and evaluate these publications to determine whether they are relevant and should be cited. There is no requirement to cite these works unless the editor has indicated otherwise.

[Note: HTML markup is below. Please do not edit.]

Reviewers' comments:

Reviewer's Responses to Questions

**Comments to the Author**

1. Is the manuscript technically sound, and do the data support the conclusions?

Reviewer #1: Yes

**********

2. Has the statistical analysis been performed appropriately and rigorously?

Reviewer #1: N/A

**********

3. Have the authors made all data underlying the findings in their manuscript fully available?

The PLOS Data policy

Reviewer #1: Yes

**********

4. Is the manuscript presented in an intelligible fashion and written in standard English?

Reviewer #1: Yes

**********

Reviewer #1: 1. Study aim vs. design clarity

The paper oscillates between instrument refinement and qualitative exploration of item content. Tighten the aim in Abstract/Introduction and align Methods/Results accordingly (e.g., “to refine the 32-item CPHQ for cross-domain use through stakeholder focus groups, expert review, and member checking, producing a 28-item version ready for psychometric testing”).

2. Sampling strategy and reflexivity

Provide a more transparent account of purposive sampling: who identified participants, how invitations were distributed, and how diversity (domain, geography, SEP, gender) was ensured; add a brief table on recruitment sources and non-participation. Include researcher positionality/reflexivity and how it may have shaped facilitation and analysis (e.g., backgrounds of moderators, prior ties to Positive Health). This aligns with SRQR/COREQ good practice.

3. Focus group procedures and analytic rigour

You describe two “rounds” (qualitative memos; sticker voting) plus thematic analysis and an adapted COMET decision rule. Please:

Cite or append the exact decision rules and thresholds and justify the >50% / ≤15% cut-offs; explain how discordant groups were handled.

Clarify how qualitative inputs (memos/transcripts) were integrated with sticker scores to decide retain/adjust/omit/merge at item level; a worked example for one contentious item would help.

Report coder training, codebook development, intercoder agreement (if calculated) or processes to reach consensus.

Move Table 2 (themes guiding adjustments) earlier and link each theme to concrete item changes.

4. Theoretical mapping to the Capability Approach

The recategorization into resources, conversion factors, capabilities, functionings is a major conceptual move. Provide a short, a priori mapping framework (definitions + decision rules) and a few exemplars that were debated (e.g., “I feel safe where I live” vs. “I can be active in my environment”). Consider an expert agreement check for the taxonomy.

5. Language, readability, and cross-cultural validity

You note items were developed in Dutch, translated to English by a native translator, with no back-translation. For an instrument intended for cross-domain and potentially cross-national use, justify the absence of back-translation and describe plans for formal linguistic validation and cognitive debriefing in future studies. Also, elaborate on how readability for low literacy was ensured beyond the PHAROS review (e.g., Flesch scores, cognitive interviewing).

6. Member check scope and interpretation

Response rate was 33% (18/55). Discuss non-response bias and whether respondents differed from the original focus-group sample. Summarize what changed (if anything) after the member check, and justify keeping contested items (e.g., “I feel excluded” negative phrasing) beyond stating “no major concerns.” Consider a short table mapping member-check suggestions to disposition.

7. Political representation & trust items

Decisions around removing “I feel represented by politics” but retaining “confidence in society” merit deeper justification, given prior factor structure in the original CPHQ and the Capability Approach emphasis on social conversion factors. Consider keeping both for psychometric testing, or provide empirical/analytic rationale for removal beyond stakeholder preference.

8. Data availability and transparency

The current Data Availability Statement indicates restricted access due to privacy. Provide a stronger transparency plan (e.g., share de-identified sticker tallies, decision logs for each item in a public repository; provide the final 28-item instrument, Dutch + English, with response options). This aligns with PLOS ONE policy and will aid replication.

Abstract: Report key numbers succinctly (10 FGDs; n=76; final 28 items; main themes guiding edits). Avoid method jargon (“adapted COMET”) without a one-line gloss.

Methods: Specify focus-group duration per stakeholder type; clarify whether mixed-stakeholder groups were analyzed separately. Add example prompts and the “handbook” list to an SI.

Results:

Tables 3–5 are valuable. Add a column in Table 3 linking each edit to the qualitative theme and COMET outcome; indicate where “golden tickets” influenced decisions.

Report descriptive characteristics per group consistently; a CONSORT-like flow for recruitment would help readability.

Discussion:

When noting the primacy of functionings items (“I feel healthy/happy/fit”), link this more explicitly to measurement implications (risk of redundancy with global health/QoL items; need to test higher-order factor).

Expand on implications for monitoring population broad health vs. program evaluation, including sensitivity to change.

Limitations: Acknowledge possible pro-Positive-Health sample bias; discuss implications of removing politically tinged items for construct coverage.

Presentation: Fix small typos (“pronfessionals”, spacing), standardize item wording (all first-person present), and ensure the final 28-item list in Appendix 3 exactly matches Table 5 categories.

**********

what does this mean?). If published, this will include your full peer review and any attached files.

If you choose “no”, your identity will remain anonymous but your review may still be made public.

**Do you want your identity to be public for this peer review?**  For information about this choice, including consent withdrawal, please see our Privacy Policy

Reviewer #1: No

**********

[NOTE: If reviewer comments were submitted as an attachment file, they will be attached to this email and accessible via the submission site. Please log into your account, locate the manuscript record, and check for the action link "View Attachments". If this link does not appear, there are no attachment files.]

While revising your submission, please upload your figure files to the Preflight Analysis and Conversion Engine (PACE) digital diagnostic tool, https://pacev2.apexcovantage.com/. PACE helps ensure that figures meet PLOS requirements. To use PACE, you must first register as a user. Registration is free. Then, login and navigate to the UPLOAD tab, where you will find detailed instructions on how to use the tool. If you encounter any issues or have any questions when using PACE, please email PLOS at figures@plos.org. Please note that Supporting Information files do not need this step.

# Author comment: Author response to Decision Letter 1

**DOI:** 10.1371/journal.pone.0330014.r002

26 Jan 2026

Rebuttal letter: PONE-D-25-39699

Stakeholder and end-user perspectives to improve the Context-sensitive Positive Health Questionnaire (CPHQ) to measure broad health

Dear editors and reviewers,

We would like to thank you for your constructive feedback on our manuscript. We appreciate the time and effort that you took to review our manuscript. We have carefully considered all your comments and revised our manuscript accordingly. We believe that these revisions improved the clarity and quality of our manuscript. Below, we provide a point-by-point response to each comment. Reviewer and editor comments are in black font color followed by our response and a description of our changes in blue. We hope that the revised version meets your expectations and we look forward to your response.

On behalf of all authors,

Cheryl Roumen and Mirte Boelens

Journal requirements:

When submitting your revision, we need you to address these additional requirements.

1. Please ensure that your manuscript meets PLOS ONE's style requirements, including those for file naming. The PLOS ONE style templates can be found at https://journals.plos.org/plosone/s/file?id=wjVg/PLOSOne_formatting_sample_main_body.pdf and https://journals.plos.org/plosone/s/file?id=ba62/PLOSOne_formatting_sample_title_authors_affiliations.pdf

We adjusted our files accordingly.

2. Thank you for stating the following in the Funding Section of your manuscript:

“This work was funded by a research grant (project number: 10530012110002) from ZonMw, The Netherlands Organization for Health Research and Development(https://www.zonmw.nl/en). ZonMw has no role in the study design, data collection and analysis, decision to publish or preparation of the manuscript.”

We note that you have provided funding information that is currently declared in your Funding Statement. However, funding information should not appear in the Acknowledgments section or other areas of your manuscript. We will only publish funding information present in the Funding Statement section of the online submission form.

Please remove any funding-related text from the manuscript and let us know how you would like to update your Funding Statement. Currently, your Funding Statement reads as follows:

“his work was funded by a research grant (project number: 10530012110002) from ZonMw, The Netherlands Organization for Health Research and Development (https://www.zonmw.nl/en). ZonMw has no role in the study design, data collection and analysis, decision to publish or preparation of the manuscript.”

Please include your amended statements within your cover letter; we will change the online submission form on your behalf.

We have deleted funding information from the acknowledgement section and other areas of the manuscript.

In the cover letter we ensured an update of our funding information. The funding information we would like to report is as follows: “This work was funded by a research grant (project number: 10530012110002) from ZonMw, The Netherlands Organization for Health Research and Development (https://www.zonmw.nl/en). ZonMw has no role in the study design, data collection and analysis, decision to publish or preparation of the manuscript.”.

3. In the online submission form you indicate that your data is not available for proprietary reasons and have provided a contact point for accessing this data. Please note that your current contact point is a co-author on this manuscript. According to our Data Policy, the contact point must not be an author on the manuscript and must be an institutional contact, ideally not an individual. Please revise your data statement to a non-author institutional point of contact, such as a data access or ethics committee, and send this to us via return email. Please also include contact information for the third party organization, and please include the full citation of where the data can be found.

We have edited the point of contact to the institutional point of contact of the Health Campus the Hague, department of Public Health and Primary Care at the Leiden University Medical Center via: LUMC-CampusDenHaag@lumc.nl.

Furthermore, (meta)data is uploaded in a repository (https://doi.org/10.34894/9STE1Z, Ontwikkelen Meetinstrumentarium Brede Benadering Gezondheid).

4. Please include captions for your Supporting Information files at the end of your manuscript, and update any in-text citations to match accordingly. Please see our Supporting Information guidelines for more information: http://journals.plos.org/plosone/s/supporting-information.

We have added Supporting Information files at the end of our manuscript. We adjusted in-text citations accordingly.

If the reviewer comments include a recommendation to cite specific previously published works, please review and evaluate these publications to determine whether they are relevant and should be cited. There is no requirement to cite these works unless the editor has indicated otherwise.

Thank you for pointing this out. The reviewer has not recommended to cite specific previously published work.

Reviewers' comments:

Reviewer's Responses to Questions

Comments to the Author

1. Is the manuscript technically sound, and do the data support the conclusions?

The manuscript must describe a technically sound piece of scientific research with data that supports the conclusions. Experiments must have been conducted rigorously, with appropriate controls, replication, and sample sizes. The conclusions must be drawn appropriately based on the data presented.

Reviewer #1: Yes

2. Has the statistical analysis been performed appropriately and rigorously?

Reviewer #1: N/A

3. Have the authors made all data underlying the findings in their manuscript fully available?

The PLOS Data policy requires authors to make all data underlying the findings described in their manuscript fully available without restriction, with rare exception (please refer to the Data Availability Statement in the manuscript PDF file). The data should be provided as part of the manuscript or its supporting information, or deposited to a public repository. For example, in addition to summary statistics, the data points behind means, medians and variance measures should be available. If there are restrictions on publicly sharing data—e.g. participant privacy or use of data from a third party—those must be specified.

Reviewer #1: Yes

4. Is the manuscript presented in an intelligible fashion and written in standard English?

PLOS ONE does not copyedit accepted manuscripts, so the language in submitted articles must be clear, correct, and unambiguous. Any typographical or grammatical errors should be corrected at revision, so please note any specific errors here.

Reviewer #1: Yes

5. Review Comments to the Author

Please use the space provided to explain your answers to the questions above. You may also include additional comments for the author, including concerns about dual publication, research ethics, or publication ethics. (Please upload your review as an attachment if it exceeds 20,000 characters)

Reviewer #1:

1. Study aim vs. design clarity

The paper oscillates between instrument refinement and qualitative exploration of item content. Tighten the aim in Abstract/Introduction and align Methods/Results accordingly (e.g., “to refine the 32-item CPHQ for cross-domain use through stakeholder focus groups, expert review, and member checking, producing a 28-item version ready for psychometric testing ).

We have tightened our aim in the abstract/introduction and aligned this aim with our methods/results accordingly. The objective in the introduction (see line 121-128) is adjusted to: “The objective of this study is to further improve the CPHQ-instrument to make it more generally applicable and therefore accepted across different domains through focus group discussions (FGD) incorporating the perspectives of a broader group of stakeholders across healthcare, welfare and policy domains, researchers, as well as patients and citizens, including those from a lower SEP, expert review and a member check.“.

2. Sampling strategy and reflexivity

Provide a more transparent account of purposive sampling: who identified participants, how invitations were distributed, and how diversity (domain, geography, SEP, gender) was ensured; add a brief table on recruitment sources and non-participation. Include researcher positionality/reflexivity and how it may have shaped facilitation and analysis (e.g., backgrounds of moderators, prior ties to Positive Health). This aligns with SRQR/COREQ good practice.

We acknowledge the information on the sampling process was rather brief. Therefore, we have added more detailed information about the sampling and recruitment in our manuscript. See line 168-171 in the methods sections “A purposeful sampling approach was used. This meant that the participants for FGDs were recruited based on accessibility and based on a variety of professions and backgrounds and geographical areas to capture a wide range of perspectives. The distributors of the invitations were researchers who had access to the target population (see table 1).” Furthermore, we added information in a table (table 1) in the methods section, see line 172,173 and below.

Table. 1 Recruitment of focus group discussions (FGDs)

Focus group Target group Distributor(s) of invitations Invitations (n) Participants (n) Location Date (dd-mm-yyyy) Facilitators

1 Medical specialists LNvV 15 10 Den Bosch 23-01-23 CR, EB & MB

2 Regional policymakers, welfare JS, JD 33 14 Eindhoven 25-01-23 JD, MB, EB

3 Citizens JS and passive via hardcopy information letters in a primary care practice waiting room 18 + passive 8 Eindhoven 07-02-23 EB, MB

4 Citizens TvZ, PC 15 8 Leeuwarden 24-02-23 PC, TvZ, MB

5 National policymakers JK, MS, JD, MdK, CR, EB and passive via social media 28 + passive 14 Utrecht 16-03-23 EB, MB

6 Experts CR, EM, MB MdK, and passive via social media 33 + passive 8 Utrecht 01-03-23 CR, EB, MB

7 Paramedics JS, MB and passive via social media 60 + passive 6 Eindhoven

23-03-23 JS, CR, EB, MB

8 Mixed group ( policymakers and experts) EB, MB, CR, mDK 17 6 Utrecht 06-04-23 EB, MB, CR

9 Patients CR 7 1 Maastricht 16-08-23 CR, MB

10 Patients TvZ, PC 72 8 Leeuwarden

19-09-23 PC, TvZ, MB

Furthermore, we agree with the reviewer that information on researcher positionality/reflexivity is important. We added information about researcher positionality/reflexivity in the methods section, see line 287-311

“Researcher positionality/reflexivity

The research consortium consisted of researchers with diverse backgrounds. The core group (MS1, JK2, MB3, CR4, EB5, JD6) included six project members with expertise in psychology and statistics1, population health2,3, broad health concepts1,2,5, human biology and participatory design within oncology4, nutritional sciences and public health2,3,4,5,, and participatory research and shared decision-making6. All consortium members were familiar with the frameworks of Positive Health. Especially JD had expertise on the Capability Approach. While some researchers were advocates of Positive Health (EB5, JD6) as a way to capture a broader understanding of health, others (MS1, JK2, MB3 CR4) maintained a more critical stance, emphasizing conceptual clarity and empirical validity. This diversity of perspectives fostered reflexive dialogue throughout the research process.

Some consortium members (MdK, MvV) were affiliated with the Institute of Positive Health (iPH), and one member (EB) was a certified Positive Health trainer. These positions provided valuable insight and practical expertise regarding Positive Health, but could also introduce a tendency toward interpreting the questionnaire and results in alignment with the framework.

The focus group discussions were facilitated by different consortium members (CR, MB, EB, LNvV, TvZ, PMRC, JS). There were always 2 and sometimes 3 discussion leaders depending on who was most familiar with the target group.

Data were interpreted by two lead researchers (CR, MB) with complementary perspectives, and analytic decisions were discussed within the core group (MS, JK, CR, MB, EB, JD) to enhance reflexivity. Following analysis, the wider consortium and experts from the Dutch Positive Health Network reviewed and provided feedback on the questionnaire. While this consultation offered valuable expertise, it could potentially introduce confirmatory bias, favoring interpretations aligned with the Positive Health framework. Combining independent analysis, core group discussions, and external feedback helped mitigate potential bias and strengthened the credibility and trustworthiness of the findings.”.

3. Focus group procedures and analytic rigour

You describe two “rounds” (qualitative memos; sticker voting) plus thematic analysis and an adapted COMET decision rule. Please:

Cite or append the exact decision rules and thresholds and justify the >50% / ≤15% cut-offs; explain how discordant groups were handled.

The decision rules are inspired by an example given in the COMET handbook(1). The decision rules are originally used by Blazeby et al.(2) The COMET handbook describes these decision rules to retain or omit items as less stringent which reduces the likelihood of omitting items that may be important. In the COMET handbook they use a Delphi approach consisting of two rounds. They used more stringent criteria in their second round. Instead of a second round, we discussed discordant items within the core team. Discordant items were discussed as follows: First, one core team explained the opinion, doubts and consideration of a certain item, then a second core team member would do the same until all members had given their opinion. Then, the another core team member would give a suggestion and all members would respond to that in sequence. Finally, a parallel discussion took place in which all members participated until consensus was reached. To avoid bias, for different discordant questions, different core team members started the discussion.

The discussion about these items were based on the qualitative input written on the memos and the golden tickets.

We added the reference to the comet handbook by Paula Williamson (2017) and a reference to the paper by Blazeby et al (2015) in our manuscript, see line 219. We also added a more thorough explanation of our analysis in the manuscript, see line 222-253 “For each focus group all items were scored quantitatively by counting the colored stickers scored as 1 for green, 2 for yellow and 3 for orange. Number of golden tickets was scored per item. Decision rules were applied per item separately for each focus group. An item was retained when >50% scored 1 (green) and ≤15% 3 (orange), adjusted when >50% scored 2 (yellow) and ≤15% 1 (green), and omitted when >50% scored 3 (orange) and ≤15% 1 (green). If a score of an item fell outside these categories, the core group decided that it was inconclusive or proposed a suggestion if numbers were close to either keep, adjust omit.

After this, in case of consensus about an item across the focus groups (when all focus groups score the same on an item), this score (retain, adjust, omit) was followed by the core group. If the scoring of an item was inconclusive (discordant items), the core group decided on retaining, adjusting, omitting or merging items when meaning and content overlapped based on the memos with qualitative input and the golden tickets.

The goal of the qualitative feedback was not to examine emerging themes but rather to examine what aspects of the CPHQ items need adaptation or adjustment and what is perceived as useful or not useful. Therefore, we grouped all qualitative feedback from all focus groups per item. We examined how often a certain type of feedback was given for each item, e.g. “this item is complicated, consider reformulating the item”.

Discordant items (items without consensus based on quantitative scoring) were discussed by the core group. Consensus was achieved through structured discussion until agreement was reached. For each item, a different core group member initiated the discussion by stating their preference and providing a rationale. Subsequently, the other members shared their view

Attachment Submitted filename: Response to reviewers.docx

# Peer review: Decision Letter 1

**DOI:** 10.1371/journal.pone.0330014.r003

**Authors:** Katherine Demi Kokkinias

19 Mar 2026

Dear Dr. Boelens,

Thank you for submitting your manuscript to PLOS ONE. After careful consideration, we feel that it has merit but does not fully meet PLOS ONE’s publication criteria as it currently stands. Therefore, we invite you to submit a revised version of the manuscript that addresses the points raised during the review process.

==============================

The manuscript has been evaluated by two reviewers, and their comments are available below.

While many of the concerns have been addressed, the reviewers have several remaining concerns. They note that there are ten focus groups listed in table 1 but nine focus groups described in the text. They would like justification for the focus group size. They also recommend defining terminology and suggest points that could be clarified for the reader.

Could you please carefully revise the manuscript to address all comments raised?

==============================

Please submit your revised manuscript by May 03 2026 11:59PM. If you will need more time than this to complete your revisions, please reply to this message or contact the journal office at plosone@plos.org. When you're ready to submit your revision, log on to https://www.editorialmanager.com/pone/ and select the 'Submissions Needing Revision' folder to locate your manuscript file.

- A letter that responds to each point raised by the academic editor and reviewer(s). You should upload this letter as a separate file labeled 'Response to Reviewers'.
- A marked-up copy of your manuscript that highlights changes made to the original version. You should upload this as a separate file labeled 'Revised Manuscript with Track Changes'.
- An unmarked version of your revised paper without tracked changes. You should upload this as a separate file labeled 'Manuscript'.

If you would like to make changes to your financial disclosure, please include your updated statement in your cover letter. Guidelines for resubmitting your figure files are available below the reviewer comments at the end of this letter.

If applicable, we recommend that you deposit your laboratory protocols in protocols.io to enhance the reproducibility of your results. Protocols.io assigns your protocol its own identifier (DOI) so that it can be cited independently in the future. For instructions see: https://journals.plos.org/plosone/s/submission-guidelines#loc-laboratory-protocols. Additionally, PLOS ONE offers an option for publishing peer-reviewed Lab Protocol articles, which describe protocols hosted on protocols.io. Read more information on sharing protocols at https://plos.org/protocols?utm_medium=editorial-email&utm_source=authorletters&utm_campaign=protocols.

We look forward to receiving your revised manuscript.

Kind regards,

Katherine Demi Kokkinias, Ph.D.

Staff Editor

PLOS One

**Journal Requirements:**

If the reviewer comments include a recommendation to cite specific previously published works, please review and evaluate these publications to determine whether they are relevant and should be cited. There is no requirement to cite these works unless the editor has indicated otherwise.

[Note: HTML markup is below. Please do not edit.]

Reviewers' comments:

Reviewer's Responses to Questions

**Comments to the Author**

Reviewer #2: All comments have been addressed

Reviewer #3: All comments have been addressed

**********

2. Is the manuscript technically sound, and do the data support the conclusions?

Reviewer #2: Yes

Reviewer #3: Yes

**********

3. Has the statistical analysis been performed appropriately and rigorously?

Reviewer #2: Yes

Reviewer #3: Yes

**********

4. Have the authors made all data underlying the findings in their manuscript fully available?

The PLOS Data policy

Reviewer #2: Yes

Reviewer #3: No

**********

5. Is the manuscript presented in an intelligible fashion and written in standard English?

Reviewer #2: Yes

Reviewer #3: Yes

**********

Reviewer #2: My review comments are based on Revision 1 which includes multiple changes made in response to Reviewer 1's feedback. These changes have been substantively met, and no further changes are recommended. As noted by the authors, a well-designed patient-reported outcome measure is an important step in supporting the potential utility of positive health perspectives. The continued development of the CPHQ is an important step in this direction. Future research from independent research groups to clarify the reliability and validity of this measure will be an important outcome that this paper supports.

Reviewer #3: Thank you for the opportunity to evaluate this manuscript. In this work, the authors aimed to refine the 32-item Context-sensitive Positive Health Questionnaire (CPHQ) for broader use across healthcare, welfare, and policy domains. Starting from the previously developed CPHQ, they conducted focus group discussions with stakeholders from healthcare, welfare, policy, and research, as well as with patients and citizens, including individuals with lower socioeconomic position. The study combined qualitative feedback and structured item scoring during the focus groups. Participants commented on the relevance, clarity, and formulation of questionnaire items, rated whether items should be retained, adjusted, or omitted, and identified particularly important items. Based on this process, the authors omitted, merged, reformulated, and added items, resulting in a revised set of 28 items. After the focus groups, the revised questionnaire was further examined through expert validation and a member check with a subgroup of original participants. The expert review contributed to additional refinements in item wording and supported the inclusion of some items that had been reconsidered as important for capturing broad health. The final set of items was then categorized according to the Capability Approach into resources, conversion factors, capabilities, and functionings. The final result of the study is a revised 28-item CPHQ, intended as a theory-informed and stakeholder-informed instrument to measure broad health, with future research needed for further validation and possible shortening.

1/ In the Objective sections of both the Abstract and Introduction, the manuscript alternates between “refine the 32-item CPHQ for cross-domain use,” “further improve the CPHQ-instrument,” and broader descriptions of exploring stakeholder perspectives. The manuscript would thus benefit from stating one stable objective formulation and using that exact wording throughout the manuscript to keep the design, analysis, and results aligned.

2/ In the Abstract (“We conducted focus group discussions (9 FGDs; n=76)”) and in the Methods/Table 1, where ten focus groups are listed (“Focus group 1” through “Focus group 10”), it seems that there is a mismatch in the reported number of FGDs. The manuscript would benefit from aligning the number of focus groups consistently across the Abstract, Methods, Results, and tables, and clarifying whether one group was excluded from a specific stage of analysis.

3/ In the FGD method subsection, the manuscript states that “Participants in each focus group were divided into groups of no more than five persons,” however no rationale is provided for limiting the discussion groups to five persons, whereas focus group methodology is often described as favoring group sizes closer to 8–12 participants (e.g., Vaughn, 96, Sage Pub), with possible consequences for the breadth of interaction and diversity of viewpoints; The manuscript would benefit from specifying the methodological referential used to justify this choice, and clarifying whether these were considered full focus groups or smaller breakout groups within a larger focus-group session.

4/ In the Analysis of the FGDs subsection, the thresholds “>50%” and “≤15%” are presented as decision rules for “retain,” “adjust,” and “omit,” but their applicability to this specific focus-group item-refinement setting remains unclear since the rules are borrowed from another consensus context; The manuscript would benefit from adding one concise explanation of why these thresholds were considered suitable for this study design and dataset.

5/ In the Introduction and Discussion, the manuscript alternates between the terms “broad health,” “Positive Health,” “Context-sensitive Positive Health Questionnaire,” and “Capability Approach,” without always specifying whether these terms refer to the same construct, a framework, or a measurement target; The manuscript would benefit from defining these terms once and using them with stricter consistency throughout.

Wishing you every success in the subsequent editorial stages !

**********

what does this mean?). If published, this will include your full peer review and any attached files.

If you choose “no”, your identity will remain anonymous but your review may still be made public.

**Do you want your identity to be public for this peer review?**  For information about this choice, including consent withdrawal, please see our Privacy Policy

Reviewer #2: No

Reviewer #3: **Yes:** Arthur Trognon

**********

[NOTE: If reviewer comments were submitted as an attachment file, they will be attached to this email and accessible via the submission site. Please log into your account, locate the manuscript record, and check for the action link "View Attachments". If this link does not appear, there are no attachment files.]

To ensure your figures meet our technical requirements, please review our figure guidelines: https://journals.plos.org/plosone/s/figures

You may also use PLOS’s free figure tool, NAAS, to help you prepare publication quality figures: https://journals.plos.org/plosone/s/figures#loc-tools-for-figure-preparation.

NAAS will assess whether your figures meet our technical requirements by comparing each figure against our figure specifications.

# Author comment: Author response to Decision Letter 2

**DOI:** 10.1371/journal.pone.0330014.r004

28 Apr 2026

Rebuttal letter: PONE-D-25-39699

Stakeholder and end-user perspectives to improve the Context-sensitive Positive Health Questionnaire (CPHQ) to measure broad health

Dear editors and reviewers,

We would like to thank you for your constructive feedback on our manuscript. We appreciate the time and effort that you took to review our manuscript. We have carefully considered all your comments and revised our manuscript accordingly. We believe that these revisions improved the clarity and quality of our manuscript. Below, we provide a point-by-point response to each comment. Reviewer and editor comments are in black font color followed by our response and a description of our changes in blue. We hope that the revised version meets your expectations and we look forward to your response.

On behalf of all authors,

Cheryl Roumen and Mirte Boelens

Comments to the Author

1. If the authors have adequately addressed your comments raised in a previous round of review and you feel that this manuscript is now acceptable for publication, you may indicate that here to bypass the “Comments to the Author” section, enter your conflict of interest statement in the “Confidential to Editor” section, and submit your "Accept" recommendation.

Reviewer #2: All comments have been addressed

Reviewer #3: All comments have been addressed

________________________________________

2. Is the manuscript technically sound, and do the data support the conclusions?

The manuscript must describe a technically sound piece of scientific research with data that supports the conclusions. Experiments must have been conducted rigorously, with appropriate controls, replication, and sample sizes. The conclusions must be drawn appropriately based on the data presented.

Reviewer #2: Yes

Reviewer #3: Yes

________________________________________

3. Has the statistical analysis been performed appropriately and rigorously?

Reviewer #2: Yes

Reviewer #3: Yes

________________________________________

4. Have the authors made all data underlying the findings in their manuscript fully available?

The PLOS Data policy requires authors to make all data underlying the findings described in their manuscript fully available without restriction, with rare exception (please refer to the Data Availability Statement in the manuscript PDF file). The data should be provided as part of the manuscript or its supporting information, or deposited to a public repository. For example, in addition to summary statistics, the data points behind means, medians and variance measures should be available. If there are restrictions on publicly sharing data—e.g. participant privacy or use of data from a third party—those must be specified.

Reviewer #2: Yes

Reviewer #3: No

________________________________________

5. Is the manuscript presented in an intelligible fashion and written in standard English?

PLOS ONE does not copyedit accepted manuscripts, so the language in submitted articles must be clear, correct, and unambiguous. Any typographical or grammatical errors should be corrected at revision, so please note any specific errors here.

Reviewer #2: Yes

Reviewer #3: Yes

________________________________________

6. Review Comments to the Author

Please use the space provided to explain your answers to the questions above. You may also include additional comments for the author, including concerns about dual publication, research ethics, or publication ethics. (Please upload your review as an attachment if it exceeds 20,000 characters)

Reviewer #2: My review comments are based on Revision 1 which includes multiple changes made in response to Reviewer 1's feedback. These changes have been substantively met, and no further changes are recommended. As noted by the authors, a well-designed patient-reported outcome measure is an important step in supporting the potential utility of positive health perspectives. The continued development of the CPHQ is an important step in this direction. Future research from independent research groups to clarify the reliability and validity of this measure will be an important outcome that this paper supports.

Thank you for your review of our manuscript and your considerations!

Reviewer #3: Thank you for the opportunity to evaluate this manuscript. In this work, the authors aimed to refine the 32-item Context-sensitive Positive Health Questionnaire (CPHQ) for broader use across healthcare, welfare, and policy domains. Starting from the previously developed CPHQ, they conducted focus group discussions with stakeholders from healthcare, welfare, policy, and research, as well as with patients and citizens, including individuals with lower socioeconomic position. The study combined qualitative feedback and structured item scoring during the focus groups. Participants commented on the relevance, clarity, and formulation of questionnaire items, rated whether items should be retained, adjusted, or omitted, and identified particularly important items. Based on this process, the authors omitted, merged, reformulated, and added items, resulting in a revised set of 28 items. After the focus groups, the revised questionnaire was further examined through expert validation and a member check with a subgroup of original participants. The expert review contributed to additional refinements in item wording and supported the inclusion of some items that had been reconsidered as important for capturing broad health. The final set of items was then categorized according to the Capability Approach into resources, conversion factors, capabilities, and functionings. The final result of the study is a revised 28-item CPHQ, intended as a theory-informed and stakeholder-informed instrument to measure broad health, with future research needed for further validation and possible shortening.

1/ In the Objective sections of both the Abstract and Introduction, the manuscript alternates between “refine the 32-item CPHQ for cross-domain use,” “further improve the CPHQ-instrument,” and broader descriptions of exploring stakeholder perspectives. The manuscript would thus benefit from stating one stable objective formulation and using that exact wording throughout the manuscript to keep the design, analysis, and results aligned.

Thank you for pointing out that we should use one stable formulation to keep everything aligned. We changed the wording in our manuscript to “refine” throughout the entire manuscript.

2/ In the Abstract (“We conducted focus group discussions (9 FGDs; n=76)”) and in the Methods/Table 1, where ten focus groups are listed (“Focus group 1” through “Focus group 10”), it seems that there is a mismatch in the reported number of FGDs. The manuscript would benefit from aligning the number of focus groups consistently across the Abstract, Methods, Results, and tables, and clarifying whether one group was excluded from a specific stage of analysis.

Thank you for addressing this mismatch. We organized ten FGDs but for one of the patient FGDs only one of the patients who agreed to participate showed up. Therefore, we conducted nine FGDs and one interview. We have explained this more in depth in our manuscript to enhance clarity and transparency.

Line 46 in the abstract now states: “We conducted nine focus group discussions (9 (FGDs) and one interview;( n=76) with stakeholders from healthcare, welfare, and policy domains, research, patients and (lower SEP) citizens.”. IN the methods section line 160 in now states: “For one of the FGDs with patients, only one of the patients who agreed to participate showed up. This FGD was therefore approached as an interview using similar questions as the FGDs.” And line 237-238 now states: “As one FGD with patient turned out as an interview, we combined this data with the other patient FGD. This means all patients were analyzed together.”.

3/ In the FGD method subsection, the manuscript states that “Participants in each focus group were divided into groups of no more than five persons,” however no rationale is provided for limiting the discussion groups to five persons, whereas focus group methodology is often described as favoring group sizes closer to 8–12 participants (e.g., Vaughn, 96, Sage Pub), with possible consequences for the breadth of interaction and diversity of viewpoints; The manuscript would benefit from specifying the methodological referential used to justify this choice, and clarifying whether these were considered full focus groups or smaller breakout groups within a larger focus-group session.

The participants focus group discussion groups were divided into smaller sub-groups during part of the focus group discussion. We started and closed with a full group discussion. We limited the number of participants in these sub-groups to a maximum of five participants in order to be able to have sufficient time and opportunity for each individual of the group to state their opinion. Given the fact that posters with statements needed careful consideration, we ensured that every member’s opinion was heard. When a participant was silent, a discussion leader would give that member the floor. Before and after the discussions We are convinced that with the methodology of using smaller sub-groups and starting and concluding in a bigger full group discussion we used all opportunity to capture participants’ opinions and ensured substantiation in the smaller sub-groups while also ensuring that the diversity of opinions was still taken into account in the full focus group.

In the method section of the manuscript we have clarified our choice by:

1) clarifying that we started and closed with a full focus group discussion,

2) by adding clarification about the starting discussion with all participants in line 180-184: “Full group starting discussion: First, the concept of broad health was defined and communicated to participants as: “The extent to which one is capable to adapt and to thrive given one’s physical, mental, social and contextual opportunities” (15). Second, the discussion addressed topics as: what does health consist of, what do we need to capture when measuring health and preconditions of a measuring instrument.“

3) adding two sentences to line 187-191: “Participants in each FGD were divided into approximately equal sub-groups of no more than five persons per sub-group. Each group was accompanied by a researcher that guided the process. The group size was limited to a maximum 5 participants in order to have sufficient time and opportunity for each individual of the group to state their opinion. Silent participants were encouraged to speak up by the researcher.” and

4) by explicating closure with a full group discussion in line 209-212: “Full group closing discussion: After both rounds, the FGD was closed with a discussion among all participants of the FGD to ensure full diversity of opinions was taken into account. The short discussions were mostly about the most striking findings and some additional discussion points related to the preferred number of items and answer method of a measurement instrument.”.

4/ In the Analysis of the FGDs subsection, the thresholds “>50%” and “≤15%” are presented as decision rules for “retain,” “adjust,” and “omit,” but their applicability to this specific focus-group item-refinement setting remains unclear since the rules are borrowed from another consensus context; The manuscript would benefit from adding one concise explanation of why these thresholds were considered suitable for this study design and dataset.

The CPHQ2.0 is intended for use for diverse populations and each opinion during the focus groups was weighed equally. Therefore, you could refer to the use of >50% as a democratic process to ensure a majority decision rather than a plurality. In addition, we used the ≤15% cutoff to identify significant amount of people with a contrasting opinion as the 50% group. We were of the opinion that even if there would be a majority of more than 50%, if there would be a large group opposing that opinion, the reasoning would need to be discussed in the core group rather than immediately make a decision.

In the manuscript we have clarified this in the method section by adding two sentences in line 232-235: ”The >50% cutoff were used to reflect equal weighing of each opinion and a democratic process of majority decision rather than plurality. The ≤15% cutoff was used to identify contrasting opinions compared to the 50% group, in order to investigate, discuss and form a substantiated opinion.”

5/ In the Introduction and Discussion, the manuscript alternates between the terms “broad health,” “Positive Health,” “Context-sensitive Positive Health Questionnaire,” and “Capability Approach,” without always specifying whether these terms refer to the same construct, a framework, or a measurement target; The manuscript would benefit from defining these terms once and using them with stricter consistency throughout.

Wishing you every success in the subsequent editorial stages !

We appreciate the reviewer’s request for stricter consistency to enhance clarity and the support during the editorial stages.

We have reread our manuscript clarified certain terms. In the introduction we adjusted the following:

1)Positive health is defined in the first paragraph of the introduction. Thereafter the Capability approach is defined as a dynamic framework, which does not only look at the outcomes (which is done in Positive Health), but also places health in context of the people’s capability and social and environmental resources. We have indicated this a bit more clearly by adding a couple of words in line 74-75: ”The Capability Approach …..differs from Positive Health as it is …”

2)In the second paragraph in line 85-86 we have added a sentence to clarify that we use the term ‘broad health’ to refer to the broad conceptualization of health which includes the concept of positive health and the capability approach framework.

3)In the third paragraph in line 90-91, three words are added to indicate the section refers to a Positive health questionnaire.

4)The fourth paragraph starts with the explanation of the Context-sensitive Positive Health Questionnaire (CPHQ). In the first sentence in line 104-107 it is explained that the questionnaire is based on both Positive Health and the Capability Approach.

By explicating specific terms in the introduction, we believe that the terms used in the discussion are now clear. We only omitted the term well-being in line 517.

By improving the clarity on the previous paragraphs, we believe this is now consistently defined.

Attachment Submitted filename: response_to_reviewers_auresp_2.docx

# Peer review: Decision Letter 2

**DOI:** 10.1371/journal.pone.0330014.r005

**Authors:** Shiming Hao

20 May 2026

Dear Dr. Boelens,

publication criteria and not, for example, on novelty or perceived impact.

Please submit your revised manuscript by Jul 04 2026 11:59PM. If you will need more time than this to complete your revisions, please reply to this message or contact the journal office at plosone@plos.org. When you're ready to submit your revision, log on to https://www.editorialmanager.com/pone/ and select the 'Submissions Needing Revision' folder to locate your manuscript file.

- A letter that responds to each point raised by the academic editor and reviewer(s). You should upload this letter as a separate file labeled 'Response to Reviewers'.
- A marked-up copy of your manuscript that highlights changes made to the original version. You should upload this as a separate file labeled 'Revised Manuscript with Track Changes'.
- An unmarked version of your revised paper without tracked changes. You should upload this as a separate file labeled 'Manuscript'.

If you would like to make changes to your financial disclosure, please include your updated statement in your cover letter. Guidelines for resubmitting your figure files are available below the reviewer comments at the end of this letter.

If applicable, we recommend that you deposit your laboratory protocols in protocols.io to enhance the reproducibility of your results. Protocols.io assigns your protocol its own identifier (DOI) so that it can be cited independently in the future. For instructions see: https://journals.plos.org/plosone/s/submission-guidelines#loc-laboratory-protocols. Additionally, PLOS ONE offers an option for publishing peer-reviewed Lab Protocol articles, which describe protocols hosted on protocols.io. Read more information on sharing protocols at https://plos.org/protocols?utm_medium=editorial-email&utm_source=authorletters&utm_campaign=protocols.

As the corresponding author, your ORCID iD is verified in the submission system and will appear in the published article. PLOS supports the use of ORCID, and we encourage all coauthors to register for an ORCID iD and use it as well. Please encourage your coauthors to verify their ORCID iD within the submission system before final acceptance, as unverified ORCID iDs will not appear in the published article. *Only*  the individual author can complete the verification step; PLOS staff *cannot*  verify ORCID iDs on behalf of authors.

We look forward to receiving your revised manuscript.

Kind regards,

Shiming Hao, Ph.D.

Guest Editor

PLOS One

Journal Requirements:

1. If the reviewer comments include a recommendation to cite specific previously published works, please review and evaluate these publications to determine whether they are relevant and should be cited. There is no requirement to cite these works unless the editor has indicated otherwise.

2. Please review your reference list to ensure that it is complete and correct. If you have cited papers that have been retracted, please include the rationale for doing so in the manuscript text, or remove these references and replace them with relevant current references. Any changes to the reference list should be mentioned in the rebuttal letter that accompanies your revised manuscript. If you need to cite a retracted article, indicate the article’s retracted status in the References list and also include a citation and full reference for the retraction notice.

Reviewers' comments:

Reviewer's Responses to Questions

**Comments to the Author**

Reviewer #2: All comments have been addressed

Reviewer #3: All comments have been addressed

**********

2. Is the manuscript technically sound, and do the data support the conclusions?

Reviewer #2: Partly

Reviewer #3: Yes

**********

3. Has the statistical analysis been performed appropriately and rigorously?

Reviewer #2: Yes

Reviewer #3: Yes

**********

4. Have the authors made all data underlying the findings in their manuscript fully available?

The PLOS Data policy

Reviewer #2: No

Reviewer #3: Yes

**********

5. Is the manuscript presented in an intelligible fashion and written in standard English?

Reviewer #2: Yes

Reviewer #3: Yes

**********

Reviewer #2: l46 The 'n=76' is misplaced and somewhat confusing as it occur after 'one interview'. If it refers to the number of participants it should go after 'stakeholders'?

ll53 For the 'At the same time, views diverged' lacks clarity as to what happened in these results. Due to a lack of consensus were these items excluded?

l87 Can you specify more clearly what is meant by 'no adequately validated questionnaires' as this is the crux of reasoning for why this study occured. What were the issues with these questionnaires? How does this research seek to address them?

l93 It would be helpful to specify why it was not 'suitable'.

l143 Specify who the 'experts' were.

l154-158 Suggest list these numerically for structure and clarity, as they are currently a long wordy read.

l195 What does 'memo' refer to? Does this mean the CPHQ lists of items?

l207 What is Appendix S3 referring to?

l219 As above - what are the memos referring to?

l217 Clarify how the themes were determined?

l222-232 This decision-making process seems unnecessarily complex and confusing, especially considering it was done per FDG and these groups ranged in size from 6 to 14 participants. Or were all groups combined and then these metrics were calculated?

l322 What type of qualitative analysis was used? it is problematic that this is not clarified throughout the article. Were deductive or inductive approaches used? Who in the team has qualitative experience? Given the background of the core team, there seems to be significant potential for bias to occur- as noted by the authors.

Table 2 - I appreciate why statistically you have used a Median and IQR for Age, but providing a Mean and Age Range would be clearer for the reader.

l331 - missing the word 'see' before Appendix S5

l334 - typo for 'table' should be capitalised.

Table 4 is very difficult to read. Suggest reformat in Landscape not Portrait.

Table 4 - Item 3 (original) is very different to the Revision wording, to the extent it isn't measuring the same thing. What someone values is very different to what is important.

Table 4 - Item 7 Revision seems highly problematic. Unless you have a severe spinal cord injury, everyone can move. The original item that denoted 'easily' was a much better descriptor of what the item seems to be trying to measure.

l345 It would be helpful to specify who the experts were at this stage.

l362 - Should read Table not table

There are some issues with the 'expert' added items. Namely: Item 2 clearly describes achievement - it is not about meaning or belonging. Item 3 is poorly worded and relates to three different things - work, volunteering and 'informal care' which will most likely to jargon to a layperson. Similarly Item 4 sounds like psychological jargon with 'boundaries' and should be rephrased for a layperson.

I also think it is problematic that the 'expert' group came in over the top of the community consultation and added their own preferred items and words in. It would have been more methodologically sound to do this prior to the community consultation, not afterwards.

l381 The comment that the 'experts' felt that the CPHQ should be based on theory hence the use of Capability theory is interesting. Surely the CPHQ is based on Positive Health theory?

l395 Shoud read Table not table 7

Table 7 should be for each of the final items, not only the Totals. The range of ratings for each item should also be provided.

l403 Again should read Table not table 8

l413 It looks like 'times' is an error and should be 'items'.

My comments above tend to be editorial, with some minor methodological issues. Essentially this is a long article on the item (re) development phase of a scale which now needs some good quantitative research done to it. A minor (? major) issue is I think the scale is misnamed. Its not at all about the contexts within which patients life their lives and deal with it's challenges. It is, in fact, entirely without context in the items, and the capability framework is about linguistic expression of capabilities, it is not at all about context. Given these concerns though, positive health is an important, slowly emerging concept, and a good scale to measure it well is a worthy endeavour.

Reviewer #3: The authors carried out a very thorough revision and I am particularly satisfied with the final state of the manuscript.

**********

what does this mean?). If published, this will include your full peer review and any attached files.

If you choose “no”, your identity will remain anonymous but your review may still be made public.

**Do you want your identity to be public for this peer review?**  For information about this choice, including consent withdrawal, please see our Privacy Policy

Reviewer #2: No

Reviewer #3: **Yes:** Dr. Arthur Trognon

**********

[NOTE: If reviewer comments were submitted as an attachment file, they will be attached to this email and accessible via the submission site. Please log into your account, locate the manuscript record, and check for the action link "View Attachments". If this link does not appear, there are no attachment files.]

To ensure your figures meet our technical requirements, please review our figure guidelines: https://journals.plos.org/plosone/s/figures

You may also use PLOS’s free figure tool, NAAS, to help you prepare publication quality figures: https://journals.plos.org/plosone/s/figures#loc-tools-for-figure-preparation.

NAAS will assess whether your figures meet our technical requirements by comparing each figure against our figure specifications.

# Author comment: Author response to Decision Letter 3

**DOI:** 10.1371/journal.pone.0330014.r006

25 Jun 2026

Rebuttal letter: PONE-D-25-39699

Stakeholder and end-user perspectives to improve the Context-sensitive Positive Health Questionnaire (CPHQ) to measure broad health

Dear editors and reviewers,

We would like to thank you for your constructive feedback on our manuscript. We appreciate the time and effort that you took to review our manuscript. We have carefully considered all your comments and revised our manuscript accordingly. We believe that these revisions improved the clarity and quality of our manuscript. Below, we provide a point-by-point response to each comment. Reviewer and editor comments are in black font color followed by our response and a description of our changes in blue. We hope that the revised version meets your expectations and we look forward to your response.

On behalf of all authors,

Cheryl Roumen and Mirte Boelens

Journal Requirements:

1. If the reviewer comments include a recommendation to cite specific previously published works, please review and evaluate these publications to determine whether they are relevant and should be cited. There is no requirement to cite these works unless the editor has indicated otherwise.

2. Please review your reference list to ensure that it is complete and correct. If you have cited papers that have been retracted, please include the rationale for doing so in the manuscript text, or remove these references and replace them with relevant current references. Any changes to the reference list should be mentioned in the rebuttal letter that accompanies your revised manuscript. If you need to cite a retracted article, indicate the article’s retracted status in the References list and also include a citation and full reference for the retraction notice.

Reviewers' comments:

Comments to the Author

1. If the authors have adequately addressed your comments raised in a previous round of review and you feel that this manuscript is now acceptable for publication, you may indicate that here to bypass the “Comments to the Author” section, enter your conflict of interest statement in the “Confidential to Editor” section, and submit your "Accept" recommendation.

Reviewer #2: All comments have been addressed

Reviewer #3: All comments have been addressed

________________________________________

2. Is the manuscript technically sound, and do the data support the conclusions?

The manuscript must describe a technically sound piece of scientific research with data that supports the conclusions. Experiments must have been conducted rigorously, with appropriate controls, replication, and sample sizes. The conclusions must be drawn appropriately based on the data presented.

Reviewer #2: Partly

Reviewer #3: Yes

________________________________________

3. Has the statistical analysis been performed appropriately and rigorously?

Reviewer #2: Yes

Reviewer #3: Yes

________________________________________

4. Have the authors made all data underlying the findings in their manuscript fully available?

The PLOS Data policy requires authors to make all data underlying the findings described in their manuscript fully available without restriction, with rare exception (please refer to the Data Availability Statement in the manuscript PDF file). The data should be provided as part of the manuscript or its supporting information, or deposited to a public repository. For example, in addition to summary statistics, the data points behind means, medians and variance measures should be available. If there are restrictions on publicly sharing data—e.g. participant privacy or use of data from a third party—those must be specified.

Reviewer #2: No

Reviewer #3: Yes

________________________________________

5. Is the manuscript presented in an intelligible fashion and written in standard English?

PLOS ONE does not copyedit accepted manuscripts, so the language in submitted articles must be clear, correct, and unambiguous. Any typographical or grammatical errors should be corrected at revision, so please note any specific errors here.

Reviewer #2: Yes

Reviewer #3: Yes

________________________________________

6. Review Comments to the Author

Reviewer #2:

l46 The 'n=76' is misplaced and somewhat confusing as it occur after 'one interview'. If it refers to the number of participants it should go after 'stakeholders'?

We adjusted this accordingly.

ll53 For the 'At the same time, views diverged' lacks clarity as to what happened in these results. Due to a lack of consensus were these items excluded?

Thank you for pointing out that our phrasing was unclear. We have clarified this sentence in the abstract by explicitly stating that the differing views did not automatically lead to omission of the items but that these final items were based on quantitative scoring and qualitative feedback and the core group discussions.

l87 Can you specify more clearly what is meant by 'no adequately validated questionnaires' as this is the crux of reasoning for why this study occured. What were the issues with these questionnaires? How does this research seek to address them?

Thank you for highlighting this. Existing broad-health questionnaires either lack sufficient psychometric validation, were developed primarily as dialogue tools rather than measurement instruments and do not adequately include contextual factors such as social, political or environmental influences on health. To illustrate, one of the first instruments was the 42-item Positive Health questionnaire which lacks comprehensibility and discriminant validity(1, 2). It was also not developed as a measuring instrument but as a dialogue tool. A shorter version, the 17-item Positive Health questionnaire showed improved psychometric properties but the validity and reliability of the constructs underlying the questionnaire has not yet sufficiently been shown. Moreover, the IPH-17 is primarily focused on perceived health whereas contextual factors are also important(2). Our study aims to address these limitations by refining a questionnaire that is theoretically grounded in both Positive Health and the Capability Approach and includes contextual determinants of health. This is explained in the paragraph below.

We agree that this was not entirely clear in our introduction. We reformulated some sentences in our introduction and feel that it is now sufficiently clear.

l93 It would be helpful to specify why it was not 'suitable'.

We have specified this accordingly. It was not suitable as it lacks comprehensibility and also in discriminant validity.

l143 Specify who the 'experts' were.

We agree that this is not entirely clear as we explain this later in the manuscript. We added a description about the experts. The experts include the core group and expert from the Dutch National Positive Health research Network. This network covers a broad range of researchers and we now specify this in the manuscript.

We specified this at the start of the method section in l138-147: The core group (MS, JK, MB, CR, EB, JD) consisted of six project members with diverse research backgrounds: a researcher with a background in psychology and statistics, a researcher with a background in population health, a researcher in the field of broad health concepts, a researcher in the field of health behavior, health promotion and public health, a researcher with a background in nutritional sciences and public health, and a researcher with a background in participatory research and shared decision-making.

The Dutch National Positive Health research Network includes researchers from several Dutch universities, hospitals, the National Institute of Public Health and the Environment, the Institute of Positive Health, and VILANS a knowledge organization for care and support

l154-158 Suggest list these numerically for structure and clarity, as they are currently a long wordy read.

Thank you for this suggestion. We agree that the description of the stakeholder groups was lengthy and difficult to read. We have revised this by presenting the stakeholders in a more structured format. We also corrected a minor error in table 1.

l195 What does 'memo' refer to? Does this mean the CPHQ lists of items?

We agree that this may not entirely clear. A memo refers to a sticky note on which participants of the FGDs wrote their qualitative feedback concerning the individual CPHQ items. We changed the word in the manuscript to sticky notes.

l207 What is Appendix S3 referring to?

It refers to the example prompts (topic list) used for the FGDs. We rephrased the sentence for to improve clarity and readability.

l219 As above - what are the memos referring to?

A memo refers to a sticky note. We changed the word in the manuscript to sticky notes. The sticky notes included qualitative feedback discussed by the focus group members about individual CPHQ items. We specified this in the manuscript (l255 and l258).

l217 Clarify how the themes were determined?

Thank you for addressing tat this important aspect is not clear. We have clarified the analytical approach used to identify the themes. The themes were explored through deductive and inductive qualitative content analysis off both the focus group transcripts and the participant feedback on the sticky notes. The analysis focused specifically on feedback regarding the questionnaire items (e.g., wording, overlap and missing content) rather than on generating a broader theory of health.

It now states (L 243-248): FGDs were transcribed ad verbatim and analyzed independently by two researchers (CR and MB). Qualitative feedback on the sticky notes and the transcripts were analyzed deductively on the following themes: formulation, overlap, redundancy, importance, new suggestions, duration and length of the questionnaire and inductively using content analysis. Analysis of the feedback on sticky notes for was done using an adapted consensus method inspired by the COMET handbook and Blazeby et al.(3, 4).

l222-232 This decision-making process seems unnecessarily complex and confusing, especially considering it was done per FDG and these groups ranged in size from 6 to 14 participants. Or were all groups combined and then these metrics were calculated?

Thank you for this comment. We agree that the decision-making procedure was not sufficiently clear. Quantitative scoring was first calculated separately for each focus group. Subsequently, the results (e.g,, omit, adjust, retain) were aggregated to determine whether overall consensus had been reached. Items for which overall consensus was reached were retained, adjusted or omitted accordingly. For items where no overall consensus was reached, the core group reviewed the quantitative results together with the qualitative feedback from the transcripts and and the sticky notes and the golden ticket prioritization to reach a final decision. This procedure enabled us to identify variations between focus groups. We have clarified this in the manuscript and thoroughly rewrote this section, see l238-290: Analysis of the FGDs

Descriptive analyses were performed for educational level, gender and work experience using IBM SPSS statistics for Windows, version 29.0 (International Business Machines Corporation, Armonk, New York).

FGDs were transcribed ad verbatim and analyzed independently by two researchers (CR and MB). Qualitative feedback on the sticky notes and the transcripts were analyzed deductively on the following themes: formulation, overlap, redundancy, importance, new suggestions, duration and length of the questionnaire and inductively using content analysis. Analysis of the feedback on sticky notes for was done using an adapted consensus method inspired by the COMET handbook and Blazeby et al.(3, 4).

For each FGD all items were scored quantitatively by counting the colored stickers scored as 1 for green, 2 for yellow and 3 for orange. Number of golden tickets was scored per item. Decision rules were applied per item separately for each FGD. An item was retained when >50% scored 1 (green) and ≤15% 3 (orange), adjusted when >50% scored 2 (yellow) and ≤15% 1 (green), and omitted when >50% scored 3 (orange) and ≤15% 1 (green). The >50% cutoffs were used to reflect equal weighing of each opinion and a democratic process of majority decision rather than plurality. The ≤15% cutoff was used to identify contrasting opinions compared to the 50% group, in order to investigate, discuss and form a substantiated opinion.

If an item score fell outside these predefined categories, the quantitative result was considered inconclusive and the item was subsequently discussed by the core group together using the quantitative results together with the qualitative input from the sticky notes and transcripts. When the quantitative results were close to one of the predefined categories (retain, adjust or omit), this was taken into consideration during discussion, but did not automatically determine the final decision. Final decisions were reached through consensus within the core group.

As one FGD with patients turned out as an interview, we combined this data with the other patient FGD data. This means all patients were analyzed together.

The resulting scores (retain, adjust and omit) were subsequently compared across all FGDs to determine whether consensus between FGDs had been reached. Consensus was defined as all FGDs resulting in the same quantitative decision (retain, adjust or omit) for a given item. In these cases, the consensus decision was followed by the core group. When decisions differed between FGDs, the item was discussed by the core group using the quantitative results, qualitative feedback from the transcripts and sticky notes and the golden ticket prioritization.

The goal of the qualitative feedback was not to examine emerging themes but rather to examine which aspects of the individual CPHQ items required adaptation or adjustment or removal. Therefore, all qualitative feedback was grouped all per individual CPHQ item across FGDs. Feedback was summarized by examining the frequency of recurring comments, for example that an item was difficult to understand or should be reformulated.

Items without consensus between FGDs were discussed by the core group. Consensus was achieved through structured discussion until agreement was reached. For each item, a different core group member initiated the discussion by stating their preferred decision and rationale. Subsequently, the remaining members shared their views in a rotating order. At least four out of the six core group members had to agree on a decision to retain, adjust or omit, merge an item.. This structured discussion format was deliberately applied to ensure that all core group members had equal opportunity to contribute and to mitigate potential dominance effects or power imbalances within the group. CR and MB facilitated the discussion.

Items classified as ‘adjust’ were reformulated based on the qualitative feedback. This could also include merging items together.

Finally items that were retained, adjusted and sometimes merged were further refined to improve readability, consistency and comprehensibility across the questionnaire.

Step 1 led to a revised CPHQ with a reduced set of items.

l322 What type of qualitative analysis was used? it is problematic that this is not clarified throughout the article. Were deductive or inductive approaches used? Who in the team has qualitative experience? Given the background of the core team, there seems to be significant potential for bias to occur- as noted by the authors.

First of all, we agree that the qualitative approach should have been described more explicitly. We have clarified that we applied a deductive and inductive content analysis. We deductively explored the following themes formulation, overlap, redundancy, importance, new suggestions, duration and length of the questionnaire. Our manuscript now states (l243-248): FGDs were transcribed ad verbatim and analyzed independently by two researchers (CR and MB). Qualitative feedback on the sticky notes and the transcripts were analyzed deductively on the following themes: formulation, overlap, redundancy, importance, new suggestions, duration and length of the questionnaire and inductively using content

Attachment Submitted filename: Rresponse to reviewers.docx

# Editor report: Decision Letter 3

**DOI:** 10.1371/journal.pone.0330014.r007

**Authors:** Shiming Hao

6 Aug 2026

Stakeholder and end-user perspectives to refine the Context-sensitive Positive Health Questionnaire (CPHQ) to measure broad health

PONE-D-25-39699R3

Dear Dr. Mirte Boelens,

We’re pleased to inform you that your manuscript has been judged scientifically suitable for publication and will be formally accepted for publication once it meets all outstanding technical requirements.

Within one week, you’ll receive an e-mail detailing the required amendments. When these have been addressed, you’ll receive a formal acceptance letter and your manuscript will be scheduled for publication.

An invoice will be generated when your article is formally accepted. Please note, if your institution has a publishing partnership with PLOS and your article meets the relevant criteria, all or part of your publication costs will be covered. Please make sure your user information is up-to-date by logging into Editorial Manager at Editorial Manager® and clicking the ‘Update My Information' link at the top of the page. For questions related to billing, please contact billing support.

If your institution or institutions have a press office, please notify them about your upcoming paper to help maximize its impact. If they’ll be preparing press materials, please inform our press team as soon as possible -- no later than 48 hours after receiving the formal acceptance. Your manuscript will remain under strict press embargo until 2 pm Eastern Time on the date of publication. For more information, please contact onepress@plos.org.

Kind regards,

Shiming Hao, Ph.D.

Guest Editor

PLOS One

# Editor report: Acceptance letter

**DOI:** 10.1371/journal.pone.0330014.r008

**Authors:** Shiming Hao

PONE-D-25-39699R3

PLOS One

Dear Dr. Boelens,

I'm pleased to inform you that your manuscript has been deemed suitable for publication in PLOS One. Congratulations! Your manuscript is now being handed over to our production team.

At this stage, our production department will prepare your paper for publication. This includes ensuring the following:

* All references, tables, and figures are properly cited

* All relevant supporting information is included in the manuscript submission,

* There are no issues that prevent the paper from being properly typeset

You will receive further instructions from the production team, including instructions on how to review your proof when it is ready. Please keep in mind that we are working through a large volume of accepted articles, so please give us a few days to review your paper and let you know the next and final steps.

Lastly, if your institution or institutions have a press office, please let them know about your upcoming paper now to help maximize its impact. If they'll be preparing press materials, please inform our press team within the next 48 hours. Your manuscript will remain under strict press embargo until 2 pm Eastern Time on the date of publication. For more information, please contact onepress@plos.org.

You will receive an invoice from PLOS for your publication fee after your manuscript has reached the completed accept phase. If you receive an email requesting payment before acceptance or for any other service, this may be a phishing scheme. Learn how to identify phishing emails and protect your accounts at https://explore.plos.org/phishing.

If we can help with anything else, please email us at customercare@plos.org.

Thank you for submitting your work to PLOS One and supporting open access.

Kind regards,

PLOS ONE Editorial Office Staff

on behalf of

Professor Shiming Hao

Guest Editor

PLOS One
